# It's not just the size that matters: crystal engineering of lanthanide-based coordination polymers†

**DOI:** 10.1039/d3sc03746k

**Published:** 2023-12-20

**Authors:** Adrian Hauser, Luca Münzfeld, Cedric Uhlmann, Sergei Lebedkin, Sören Schlittenhardt, Ting-Ting Ruan, Manfred M. Kappes, Mario Ruben, Peter W. Roesky

**Affiliations:** a Institute of Inorganic Chemistry, Karlsruhe Institute of Technology (KIT) Engesserstraße 15 D-76131 Karlsruhe Germany roesky@kit.edu; b Institute of Nanotechnology, Karlsruhe Institute of Technology (KIT) Hermann-von-Helmholtz-Platz 1 D-76344 Eggenstein-Leopoldshafen Germany; c Institute of Physical Chemistry, Karlsruhe Institute of Technology (KIT) Fritz-Haber-Weg 2 D-76131 Karlsruhe Germany; d Centre Européen de Science Quantique (CESQ), Institut de Science et d’Ingénierie Supramoléculaires (ISIS, UMR 7006), CNRS-Université de Strasbourg 8 allée Gaspard Monge BP 70028 67083 Strasbourg Cedex France; e Institute of Quantum Materials and Technologies (IQMT), Karlsruhe Institute of Technology Hermann-von-Helmholtz-Platz 1 76344 Eggenstein-Leopoldshafen Germany

## Abstract

Synthesis and characterization of Lewis base free coordination polymers of selected lanthanides are presented. For this purpose, the substituted Cot^TIPS^ ligand (Cot^TIPS^ = 1,4-bis-triisopropylsilyl-cyclo-octatetraendiide) was used to synthesize homoleptic, anionic multidecker compounds of the type [K{Ln^III^(ɳ^8^-Cot^TIPS^)_2_}]_*n*_. Depending on the solvent used for crystallization and the ionic radii of the lanthanide cations, three different categories of one-dimensional heterobimetallic coordination polymers were obtained in the solid state. For the early lanthanides La and Ce a unique helical conformation was obtained by crystallization from toluene, while the ionic radius of Pr seems to be a turning point towards the crystallization of zigzag polymers. For Er a third structural motif, a trapezoidal wave polymer was observed. Additionally, the zigzag polymer for all compounds could be obtained by changing the solvent from toluene to Et_2_O, reavealing a correlation between solid-state structure and ionic radii as well as solvent. While photoluminescence (PL) properties of Cot-lanthanide compounds are scarce, the La complexes show ligand centered green luminescence, whereas the Ce complexes reveal deep red emission origin from d–f transitions. The Er-compounds are single-molecule magnets, in which the magnetic relaxation of each Er ion occurs isolated from its neighbors at temperatures above 10 K, while below 9 K a strong antiferromagnetic coupling between the Er ions was seen.

## Abstract 2

The synthesis of various Cot^TIPS^-based Ln coordination polymers is reported. Different chain polymers were obtained depending on the ionic radii of the lanthanides and the solvent chosen for crystallization. The photophysical and magnetic properties were investigated.

## Graphical abstract

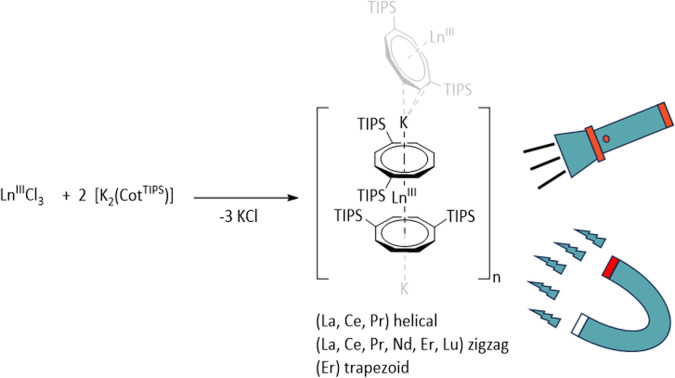

## Introduction

The Cot dianion (Cot = cyclooctatetraendiide) and its derivatives are some of the most prominent ligands in lanthanide chemistry. They not only form homoleptic but also heteroleptic sandwich compounds.^1–16^ Nevertheless, few fundamental structural motifs have been established, reflecting the limited number of successful complete structural characterization studies.^17^ The reason for this is the low solubility of the unsubstituted Cot ligand and its complexes in common organic solvents, which complicates the crystallization of these compounds as well as further investigations regarding their physical properties and reactivities.^2^ The classical anionic sandwich complexes of the general type [K(L)_n_][Ln^III^(η^8^-Cot)_2_] (L = thf (*n* = 3), L = 18-crown-6 (*n* = 1), L = diglyme (*n* = 1)) were among the first sandwich compounds of the lanthanides to be isolated.^2,18,19^ Due to their simple structure, their physical properties as single-molecule magnets (SMMs), luminescent compounds, as well as their sometimes high reactivity in redox transformations, are of principle interest in current research.^6,7,19–24^ However, due to the ionic character of these compounds the corresponding sandwich ions are only soluble in N- or O-donating solvents and were so far only crystallized in the presence of ether.^5,7,18,19,21,25^ Thus, it is almost impossible to analyze these species as defined compounds by single crystal X-ray diffraction (SCXRD) in the absence of etheric solvents, crown ethers, or cryptands that stabilize the alkali metal cations. Nevertheless, it was possible to crystallize the solvate [Li(η^2^-dme)Tb(η^8^-Cotʺ)_2_]_*n*_ (dme = 1,2-dimethoxyethane), as a lithium-bridged polymer from *n*-pentane.^26^ The defined formation of such polymeric structures in donor-free environments in particular could lead to novel macromolecular structural motifs with interesting properties.

One approach to increase the solubility of COT ligands developed in the 1990s was the introduction of bis- and tris-Me_3_Si-substituted ligands (1,4-(Me_3_Si)_3_C_8_H_6_ = COT′′ and 1,3,6-(Me_3_Si)_3_C_8_H_5_ = COT′′′) in lanthanide chemistry. This alteration of the ligand increased the number of lanthanide COT complexes significantly.^9,10,22,27–30^ The use of the bis-triisopropylsilyl-substituted Cot^TIPS^ ligand (Cot^TIPS^ = 1,4-bis-triisopropylsilyl-cyclo-octatetraendiide) as ligand in the coordination sphere of lanthanides was a further logical step.^31^ It has already been shown to be fruitful for the design of novel lanthanide compounds and the fine-tuning of their physical properties,^12,13,31^ due to the increased solubility in common organic solvents as well as sufficient steric shielding. Therefore, we have applied this ligand for the synthesis of Lewis base free coordination polymers. In this context, we recently communicated for the divalent metals Sr, Sm, Eu the isolation and structural characterization of the cyclocene family [*cyclo*-M^II^(μ-η^8^:η^8^-Cot^TIPS^)]_18_ (M = Sr, Sm, Eu).^32^ These cyclic compounds consist of eighteen repeating units, forming almost ideally circular, closed rings in the solid state. The bending seen in the cyclocene structures is caused by the steric demand of the Cot^TIPS^ ligands. The driving force for the ring formation is the energy gained by its closure. Moreover, we reported the Sm^III^-coordination polymer [K{Sm^III^(ɳ^8^-Cot^TIPS^)_2_}]_*n*_, which forms a zigzag-type polymer chain which is bent at the potassium atoms.^13^ Herein, we now present a full account of the lanthanide polymers of composition [K{Ln^III^(ɳ^8^-Cot^TIPS^)_2_}]_*n*_. We have investigated the photophysical and magnetic properties of selected compounds and show that in dependence of the ion radius of the center metal either zigzag-type, helix-type, or trapezoid polymers are formed.

## Results and discussion

### Synthesis and structural characterization

**Scheme 1:**
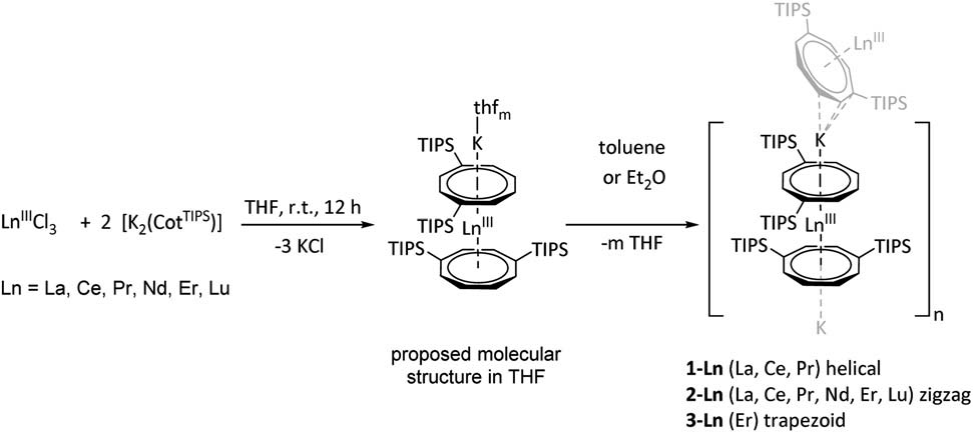
Synthesis of [K{Ln^III^(ɳ^8^-Cot^TIPS^)_2_}]_n_, compounds 1-Ln to 3-Ln.

The desired polymers [K{Ln^III^(ɳ^8^-Cot^TIPS^)_2_}]_*n*_ were synthesized by salt elimination reaction between two equivalents of [K_2_(Cot^TIPS^)] and the corresponding lanthanide halides LnCl_3_ (Ln = La, Ce, Pr, Nd, Er and Lu) in THF (Scheme 1).^13^

In contrast to the unsubstituted analogues, the crude products obtained could be subsequently extracted with hot toluene and isolated as crystalline solids of the type [K{Ln^III^(ɳ^8^-Cot^TIPS^)_2_}]_*n*_ upon cooling to room temperature. The obtained compounds show different macromolecular structures in the solid state, depending on the ionic radius of the respective lanthanide. Hence, for the early and thus larger lanthanides La, Ce, and Pr, crystallization from hot toluene leads to a one-dimensional coordination polymer which adopts a helical configuration. It should be noted that helical structures of lanthanide compounds have also be seen before, *e.g.* in anionic cerium(iii) corrole complexes.^33^

**Fig. 1:**
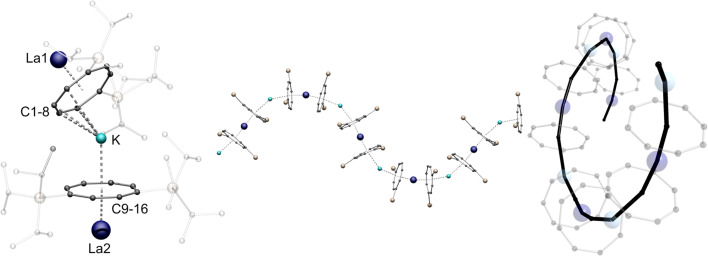
Left: molecular structure of the asymmetric unit of 1-La. Middle: section of the macromolecular helical structural motif of 1-La. TIPS-groups are transparent and hydrogen atoms are omitted for clarity Right: top view of the helical polymer. Colour code; La dark blue, K blue, Si yellow, C black.

Fig. 1 shows exemplarily both the asymmetric unit and a section of the helical structure of 1-La in the solid state. The helical polymer 1-La crystallizes in the orthorhombic, acentric space group *C*222_1_, in reproducibly enantiomerically pure form (Fig. 1, right, shows the right-handed helix). Here, the asymmetric unit contains a [(η^4^-Cot^TIPS^)K(η^4^-Cot^TIPS^)]^3–^ fragment coordinated on both sides by formally half-occupied, crystallographically distinguishable La^III^-cations (Fig. 1, left). The La-Ct_Cot_ distances (Ct_Cot_ = centroid of the Cot ring) in the two [La^III^(η^8^-Cot^TIPS^)]^+^ fragments are almost identical despite the different identities of the La^III^-cations (2.1249(1) Å and 2.1245(1) Å). This value is slightly increased compared to the related compound [Li(thf)_3_La^III^(η^8^-Cot)_2_], probably due to the increased steric demand of the TIPS groups contrary to the unsubstituted Cot ligand (2.068 Å and 2.106 Å).^34^ The K–Ct_Cot_ distances diverge significantly from each other with 2.684(2) Å for K-Ct_C1-8_ and 2.855(2) Å for K-Ct_C9-16_, as a result of different formal coordination numbers. The more distant ligand is laterally shifted from the neighboring La-Ct-K axis, inducing rotation of the helical structure. The bending of the structure occurs mainly at the potassium positions with a Ct_Cot_-K-Ct_Cot_ angle of 149.98(7)°, while the [(Cot^TIPS^)_2_La^III^]^−^ fragments show a more coplanar coordination geometry with Ct_Cot_-La-Ct_Cot_ angles of 172.010(1)° and 166.215(1)°. It should be noted here that the open side of the potassium ion is sterically shielded by one TIPS group of the η^4^-Cot^TIPS^ ligand. The helical chain can be further explained by the unequal La^III^ positions: Due to their inequivalence, two different K-La1-K′'-La2 torsion angles were determined: 81.88(10)° and −4.18(13)°. Accordingly, within one repetition of the polymer, a twist of 86° occurs in the polymer chain. About four monomeric units are needed for a complete 360° twist.

^1^H NMR spectroscopic investigations in THF-*d*_8_ revealed one set of signals for twelve aromatic protons of the Cot-ligands between *δ* = 6.28–6.06 ppm, making them equivalent in solution. The *iso*-propyl groups of two silyl-substituents show one septet at *δ* = 1.51 ppm and two sets of doublets at *δ* = 1.19 ppm and *δ* = 1.16 ppm. In the ^29^Si{^1^H} NMR spectrum one singlet at *δ* = 4.5 ppm is observed. The asymmetric unit, as well as a section of the analogous helix structure of the cerium compound 1-Ce, is shown in the ESI in Fig. S23.†

**Fig. 2:**
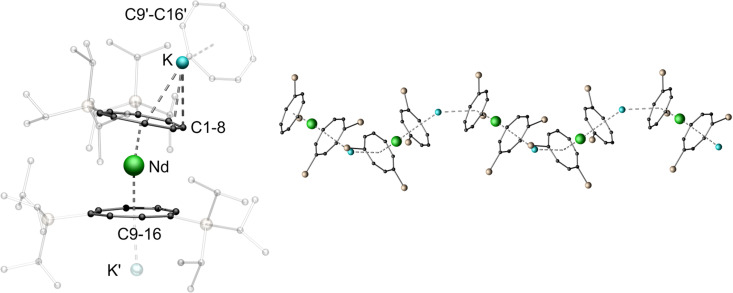
Left: molecular structure of the asymmetric unit of 2-Nd. Right: section of the macromolecular zigzag structural motif of 2-Nd. TIPS-groups are transparent and hydrogen atoms are omitted for clarity. Colour code; Nd green, K blue, Si yellow, C black.

Apparently, the corresponding compound of the next smaller lanthanide Pr plays a pivotal role, since both a helical chain polymer (1-Pr, Fig. S24†) and a one-dimensional zigzag polymer (2-Pr, Fig. S25†) can be obtained in an identical crystallization approach *i.e.*, from a hot toluene solution. A mixture of crystals from both isomers, which were analyzed by SCXRD, is formed in each case. This suggests that the ionic radius of Pr^III^ represents a turning point in the preferential crystallization of the helical structure towards the zigzag polymer. To confirm this assumption, the synthesis of the analogous Nd^III^-compound 2-Nd was pursued. Based on the previously gained insights, a zigzag shaped polymer chain structure was expected for 2-Nd. SCXRD measurements of the obtained crystals indeed revealed this to be the isomorphic structural motif of 2-Pr. Analogous to the previously reported Sm^III^-compound, 2-Nd crystallizes in the monoclinic space group *P*2_1_/*n*.^13^ In this case, the asymmetric unit is formed by a [Nd^III^(η^8^-Cot^TIPS^)_2_]^−^ fragment coordinated by a potassium ion (Fig. 2). In the macromolecular structure, the linkage of the monomeric units through the potassium ions occurs in a μ-η^3^:η^8^ coordination mode. Binding to the η^8^-Cot^TIPS^ ligand arises with a K-Ct_η8Cot_ distance of 2.4782(11) Å, while the second, η^3^ allyl-like coordinated ligand shows a K-Ct_η3-Cot_ distance of 3.2356(10) Å. As seen in 1-La, the bending of the polymer chain at the potassium positions occurs with a Ct-K-Ct angle of 148.81(4)°. Since 2-Nd has no independent lanthanide positions, the K-Nd-K′-Nd′ torsion angles are identical. Accordingly, no rotation of the polymer chain is observed. Consequently, instead of the helical structure of 1-La and 1-Ce, the zigzag motif is formed (Fig. 2, right). As expected, the Nd^III^ cations are coordinated by two η^8^-Cot^TIPS^ ligands. The Nd-Ct_Cot_ distances are 1.9986(4) Å and 2.0385(4) Å and the Ct-Nd-Ct bending angle of the fragment is 170.50(2)°.

**Fig. 3:**
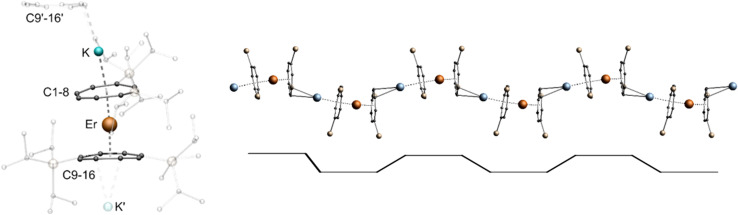
Left: molecular structure of the asymmetric unit of 3-Er. Right: section of the macromolecular trapezoidal wave motif of 3-Er (top), schematic trapezoidal wave (bottom), TIPS-groups are transparent and hydrogen atoms are omitted for clarity. Colour code; Er orange, K blue, Si yellow, C black.

^1^H NMR spectroscopic investigations of compound 2-Nd in THF-*d*_8_ revealed two broad signals for the TIPS-groups at *δ* = 1.23 ppm and *δ* = 0.88 ppm, while no resonance was observed for the ring protons due to the paramagnetic nature of the compound. In the ^29^Si{^1^H} spectrum a pronounced paramagnetic shift of the resonance towards *δ* = −45.5 ppm was detected. To further investigate the influence of the lanthanide ionic radius upon the structural motif obtained, the synthesis was carried out with the even smaller Er^III^-cation (Scheme 1).^35^ Concomitantly, this opened the possibility to investigate magnetic properties of such coordination polymers, since Er^III^ Cot-complexes have been shown to exhibit SMM properties.^36,37^ Compound 3-Er crystallizes in the orthorhombic acentric space group *F*2*dd* as a staggered coordination polymer similar to 2-Ln. However, its macromolecular structure is reminiscent of a trapezoidal wave and is thus representing a third member in the series of Ln^III^-Cot^TIPS^-polymers. As observed in 2-Ln, the asymmetric unit consists of an [Er^III^(η^8^-Cot^TIPS^)_2_]^−^ fragment, which is linked by the potassium ions in a μ-η^2^:η^8^ coordination mode in the polymeric structure (Fig. 3). The principal difference between the structures of 2-Ln and 3-Er is precisely the μ-η^2^:η^8^ bridging mode. The potassium ion is so strongly displaced against the η^2^-bound ligand that there is no bending of the repeat units against each other at this point. Consequently, the consecutive [Er^III^(η^8^-Cot^TIPS^)_2_]^−^ units are aligned almost parallel to each other. The binding metrics in this fragment are, within the expected deviations of decreasing ionic radii, similar to the previously discussed compounds. The Er-Ct distances are 1.8679(6) Å as well as 1.9405(6) Å, which is slightly elongated compared to Er-Ct distances in the unsubstituted Er-compound [K(thf)_3_Er^III^(Cot)_2_].^19^ The Ct-Er-Ct angle of 173.62(3)° illustrates the tilted coordination of the Cot^TIPS^ moieties similar to the neutral [(Cot)Er^III^(Cp*)] (Cp* = pentamethylcylopentadienyl) sandwich complex.^38^

**Fig. 4:**
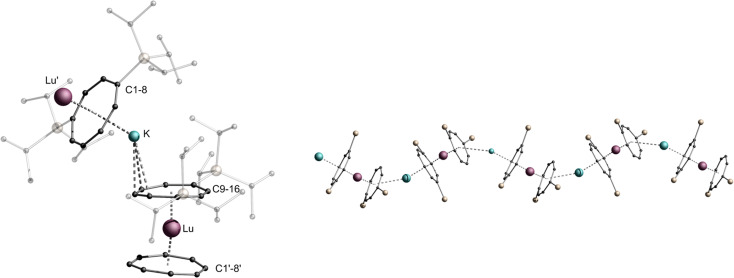
Left: molecular structure of the asymmetric unit of 2-Lu. Right: section of the macromolecular zigzag motif of 2-Lu, TIPS-groups are transparent and hydrogen atoms are omitted for clarity. Colour code; Lu purple, K blue, Si yellow, C black.

However, the size of the respective lanthanide cation is not the only determining factor in the formation of the one-dimensional polymeric chain. The solvent used for crystallization also plays a crucial role. Whereas the compounds mentioned so far (1-La, 1-Ce, 1-Pr, 2-Pr, 2-Nd and 3-Er) were crystallized exclusively from toluene, changing the solvent used for crystallization to Et_2_O allowed the isolation of isostructural zigzag structures for La (2-La), Ce (2-Ce) and Er (2-Er) (Fig. S28–S30†). Under these conditions, the zigzag structural 2-Pr isomer and not a mixture was isolated for Pr. All bonding parameters are almost identical to the other zigzag coordination polymers (2-Ln) with deviations induced by the changing ionic radii. In the case of Lu, it was not possible to crystallize the compound from toluene. Instead, single crystals of the compound were obtained from Et_2_O only, leading to the formation of the zigzag polymer 2-Lu (Fig. 4, structural parameters are given in the ESI page S32†). Varying the solvent for the compounds that did not crystallize as a helical coordination polymer due to their ionic radii (2-Nd, 2-Pr) did not result in a different macromolecular structural motif.

### Photophysical properties

**Fig. 5:**
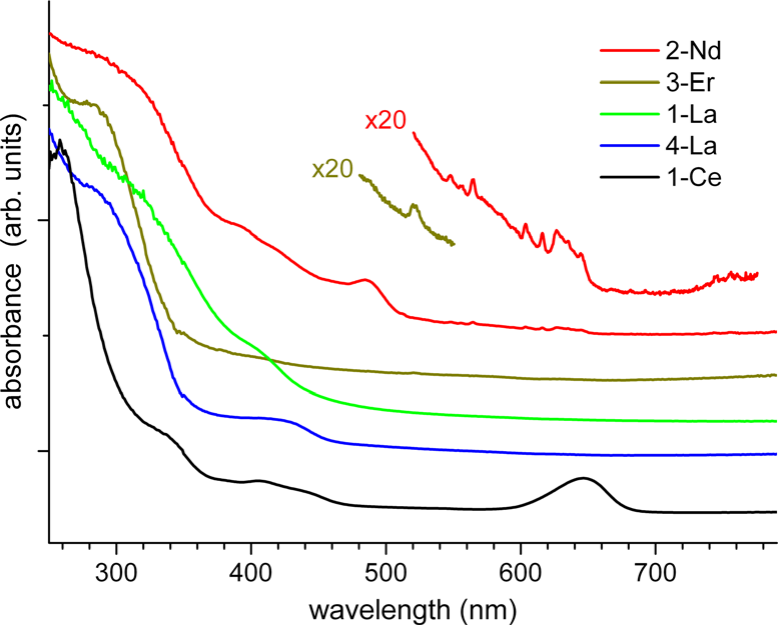
Absorption spectra of polycrystalline 1-Ce, 1-La, 2-La, 2-Nd, and 3-Er at ambient temperature. The spectra are vertically shifted for clarity.

For detailed electronic spectroscopy studies we selected 1,2-Ce, 1,2-La, 2-Nd and 3-Er. Manifold photophysical properties were found, reflecting primarily the various attributes of the lanthanide ions in these coordination polymers. Fig. 5 shows UV-Vis absorbance spectra of the polycrystalline samples measured in an integrating sphere at ambient temperature (for experimental details see the SI†). Except for the bands of 1-Ce at 650 nm (a very similar spectrum of 2-Ce is not shown) and 2-Nd at 480 nm, the major absorption begins in common at about 460 nm and increases below 350 nm, in accordance with the visual appearance of 1,2-La, 2-Nd and 3-Er as yellow crystallite powders. This absorption can be attributed to Cot^TIPS^ intraligand and charge transfer transitions. A prominent band of 1,2-Ce at 650 nm, which lends the cerium compounds an intense green colour, is assigned to the ^2^F_5/2_ → ^2^D f–d transition in Ce^III^ cations (from the ground to the lowest excited state).^39^ In contrast, f–f transitions of Nd^III^ and Er^III^ are of low intensity (symmetry-forbidden) and only observed as small narrow peaks in the magnified spectra of 2-Nd and 3-Er (Fig. 5). For instance, a peak at 522 nm in the spectrum of 3-Er can be assigned to the ^4^I_15/2_ → ^2^H_11/2_ transition.^40^ The similarities and differences between the lanthanide coordination polymers are even more distinct in photoluminescence (PL) spectra.

**Fig. 6:**
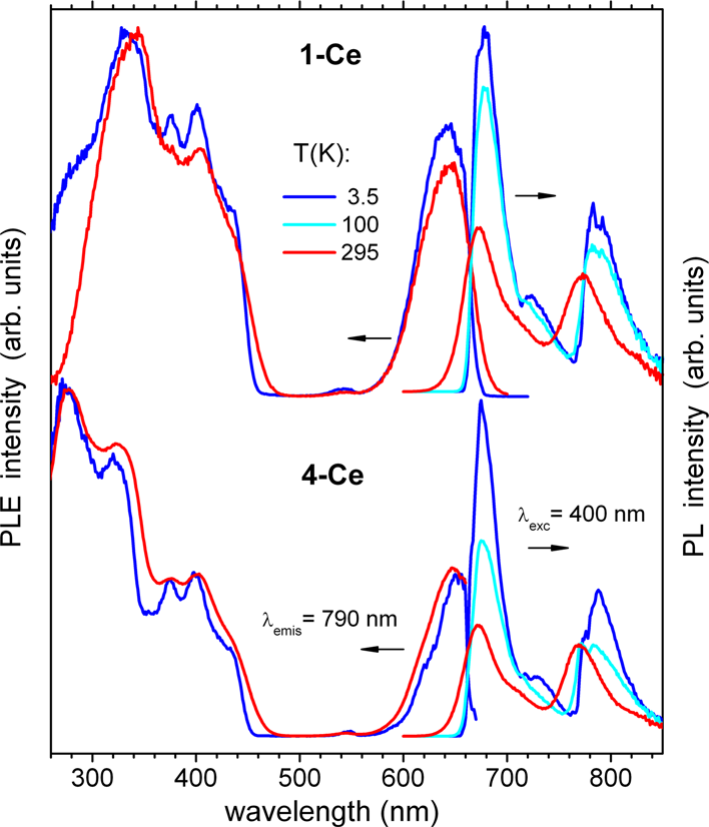
Photoluminescence excitation (PLE) and emission (PL) spectra of polycrystalline 1-Ce and 2-Ce at ambient and low temperatures. The spectra are recorded/excited at 790/400 nm, respectively.

**Fig. 7:**
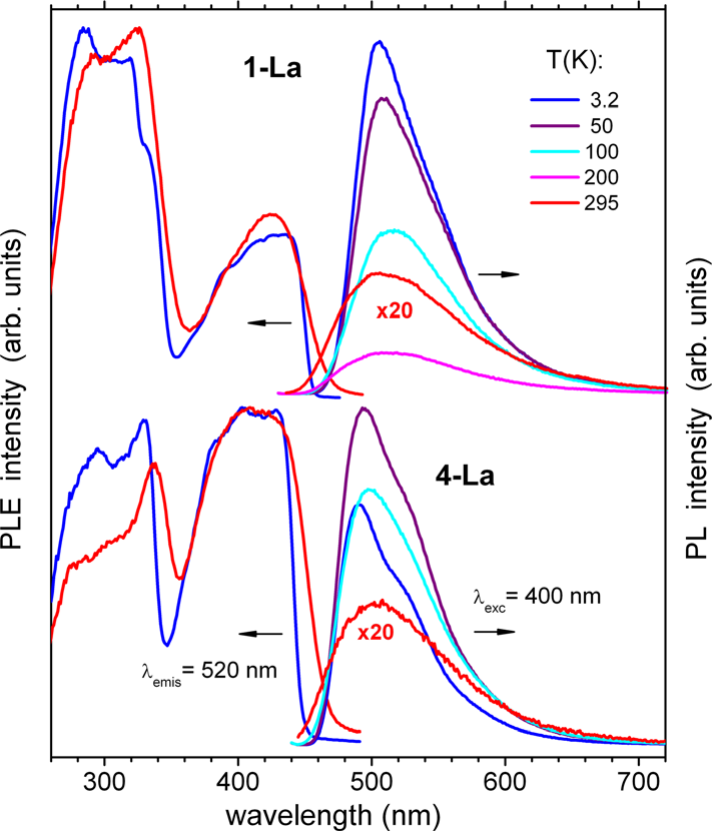
Photoluminescence excitation (PLE) and emission (PL) spectra of polycrystalline 1-La and 2-La at temperatures between 3.2 and 295 K. The spectra are recorded/excited at 520/400 nm, respectively.

As it can be expected, the closely related helix and zigzag 1,2-Ce structures show quite similar PL properties (Fig. 6). The excitation (PLE) spectra correspond well to the absorption, including both the ligand bands below 460 nm and Ce^III^-localized band at 650 nm. The red emission demonstrates a small Stokes shift and two components centered at 673 and 772 nm at *T* = 295 K (1-Ce), which shift to 676 and 788 nm at 3.5 K. The total quantum efficiency, *Φ*_PL_, was determined for 1,2-Ce in an integrating sphere at ambient temperature as 7.9% and 7.6% (*λ*_exc_ = 400 nm), respectively, and estimated from the temperature-dependent PL spectra as 12% and 13% at 3.5 K. The two components are assigned to ^2^D → ^2^F_5/2_ and ^2^D → ^2^F_7/2_ transitions of Ce^III^, respectively, with a characteristic energy splitting of about 1900 cm^−1^.^39^ The same PLE spectra and emission lifetimes (*τ* ≈ 50 ns nearly independent of temperature, Fig. S32†) indicate that they originate from a common excited state, supporting their assignment. The above PL parameters including two-band Ce^III^ -localized emission and short lifetimes of tens of nanoseconds are rather typical for cerium complexes. Unusual, however, is the spectral position of the emission, shifted into the deep red region. Its first component (^2^D → ^2^F_5/2_) has been typically observed between 350 and 550 nm.^39,41^ To the best of our knowledge, 1,2-Ce are thus the first examples of Ce^III^ compounds with ^2^D → ^2^F_5/2_ absorption and emission arising at 650 and 690 nm, *i.e.* far above 550 nm. We tentatively attribute such behavior to the quasi-linear arrangement of the ligands and lanthanide ions and, correspondingly, to unusual ligand field splitting. Another factor may be specific metal-ring interactions in the excited state as studied theoretically for Ce(Cot)_2_.^42^ A similar observation of unusually red-shifted emission bands was made for 2-Nd (see below). In 1,2-La, the lanthanum cations (in the xenon-like electronic configuration) can be considered as photophysically ‘inactive’ in comparison to other lanthanides. Nevertheless, 1,2-La demonstrate green luminescence which can only be attributed to Cot^TIPS^ ligands (Fig. 7). As expected, the emission and excitation spectra are rather similar for both structures and the PLE curves follow the absorption (Fig. 5). We note here that the literature reports on the PL of Cot-lanthanide complexes are scarce and do not reveal any common trends. For instance, no PL was detected in solid state from the sandwich complex of La with η^8^-Cot and η^9^-cyclononatetraenyl ligands.^43^ On the other hand, PL was also not detected from the homologous Ce^III^ compound. The latter gained, however, a Ce^III^-typical emission (with ^2^D → ^2^F_5/2_ transition at 500 nm) after replacement of cyclononatetraenyl by coordinated solvent (thf) molecules.^43^ The green emission of 1,2-La is moderate at ambient temperature with *Φ*_PL_ = 1.7% and 2.8% (*λ*_exc_ = 410 nm), respectively, but becomes the major relaxation channel at low temperatures, with *Φ*_PL_ ≈ 65 and 60% at 3.2 K. It is phosphorescence as indicated by lifetimes of a few hundreds of microseconds at low temperatures (Fig. S33†). The emission decay strongly accelerates at elevated temperatures, roughly correlating with the decrease of *Φ*_PL_ due to non-radiative electronic relaxation which becomes dominating. The PL lifetimes at ambient temperature, determined as average values from biexponential fits, drop to 0.75 μs and 0.65 μs for 1-La and 2-La, respectively. In addition to slight differences in emission maxima and moderate differences in the lifetimes and *Φ*_PL_, 2-La shows in comparison to 1-La a more distinct shoulder at ∼520 nm in the emission spectra below 50 K. This may be assigned to a vibronic feature with spacing of 1100–1400 cm^−1^, which likely relates to a ring stretching vibration of Cot^TIPS^ ligands.

In contrast to the above compounds, no appreciable PL was observed from 2-Nd, 2-Lu and 2, 3-Er. These lanthanides apparently function as very efficient electronic relaxation sinks in the polymeric structures. Namely, the characteristic near-infrared (NIR) emissive f–f transitions of Nd^III^ and Er^III^ were detected within 900–1200 nm and around 1535 nm, respectively, but of very low intensity. Accordingly, we applied a sensitive FTIR-PL technique and laser excitation at 375 nm (for details see the ESI†) to record the emission spectra presented in Fig. S34–S37.† Also with this setup, an efficient emission detection was only possible by using excitation below ∼480 nm (limited to discrete laser lines), *i.e.* into the ligand-related absorption bands (Fig. 5). 3-Er emits the characteristic peak at 1536 nm (^4^I_13/2_ → ^4^I_15/2_ transition) with a minor pattern of surrounding sublevel satellites. Its intrinsic bandwidth was estimated to be as small as 1.5/3 cm^−1^ at 3/295 K, indicating a very homogeneous environment of the Er ions in 3-Er. Interestingly, the satellite pattern is well-resolved at 295 K, but broadened at 3 K. A weak emission peak at 978 nm assigned to the ^4^I_11/2_ → ^4^I_15/2_ transition is observed at 3 K but disappears at ambient temperature (Fig. S36†). In difference to 3-Er, we found that the NIR emission of 2-Nd is significantly, ∼30 nm red-shifted relative to the typical spectra reported for Nd^III^-based metal–organic complexes and inorganic materials. The groups of peaks corresponding to the characteristic ^4^F_3/2_ → ^4^I_9/2_ and ^4^F_3/2_ → ^4^I_11/2_ transitions are observed in 2-Nd within ∼880–950 nm and 1080–1190 nm, respectively (Fig. S34†). For instance, the well-known NIR emission band of Nd^III^ typically found at about 1060 nm is shifted in 2-Nd to *ca.*1090 nm. Similar to 3-Er, the emission peaks are quite narrow at ambient temperature and further split into multiplet patterns (with ∼1–3 cm^−1^ sharp lines) by cooling the sample down to 3 K (Fig. S33†). We note, that decomposition of 2-Nd after ∼1 day exposure to air completely transforms the unusual NIR emission of 2-Nd into a spectrum which is typical for Nd^III^ complexes and inorganic materials with a relatively high degree of disorder, demonstrating broad emission bands at the ordinary spectral positions (Fig. S34†).^44–46^

### Magnetism

It is known that lanthanide ions show interesting magnetic behavior due to the intrinsic properties of their well-shielded 4f shell. Especially the latter half of the lanthanide series Tb–Yb is magnetically interesting as the strong spin–orbit coupling yields very high total angular momentum quantum numbers (*J*) and with that high magnetic moments. In single-molecule magnetism (SMM), Er(iii)-based compounds are among the most studied systems, as their exerted magnetic moment is among the highest next to Tb(iii), Dy(iii) and Ho(iii). Due to the half-integer spin of Er(iii) and Dy(iii) ions, they are bound to show an energetically degenerate ground state, following the Kramers theorem. This intrinsic bistability of the magnetic ground state results in slow relaxation of the magnetization when the ions are placed in a suitable ligand environment. Following Rinehart and Long,^37^ the 4f electron distribution of Ln(iii) ions can be categorized into prolate (elongated along the *z*-axis, *e.g.* Er^3+^) and oblate (elongated within the *x-y*-plane, *e.g.* Dy^3+^).

The SMM properties of ions characterized as prolate benefit from being placed in an equatorial ligand field, while axial ligand field arrangements are suitable for oblate ions. Previous studies have revealed that the ligand field provided by COT-type ligands is highly equatorial.^12,19,47^ Under these assumptions we have prepared and magnetically analyzed the Er compounds 2-Er and 3-Er of the previously discussed molecular coordination polymers. The paramagnetic analogues of Ce, Pr and Nd have also been investigated, although their oblate nature is non-beneficial in terms of single-molecule magnetism. The temperature dependent magnetic susceptibility has been tested by cooling the samples in an applied field of 1000 Oe (Fig. S38†). The χ_M_T values at 300 K of the two Er-compounds 2-Er and 3-Er are found at 8.26 and 10.45 cm^3^ K mol^−1^, respectively. Both values are considerably lower than the theoretical value of 11.48 cm^3^ K mol^−1^ that would be expected for a single uncoupled Er^3+^ ion (*J* = 15/2, *g*_J_ = 6/5). We attribute this deviation to the sample preparation, where smearing of the sample across the holder cannot be avoided, causing an error in the mass of the sample. The observed magnetization values at high magnetic fields are in line with the room temperature susceptibilities, confirming the validity of our measurement, *vide infra*. By cooling the samples to 9 K the susceptibilities gradually decrease to 6.49 and 9.21 cm^3^ K mol^−1^, respectively, which is explained by depopulation of the Stark sublevels. Below 9 K, however, the susceptibility abruptly drops to 2.36 and 3.57 cm^3^ K mol^−1^, measured at 2 K respectively, which is likely a consequence of magnetic blocking and antiferromagnetic coupling between the Er ions.

**Fig. 8:**
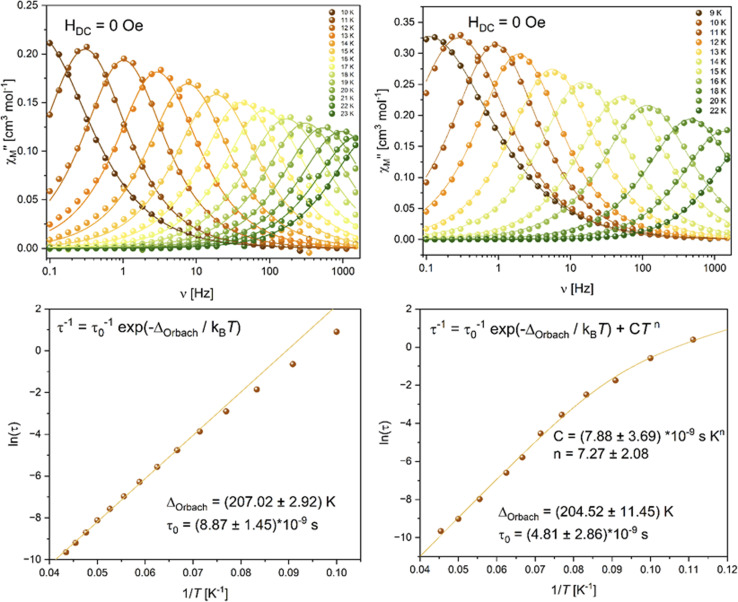
Top: frequency-dependent out-of-phase component of the magnetic susceptibility for 2-Er (left) and 3-Er (right), solid lines are the best fits to a generalized Debye model. Bottom: Arrhenius plots of the relaxation times and best fit using Orbach relaxation for 2-Er (left) and a combination of Orbach and Raman relaxation for 3-Er (right).

Measurements of the magnetization *versus* the applied magnetic field reveal a sigmoidal increase in the low field region (Fig. S55 and S56†), which is a consequence of magnetic blocking and antiferromagnetic coupling, matching the strong drop observed in the *T*-dependent susceptibilities. The obtained magnetization values at 7 T are 3.40 and 4.08 *μ*_B_, for 2-Er and 3-Er respectively. For highly anisotropic Er(iii) ions, the magnetization is expected to reach 4.5 *μ*_B,_ therefore, the deviations observed match what we have also observed in the χ_M_T(*T*) data. Frequency dependent AC studies at low *T* without an external DC field reveal a maximum in the out-of-phase component for both compounds 2-Er and 3-Er (Fig. 8, top). The maximum is observed in a temperature range between approximately 9 K and 22 K shifting from a frequency of 0.1 Hz towards our highest measurable frequency of 1500 Hz for both compounds. It is notable that the signal at 10 K shows a higher absolute intensity then at 9 K for 3-Er, although commonly a gradual decrease of the signal intensity is observed with increasing temperature. This observation can be explained with the antiferromagnetic coupling nicely matching our observation in the *T*-dependent DC data. Simultaneous fitting of the in-phase and out-of-phase component to a generalized Debye model give us a deeper insight into the relaxation behavior of 2-Er and 3-Er. As it can be seen in the Arrhenius plot (Fig. 8 bottom), the relaxation observed can be described almost exclusively through Orbach relaxation, characterized by a straight line in ln(*τ*) *vs.* 1/*T*. Only towards the lowest temperatures of around 10 K a deviation from this behavior is observed due to Raman processes. This is further confirmed by investigating the obtained α parameters increasing from 8% at 12 K to 23% at 10 K for 2-Er (Table S15†) from 12% at 12 K to 33% at 9 K for 3-Er (Table S16†).

The absence of quantum tunneling of the magnetization (QTM) within the observed frequency window means that QTM is not playing a major role in the relaxation of the Er-based coordination polymers. We therefore fitted the relaxation times using:

1 *τ*^−1^ = *τ*_0_^−1^e^−*Δ*_Orbach_/*k*_B_*T*^ + *CT*^*n*^

where the first term represents the Orbach relaxation and the second the Raman process (Fig. 8, bottom). For 2-Er the influence of Raman relaxation within the observed temperature window is too low, yielding unreasonably high standard deviations for the Raman parameter, we therefore opted to fit the relaxation data of 2-Er using a linear fit, representing Orbach relaxation. The best fits yielded *τ*_0_ = (8.87 ± 1.45) × 10^−9^ s and an energy barrier to the spin reversal of 207 ± 3 K (144 ± 2 cm^−1^) for 2-Er and *τ*_0_ = (4.81 ± 2.86) × 10^−9^ s and a barrier of 205 ± 11 K (142 ± 8 cm^−1^) for 3-Er. The obtained effective energy barriers are very comparable to the energy barrier of 147 cm^−1^ reported by Meihaus and Long^19^ for an isolated [Er(COT)_2_]^−^. Chen *et al.* have summarized a list of Er-COT single-molecule magnets, revealing the crucial role of the Er-COT distance to the observed effective energy barriers.^47^ Our observed barrier heights of 207 and 205 K correlate nicely to the emerging trend, considering the Er-COT distances of 1.87 and 1.92 Å for 2-Er and 1.87 and 1.94 Å for 3-Er. Higher energy barrier up to more than 400 K are observed only for compounds with considerably shorter Er-COT distances down to around 1.67 Å. It seems that despite the polymeric character of our compounds, the magnetic relaxation of each Er ion occurs isolated from its neighbors at temperatures above 10 K. This observation again correlates nicely with the measured DC data, as we expect the interactions between the Er ions to be negligible above 10 K. The Raman parameters for 3-Er were found to be *C* = (7.88 ± 3.69) × 10^−9^ s K^−*n*^ and *n* = 7.3 ± 2.08. Recently there has been much discussion about the meaning of the Raman parameter *n* obtained for SMMs using eqn (1).^48^ For a Kramers ion (like Er^3+^) it is expected to be *n* = 9.^49,50^ Often times best fits yield a much lower value of *n*, having no real physical meaning. Although *n* = 7.3 is closer to the expected value than for many other reported compounds, we also performed a fit of the Arrhenius data using a second exponential term for the Raman relaxation (Fig. S50†). We obtained essentially similar values for the Orbach process with *Δ*_Orbach_ = 204 K and *τ*_0_ = 4.85 × 10^−9^ s, as expected, and Raman parameters *Δ*_Raman_ = 73 K and *τ*_R_ = 4.46 × 10^−4^ s.

Using the determined Orbach parameters, we have estimated the blocking temperatures of the Er compounds as the temperature at which *τ* = 100 s. We find *T*_B_ = 8.94 K for 2-Er and an almost similar value of 8.61 K for 3-Er. Hysteresis measurements have been performed at different temperatures and field sweep rates, showing open hysteresis loops at low *T* (Fig. S53–S58†). The highest temperature at which we measured open loops using a slow sweep of 20 Oe s^−1^ is 8 K for both compounds, with the loops fully closed at 10 K. At the faster sweep rate of 100 Oe s^−1^, a minimal opening is still observed at 10 K for 2-Er, while the loop measured for 3-Er is closed. The estimated blocking temperatures are in good agreement with the recorded hysteresis loops, while the small opening at 10 K and 100 Oe s^−1^ for 2-Er matches the slightly higher calculated *T*_B_.

**Fig. 9:**
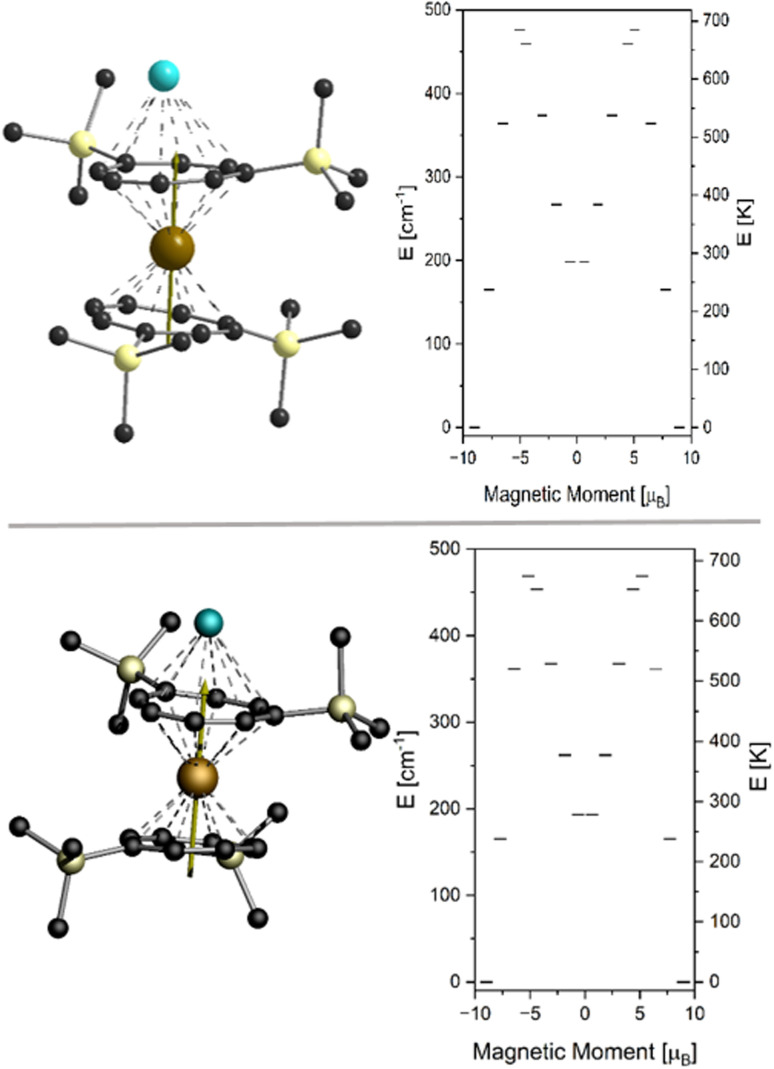
Orientation of the magnetic easy axis (left) and energy diagram of the *J* = 15/2 multiplet (right) obtained from CASSCF calculations for 2-Er (top) and 3-Er (bottom).

We have performed (11,7)-CASSCF calculations on a fictional Y-Er-Y fragment of the polymeric chain of 2-Er and 3-Er respectively. In order to keep the computational demand low enough for our system we have additionally replaced the TIPS groups with ordinary TMS groups, resulting in a fragment of the formula [K_2_ErY_2_(COT^TMS^)_6_]^−^. The two computations yield nearly identical outcomes, as anticipated. The magnetic doublet ground state is characterized by its g-tensor which was calculated as *g*_x_ < *g*_y_ < 10^−4^ and *g*_z_ = 17.94 for both 2-Er and 3-Er. These values correspond well to the *m*_J_ = 15/2 ground state without any considerable admixing of other states. The first excited Kramers doublet is an essentially pure *m*_J_ = 13/2 state found at 164.9 cm^−1^ and 165.3 cm^−1^ for 2-Er and 3-Er, respectively, while the second excited state is found at 198.27 cm^−1^ and 193.41 cm^−1^ with *m*_J_ = 1/2. In a situation like this, the magnetic relaxation typically proceeds through the second excited state as the charge distribution of the *m*_J_ = 1/2 differs significantly from that of the *m*_J_ = 15/2 and 13/2 states.^37,51^ However, comparison with the experimentally derived relaxation barrier suggests that relaxation through the first excited doublet state is the main process in the relaxation behavior of 2-Er and 3-Er (Fig. 9).

We have also accessed the magnetic properties of the two cerium analogues 1-Ce and 2-Ce and the Nd analogue 2-Nd. We have found all three compounds to show field-induced slow magnetic relaxation, evidenced by a frequency-dependent out-of-phase signal (Fig. S39–S41†). For 1-Ce the signal is observable up to approximately 5 K at an optimal DC field of 1400 Oe. At 2 K the maximum is centred at 600 Hz. Due to a very noisy signal resulting from the low moment of Ce(iii) and the maximum being found at the upper end of the frequency a window, reasonable interpretation of the *T*-dependent relaxation times was not possible. At the optimal DC field of 1000 Oe, 2-Ce reveals a maximum in the out-of-phase susceptibility centred around 200 Hz at 2 K. The maximum remains observable until approximately 8 K. Fits to a generalized Debye model and subsequent fitting of the relaxation times using eqn (1) gave *τ*_0_ = (4.80 ± 0.97) × 10^−4^ s, *Δ*_Orbach_ = 1.26 ± 0.57 K, *C* = (2.0 ± 0.4) × 10^−3^ s K^−*n*^ and *n* = 7.27 ± 1.05. Under an external field of 1800 Oe, 2-Nd shows slow relaxation of the magnetization with the maximum centred at around 0.2 Hz at 2 K. A full analysis of the AC data and final fitting using eqn (1) gave *τ*_0_ = (9.81 ± 1.20) × 10^−6^ s, *Δ*_Orbach_ = 38.20 ± 0.86 K, *C* = 0.38 ± 0.06 s K^−*n*^ and *n* = 2.17 ± 0.17.

## Conclusions

The introduction of the TIPS-substituted Cot ligand for the synthesis of Ln^III^-sandwich compounds [K{Ln^III^(ɳ^8^-Cot^TIPS^)_2_}]_*n*_ (Ln = La, Ce, Pr, Nd, Er, Lu) afforded three different and unique macromolecular structural motifs. Due to the increased solubility of the obtained target compounds, it was possible to crystallize them from the donor-free solvent toluene, thus resulting in a range of Lewis base free one-dimensional coordination polymers. In the process, the dependence of the solvent used for crystallization as well as the influence of the ionic radii on the shape of the obtained polymer chain could be systematically demonstrated. By crystallization from toluene the helical coordination polymer as well as the zigzag polymer were obtained as a mixture for the Pr-derivative. Thus, the ionic radius of the Pr^III^ cation was determined to be the inflection point for the preferential crystallization of the helical polymer toward the zigzag polymer. Furthermore, the photophysical properties of the different La, Ce, Nd and Er coordination polymers (1,2-La, 1,2-Ce, 2-Nd, 2, 3-Er, 2-Lu) were determined. Most remarkable, the La complexes show ligand centered green luminescence, while the Ce-complexes reveal red emission origin from d–f transitions. Furthermore, the magnetic properties of the Er compounds (2, 3-Er) were investigated. The Er-compounds show almost identical SMM behavior with open hysteresis up to 8 K and effective energy barriers of 207 and 205 K. This feature suggests that the magnetic relaxation occurs within the monomeric units rather than the polymeric chain.

## Data availability

All synthetic protocols, spectroscopic data, supplementary figures and tables, magnetic data, data from quantum chemical calculations and detailed crystallographic information can be found in the ESI.† Crystallographic data are available *via* the Cambridge Crystallographic Data Centre (CCDC): 2282875–2282882 and 2309731–2309732.

## Author contributions

Experimental work: AH, LM with support from CU. Magnetic measurements: SS, TTR. Photophysical measurements: SL. Project administration: PWR. Supervision: MR, MM, PWR. Writing – original draft: AH. Conceptualization: PR. Writing – review & editing: all authors.

## Conflicts of interest

There are no conflicts to declare.

## Supplementary Material

SC-015-D3SC03746K-s001

SC-015-D3SC03746K-s002
