# Supplementary material for: It's not just the size that matters: crystal engineering of lanthanide-based coordination polymers
Source: Chem Sci. 2023 Dec 20;15(4):1338–47. doi: 10.1039/d3sc03746k (PMC10806785; doi:10.1039/d3sc03746k)
Supplement: SC-015-D3SC03746K-s001 [file SC-015-D3SC03746K-s001.pdf]

Electronic Supplementary Material (ESI) for Chemical Science.
This journal is © The Royal Society of Chemistry 2023

Supporting Information

It's not just the size that matters: Crystal engineering of lanthanide-based coordination polymers

Adrian Hauser[1†], Luca Münzfeld[1†], Cedric Uhlmann[1], Sergei Lebedkin[2], Sören Schlittenhardt[2], Ting-Ting Ruan[5], Manfred M. Kappes[2,3], Mario Ruben[2,4,5] and Peter W. Roesky*[1]

[1] Institute of Inorganic Chemistry, Karlsruhe Institute of Technology (KIT), Engesserstraße 15, D-76131 Karlsruhe, Germany. E-mail: roesky@kit.edu

[2] Institute of Nanotechnology, Karlsruhe Institute of Technology (KIT), Hermann-von-Helmholtz-Platz 1, D-76344 Eggenstein-Leopoldshafen, Germany.

[3] Institute of Physical Chemistry, Karlsruhe Institute of Technology (KIT), Fritz-Haber-Weg 2, D-76131 Karlsruhe, Germany.

[4] Centre Européen de Science Quantique (CESQ); Institut de Science et d'Ingénierie Supramoléculaires (ISIS, UMR 7006), CNRS-Université de Strasbourg, 8 allée Gaspard Monge BP 70028 67083 Strasbourg Cedex, France

[5] Institute of Quantum Materials and Technologies (IQMT), Karlsruhe Institute of Technology, Hermann-von-Helmholtz-Platz 1, 76344 Eggenstein-Leopoldshafen, Germany.

† These authors contributed equally.

General Methods S3
Synthetic Part S4
Analytical data: S5
NMR Spectra S7
FT-Raman Spectra S15
X-ray Crystallographic Studies S18
PL/PLE Spectra S32
Magnetism S35
Ab initio calculations S50
References S51

General Methods

All manipulations of air-sensitive materials were performed under rigorous exclusion of oxygen and moisture in flame-dried Schlenk-type glassware either on a dual manifold Schlenk line, interfaced to a high vacuum pump (10^{-3} mbar), or in an argon-filled MBraun glove box. Hydrocarbon solvents were pre-dried using an MBraun solvent purification system (SPS-800), degassed and stored *in vacuo* over $LiAlH_4$. Tetrahydrofuran was additionally distilled under nitrogen over potassium before storage *in vacuo* over $LiAlH_4$. THF was dried over K and degassed by freeze-pump-thaw cycles. Elemental analyses were carried out with an Elementar Vario MICRO Cube. NMR spectra were recorded on Bruker spectrometers (Avance III 300 MHz, Avance 400 MHz, or Avance III 400 MHz). Chemical shifts are referenced internally using signals of the residual protio solvent (^{1}H) or the solvent (^{13}C{^{1}H}) and are reported relative to tetramethylsilane (^{1}H, ^{13}C, ^{29}Si). All NMR spectra were measured at 298 K. The multiplicity of the signals is indicated as s = singlet, d = doublet, sept. = septet, m = multiplet, and br = broad. Raman spectra were recorded in the region of 4000-20 cm^{-1} on a Bruker MultiRam spectrometer equipped with a Nd:YAG laser (λ = 1064 nm) and a germanium detector at a resolution of 2 cm^{-1}. The powdered crystalline sample materials were flame sealed in a glass tube. The laser energy was adjusted to values between 20 and 200 mW depending on the FID amplitude and laser focusing. In terms of their intensity, the signals were classified into different categories (vs = very strong, s = strong, m = medium, w = weak). [K_2(Cot^{TIPS})] (Cot^{TIPS} = 1,4-(^{i}Pr_3Si)$_3$$C_8H_6^{2-}$)[1,2] was synthesized according to literature procedure. All other chemicals were obtained from commercial sources and used without further purification.

Note: To ensure the best possible purity and reliability of all compounds, only crystalline material was isolated. Hence all yields and analytics refer to isolated crystalline samples, whereas yields are generally lower compared to bulk samples.

Photoluminescence (PL) measurements were performed with Horiba Jobin Yvon Fluorolog-322 and Fluorolog-QM spectrometers. The first instrument included a Hamamatsu NIR photomultiplier with an emission detection range up to 1400 nm. High-resolution emission spectra in the near-infrared range were acquired with a FTIR spectrometer (Bruker Vertex 80) equipped with a liquid nitrogen cooled germanium detector and different CW and quasi-CW lasers for photoexcitation. Polycrystalline samples were typically prepared as dispersions in a thin layer of perfluoroether oil between quartz plates and measured in an optical cryostat based on a Cryomech PT403 pulse tube cryocooler. All emission spectra were corrected for the wavelength-dependent response of the spectrometer and detector (in relative photon flux units). PL quantum yields of the above sample preparations were determined at ambient temperature in air using an integrating sphere which was installed into the sample chamber of the Fluorolog spectrometer. The uncertainty of these measurements was estimated to be ±10%. Emission decays of **1-Ce** and **2-Ce** were recorded using a TCSPC technique and a sub-ns pulsed laser diode at 403 nm. Relatively slow PL decays of **1-La** and **2-La** were generated with a ns-pulsed nitrogen laser at 337 nm and recorded with a fast digital oscilloscope connected to the detector (photomultiplier).

Absorbance spectra of polycrystalline compounds (the same sample preparations as for PL measurements) were recorded with an Agilent Cary 5000 spectrometer, using a 6-inch integrating sphere in transmission mode.

Synthetic Part

General synthetic procedure of [K{Ln^{III}(η^8-Cot^{TIPS})$_2$}]$_n$ (Ln = La, Ce, Pr, Nd, Er)

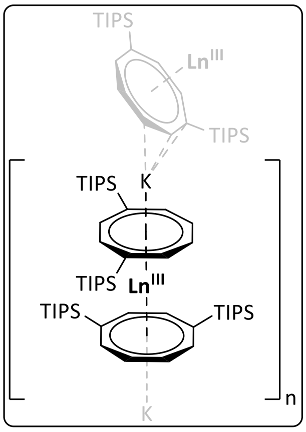

To a mixture of the corresponding lanthanide chloride (La: 124 mg $La^{III}Cl_3$, Ce: 125 mg $Ce^{III}Cl_3$, Pr: 126 mg $Pr^{III}Cl_3$, Nd: 127 mg $Nd^{III}Cl_3$, Er: 138 mg $Er^{III}Cl_3$, Lu: 142 mg $Lu^{III}Cl_3$, 0.506 mmol, 1.00 eq) and 500 mg [K_2(Cot^{TIPS})] (1.01 mmol, 2.00 eq) 25 mL THF was added at room temperature. The reaction mixture was stirred at room temperature for 12 h, resulting in a characteristic color (La: orange, Ce: green, Pr: yellow, Nd: bright green, Er: pink, Lu: orange) of the solution and the formation of a colorless precipitate was observed. After removing the solvent *in vacuo*, the oily residue was extracted with 25 mL of hot toluene (Lu: 20 mL of r.t. Et_2O) and filtered through a glass frit. The residue was extracted with toluene (Lu: Et_2O) until the filtrate remains colorless. Subsequently the filtrate was concentrated until the formation of a microcrystalline solid was visible. After storage at -30 °C for two days the mother liquor was decanted, and the crystals were dried *in vacuo*. All compounds could be isolated as microcrystalline solids (La: light yellow, Ce: green, Pr: yellow, Nd: yellow, Er: yellow- orange, Lu: yellow).

Recrystallization from toluene solution

To obtain single crystals suitable for SCXRD measurements, 100 mg of the isolated microcrystalline solids of [K{Ln^{III}(η^8-Cot^{TIPS})$_2$}]$_n$ were solved in 15 ml of hot toluene. Insoluble residues were filtered by using a PTFE syringe filter and the filtrate was allowed to slowly cool to room temperature. Thus, on the one hand, the 1D helical coordination polymers **1-Ln** (Ln = La, Ce, Pr) and the 1D zigzag coordination polymers **2-Ln** (Ln = Pr, Nd) could be obtained. For the corresponding Er compound, the trapezoidal polymer **3-Er** was isolated by this route.

Note: The two different types of coordination polymers **1-Pr** and **2-Pr** were isolated side by side by recrystallization of [K{Pr^{III}(η^8-Cot^{TIPS})$_2$}]$_n$ from a hot toluene solution. For Lu no single crystals could be obtained from toluene.

Recrystallization from Et_2O solution

In another crystallization attempt, the oily residue obtained after removal of THF was extracted in 15 ml of Et_2O. The solution was then filtered into a double ampoule, degassed, and flame sealed. Through slow evaporation of the solvent, crystals of the 1D zigzag coordination polymer **2-Ln** (Ln = La, Ce, Er, Lu) could be isolated. Similar to the helical coordination polymer **1-La**, the zigzag polymer **2-La** showed green emission, whereas both **1-Ce** and **2-Ce** showed bright red luminescence (see the main text).

Analytical data:

Since the analytical data for the different types of coordination polymers (helical **1-Ln** or zigzag **2-Ln** and **2-Ln**) are identical, they are listed together.

[La^{III}(η^{8}-Cot^{TIPS})$_2$K]$_n$ (1-La/2-La): Yield: 289 mg (0.286 mmol), 56%. **Anal. calcd**. (%) for [$C_{52}H_{96}KLaSi_4$] (1011.68 g/mol): C 61.74, H 9.57; **found** (%): C 61.47, H 9.37. **^{1}H NMR** (400.3 MHz, THF-d_8): δ [ppm] = 6.28-6.25 (m, 4 H, C*H*-Cot), 6.18 (s, 4 H, C*H*-Cot), 6.09-6.06 (m, 4 H, C*H*-Cot), 1.51 (sept, $^{3}J_{HH}$ = 7.3 Hz, 12 H, Si(C*H*(CH$_3$)$_2$)$_3$), 1.19, 1.16 (two resonances d, $^{3}J_{HH}$ = 7.4 Hz, 72 H, Si(CH(C*H*$_3$)$_2$)$_3$). **^{13}C{^{1}H} NMR** (100.67 MHz, THF-d_8): δ [ppm] = 104.7 (*C*-Cot), 103.8 (*C*-Cot), 101.2 (*C*-Cot), 97.6 (*C*-Cot), 20.5 (two resonances, Si(CH(*C*H$_3$)$_2$)$_3$), 13.6 (Si(*C*H(CH$_3$)$_2$)$_3$). **^{29}Si{^{1}H} NMR** (79.52 MHz, THF-d_8): δ [ppm] = 4.5 (*Si*(CH(CH$_3$)$_2$)$_3$). **Raman:** $\tilde{v}$ [cm^{-1}] = 3055 (w), 2921 (m), 2865 (vs), 2757 (w), 2714 (w), 1488 (m), 1470 (m), 1383 (w), 1295(w), 1236 (w), 1159 (w), 1072 (w), 1005 (w), 970 (w), 932 (w), 884 (w), 785 (m), 753 (w), 667 (m), 638 (w), 572 (w), 521 (w), 484 (w), 442 (w), 360 (w), 222 (m).

[Ce^{III}(η^{8}-Cot^{TIPS})$_2$K]$_n$ (1-Ce/2-Ce): Yield: 260 mg (0.256 mmol), 51%. **Anal. calcd**. (%) for [$C_{52}H_{96}KCeSi_4$] (1012.89 g/mol): C 61.66, H 9.55; **found** (%): C 61.48, H 9.28. **^{1}H NMR** (400.3 MHz, THF-d_8): δ [ppm] = 2.88 (br, 12 H, Si(C*H*(CH$_3$)$_2$)$_3$), 2.30, 2.13 (two resonances, br, 72 H, Si(CH(C*H*$_3$)$_2$)$_3$). **^{13}C{^{1}H} NMR** (100.67 MHz, THF-d_8): δ [ppm] = 21.4 (two resonances, Si(CH(*C*H$_3$)$_2$)$_3$), 15.6 (Si(*C*H(CH$_3$)$_2$)$_3$). **^{29}Si{^{1}H} NMR** (79.52 MHz, THF-d_8): δ [ppm] = -4.1 (*Si*(CH(CH$_3$)$_2$)$_3$). **Raman:** $\tilde{v}$ [cm^{-1}] = 3046 (w), 2944 (m), 2921 (m), 2865 (vs), 2711 (w), 2132 (w), 1489 (w), 1471 (w), 1447 (w), 1364 (w), 1293 (w), 1235 (w), 1158 (w), 1072 (w), 970 (w), 933 (w), 882 (w), 752 (w), 666 (w), 636 (w), 571 (w), 520 (w), 490 (w), 445 (w), 262 (w), 233 (w), 207 (w).

[Pr^{III}(η^{8}-Cot^{TIPS})$_2$K]$_n$ (1-Pr/2-Pr): Yield: 236 mg (0.232 mmol), 46%. **Anal. calcd**. (%) for [$C_{52}H_{96}KPrSi_4$] (1013.69 g/mol): C 61.61, H 9.55; **found** (%): C 61.69, H 9.23. **^{1}H NMR** (400.3 MHz, THF-d_8): δ [ppm] = -1.24, -1.85 (two resonances, br, 72 H, Si(CH(C*H*$_3$)$_2$)$_3$), -2.05 (br, 12 H, Si(C*H*(CH$_3$)$_2$)$_3$). **^{13}C{^{1}H} NMR** (100.67 MHz, THF-d_8): δ [ppm] = 18.9, 18.1 (two resonances, Si(CH(*C*H$_3$)$_2$)$_3$), 17.2 (Si(*C*H(CH$_3$)$_2$)$_3$). **^{29}Si{^{1}H} NMR** (79.52 MHz, THF-d_8): δ [ppm] = -29.3 (*Si*(CH(CH$_3$)$_2$)$_3$). **Raman:** $\tilde{v}$ [cm^{-1}] = 3043 (w), 2945 (w), 2921 (w), 2864 (s), 2713 (w), 1488 (w), 1472 (w), 1447 (w), 1390 (w), 1365 (w), 1294 (w), 1234 (w), 1159 (w), 1067 (w), 1004 (w), 970 (w), 932 (w), 882 (w), 756 (m), 666 (w), 639 (w), 574 (w), 520 (w), 493 (w), 443 (w), 360 (w), 251 (w), 209 (w), 170 (w).

[Nd^{III}(η^8-Cot^{TIPS})$_2$K]$_n$ (2-Nd): Yield: 278 mg (0.272 mmol), 54%. **Anal. calcd**. (%) for [$C_{52}H_{96}KNdSi_4$] (1017.02 g/mol): C 61.41, H 9.51; **found** (%): C 61.81, H 8.97. **^{1}H NMR** (400.3 MHz, THF-d_8): δ [ppm] = 1.23 (br, 12 H, Si(C*H*(CH$_3$)$_2$)$_3$), 0.88 (br, 72 H, Si(CH(C*H*$_3$)$_2$)$_3$). **^{13}C{^{1}H} NMR** (100.67 MHz, THF-d_8): δ [ppm] = 20.9, 20.5 (Si(CH(*C*H$_3$)$_2$)$_3$), 18.9 (Si(*C*H(CH$_3$)$_2$)$_3$). **^{29}Si{^{1}H} NMR** (79.52 MHz, THF-d_8): δ [ppm] = -45.5 (*Si*(CH(CH$_3$)$_2$)$_3$). **Raman:** $\tilde{v}$ [cm^{-1}] = 3004 (w), 2944 (m), 2923 (m), 2865 (s), 2714 (w), 1623 (w), 1598 (w), 1471 (w), 1448 (w), 1385 (w), 1293 (w), 1234 (w), 1207 (w), 1159 (w), 1073 (w), 970 (w), 953 (w), 882 (m), 785 (w), 755 (w), 641 (w), 565 (w), 521 (w), 428 (w), 369 (w), 275 (w), 208 (w).

[Er^{III}(η^8-Cot^{TIPS})$_2$K]$_n$ (2-Er/3-Er): Yield: 322 mg (0.310 mmol), 60%. **Anal. calcd**. (%) for [$C_{52}H_{96}KErSi_4$] (1040.04 g/mol): C 60.05, H 9.30; **found** (%): C 60.09, H 8.86. **Raman:** $\tilde{v}$ [cm^{-1}] = 3053 (w), 2945 (m), 2923 (m), 2893 (m), 2865 (vs), 2752 (w), 2711 (w), 1486 (m), 1471 (m), 1448 (w), 1381 (w), 1364 (w), 1294 (w), 1236 (w), 1158 (w), 1073 (w), 1004 (w), 970 (w), 935 (w), 883 (m), 811 (w), 785 (w), 762 (w), 749 (m), 670 (w), 637 (w), 597 (w), 561 (w), 520 (w), 438 (w), 358 (w), 266 (w), 202 (w).

[Lu^{III}(η^8-Cot^{TIPS})$_2$K]$_n$ (2-Lu): Yield: 120 mg (0.286 mmol), 56%. **Anal. calcd**. (%) for [$C_{52}H_{96}KLuSi_4$] (1047.75 g/mol): C 59.61, H 9.24; **found** (%): C 61.47, H 9.37. **^{1}H NMR** (400.3 MHz, THF-d_8): δ [ppm] = 6.23-6.20 (m, 4 H, C*H*-Cot), 6.10 (s, 4 H, C*H*-Cot), 5.81-5.78 (m, 4 H, C*H*-Cot), 1.53 (sept, $^3J_{HH}$ = 7.3 Hz, 12 H, Si(C*H*(CH$_3$)$_2$)$_3$), 1.17, 1.13 (two resonances d, $^3J_{HH}$ = 7.6 Hz, 72 H, Si(CH(C*H*$_3$)$_2$)$_3$). **^{13}C{^{1}H} NMR** (100.67 MHz, THF-d_8): δ [ppm] = 100.5 (*C*-Cot), 97.6 (*C*-Cot), 95.0 (*C*-Cot), 93.4 (*C*-Cot), 20.5 (two resonances, Si(CH(*C*H$_3$)$_2$)$_3$), 13.7 (Si(*C*H(CH$_3$)$_2$)$_3$). **^{29}Si{^{1}H} NMR** (79.52 MHz, THF-d_8): δ [ppm] = 5.0 (*Si*(CH(CH$_3$)$_2$)$_3$). **FT-IR:** $\tilde{v}$ [cm^{-1}] = 2941 (vs), 2889 (m), 2862 (vs), 1462 (w), 1383 (vw), 1364 (vw), 1248 (vw), 1206 (vw), 1071 (vw), 1035 (w), 1013 (w), 990 (vw), 959 (vw), 933 (w), 920 (vw), 881 (m), 733 (w), 660 (w), 634 (m), 572 (w), 519 (vw).

Note: Due to the paramagnetic properties, no resonances for ring protons or ring carbon atoms of the eight membered ring were detected neither in the ^{1}H NMR spectra nor in the ^{13}C{^{1}H} NMR spectra for any of the compounds except **1-La/2-La**. For compounds **2-Er/3-Er** no meaningful NMR spectra were obtained.

NMR Spectra

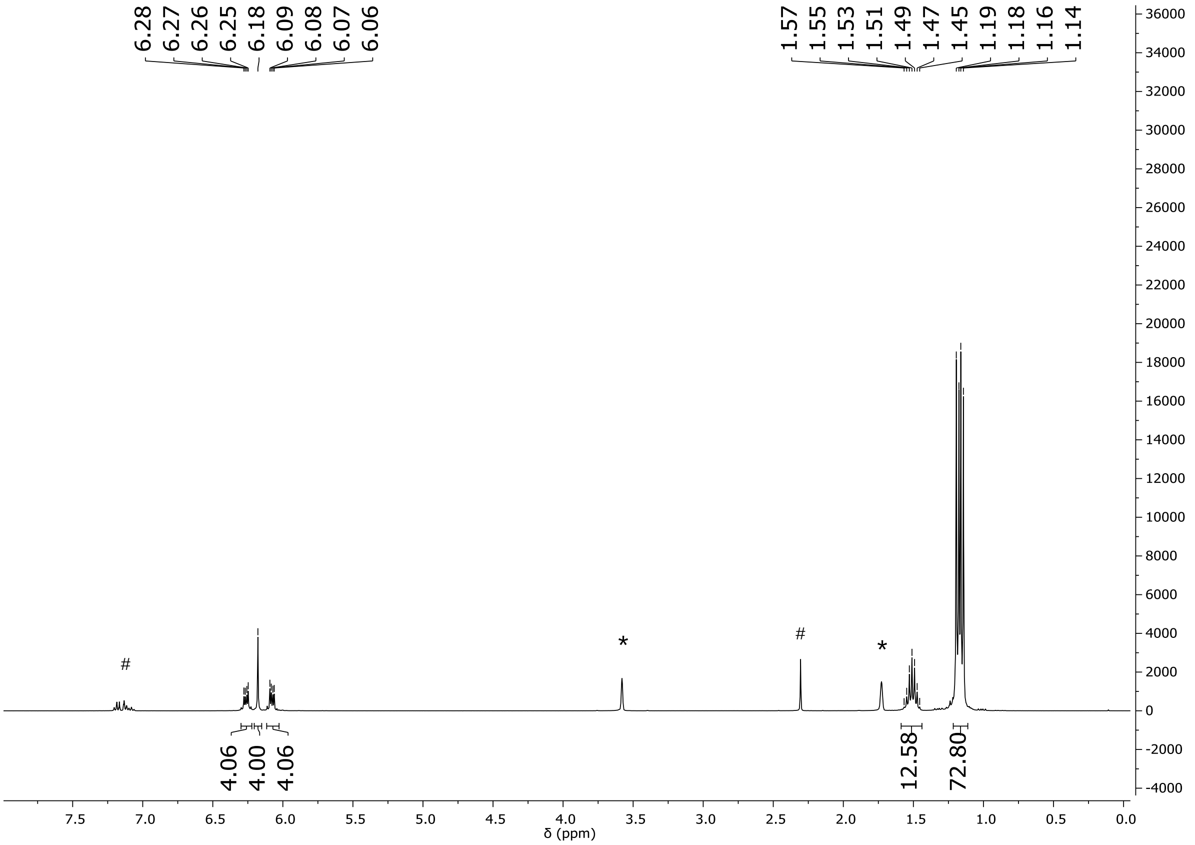

Figure S 1: ^{1}H NMR spectrum of $[La^{III}(\eta^{8}\text{-}Cot^{TIPS})_2K]_n$ (**1-La/2-La**) in THF-d_8 at 25 °C. *, residual protio solvent signal. #, toluene.

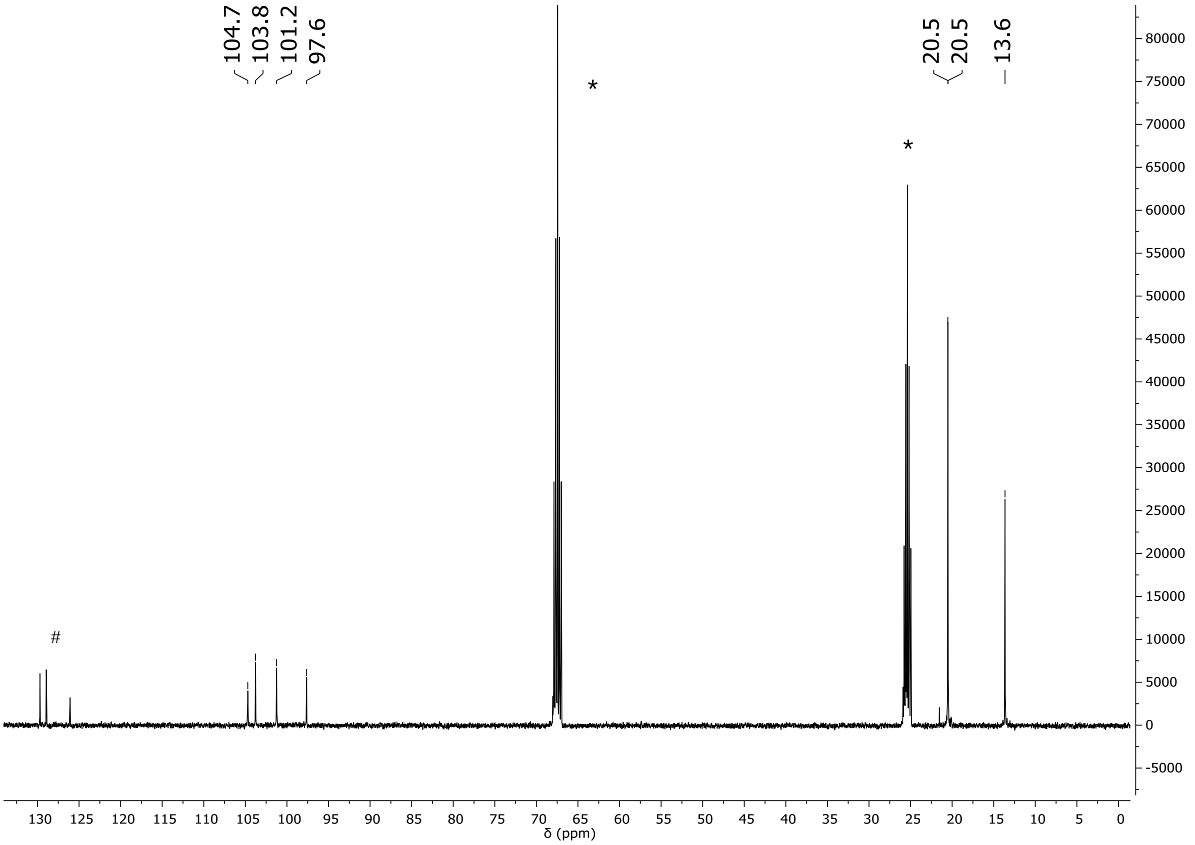

Figure S 2: $^{13}C\{^{1}H\}$ NMR spectrum of $[La^{III}(\eta^{8}\text{-}Cot^{TIPS})_2K]_n$ (**1-La/2-La**) in THF-d_8 at 25 °C. *, THF-d_8 signal. #, toluene.

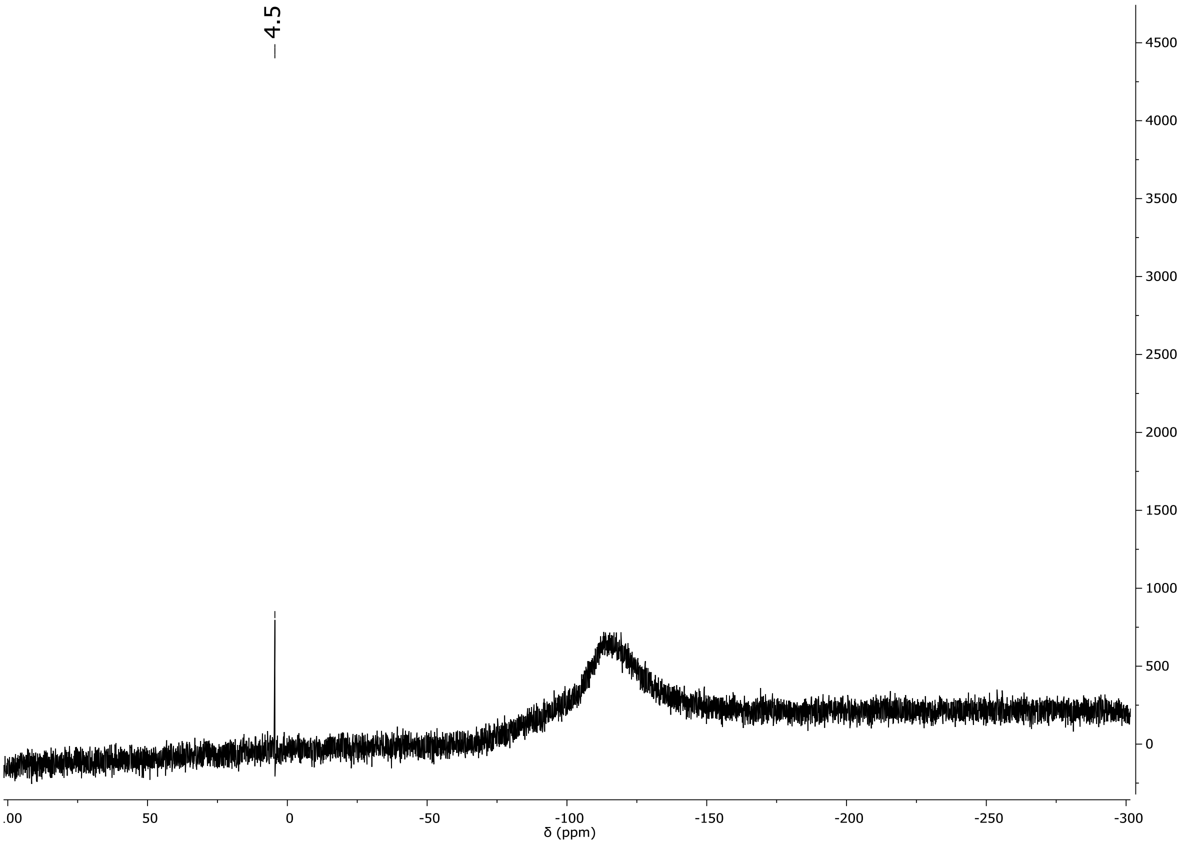

Figure S 3: $^{29}Si\{^{1}H\}$ NMR spectrum of $[La^{III}(\eta^8\text{-Cot}^{TIPS})_2K]_n$ (**1-La/2-La**) in THF-d_8 at 25 °C.

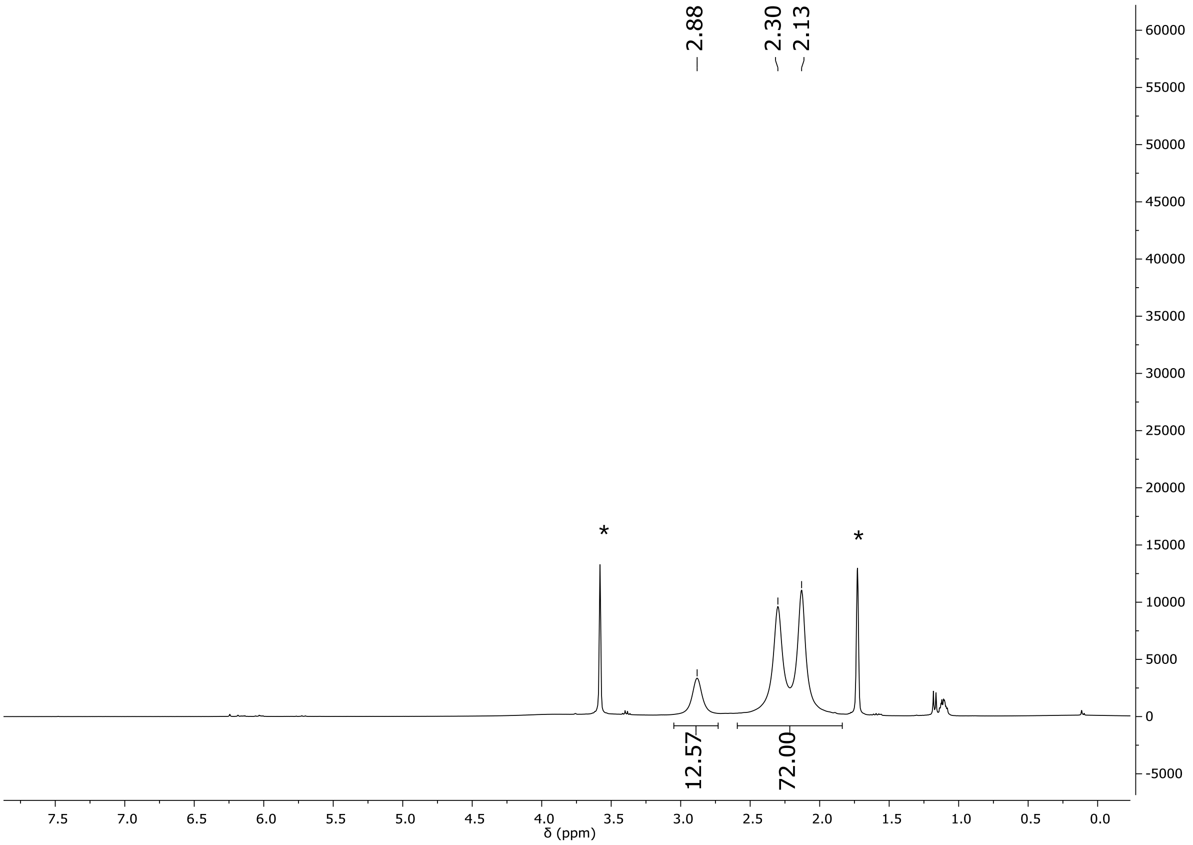

Figure S 4: ^{1}H NMR spectrum of $[Ce^{III}(\eta^8\text{-Cot}^{TIPS})_2K]_n$ (**1-Ce/2-Ce**) in THF-d_8 at 25 °C. *, residual protio solvent signal.

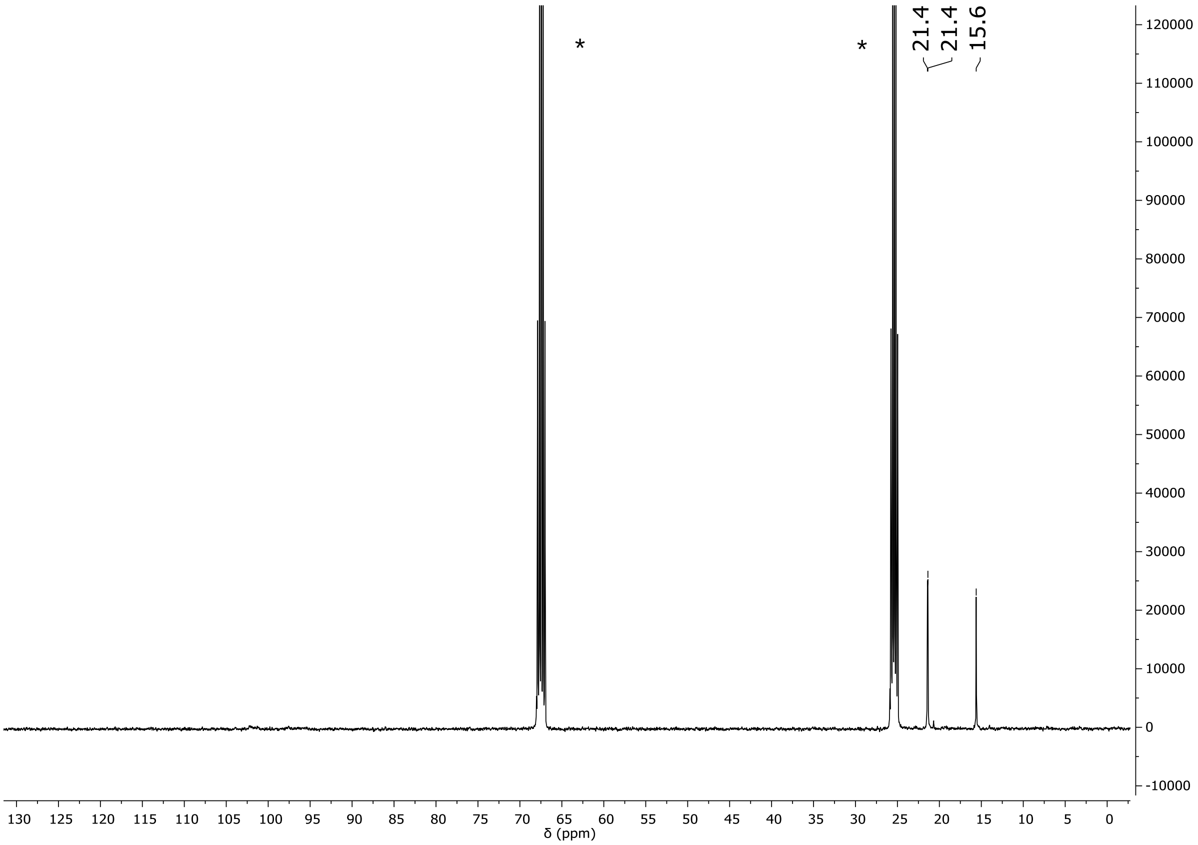

Figure S 5: $^{13}C\{^{1}H\}$ NMR spectrum of $[Ce^{III}(\eta^{8}\text{-}Cot^{TIPS})_2K]_n$ (**1-Ce/2-Ce**) in THF-d_8 at 25 °C. *, THF-d_8 signal.

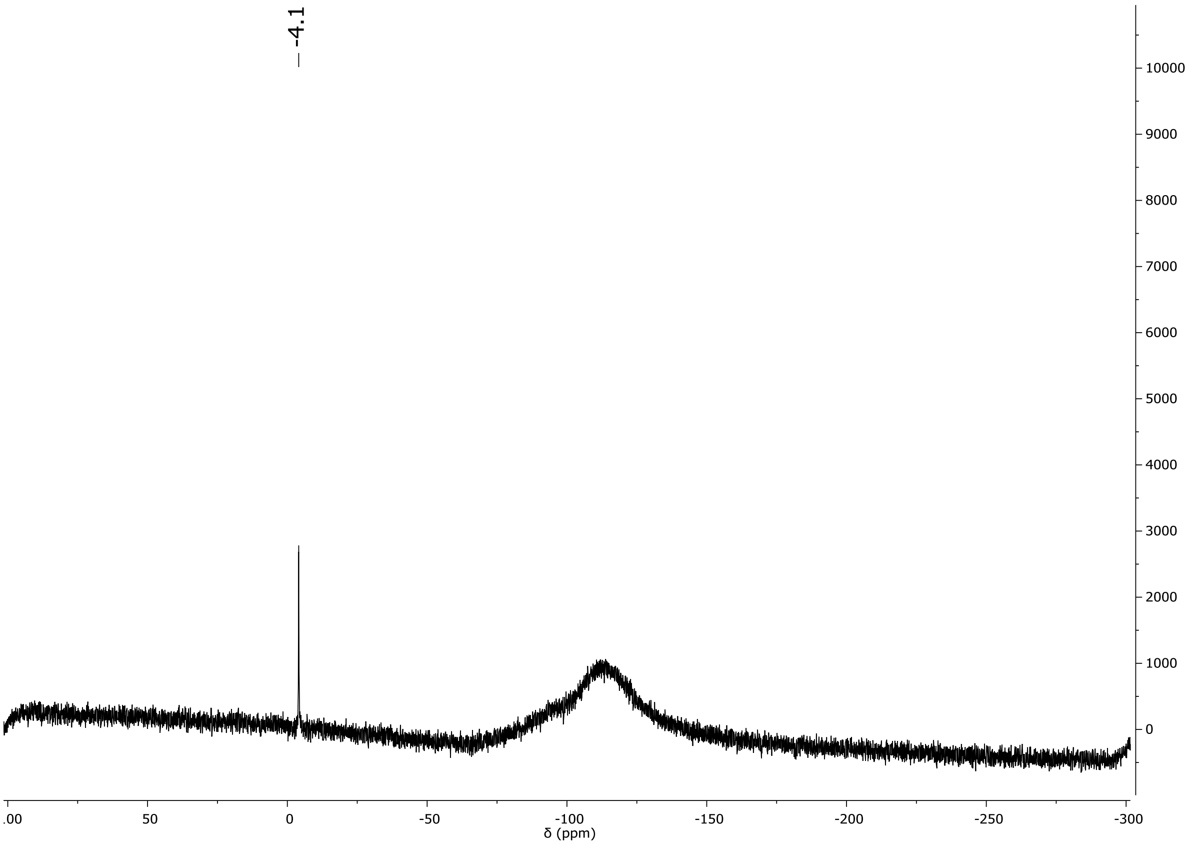

Figure S 6: $^{29}Si\{^{1}H\}$ NMR spectrum of $[Ce^{III}(\eta^{8}\text{-}Cot^{TIPS})_2K]_n$ (**1-Ce/2-Ce**) in THF-d_8 at 25 °C.

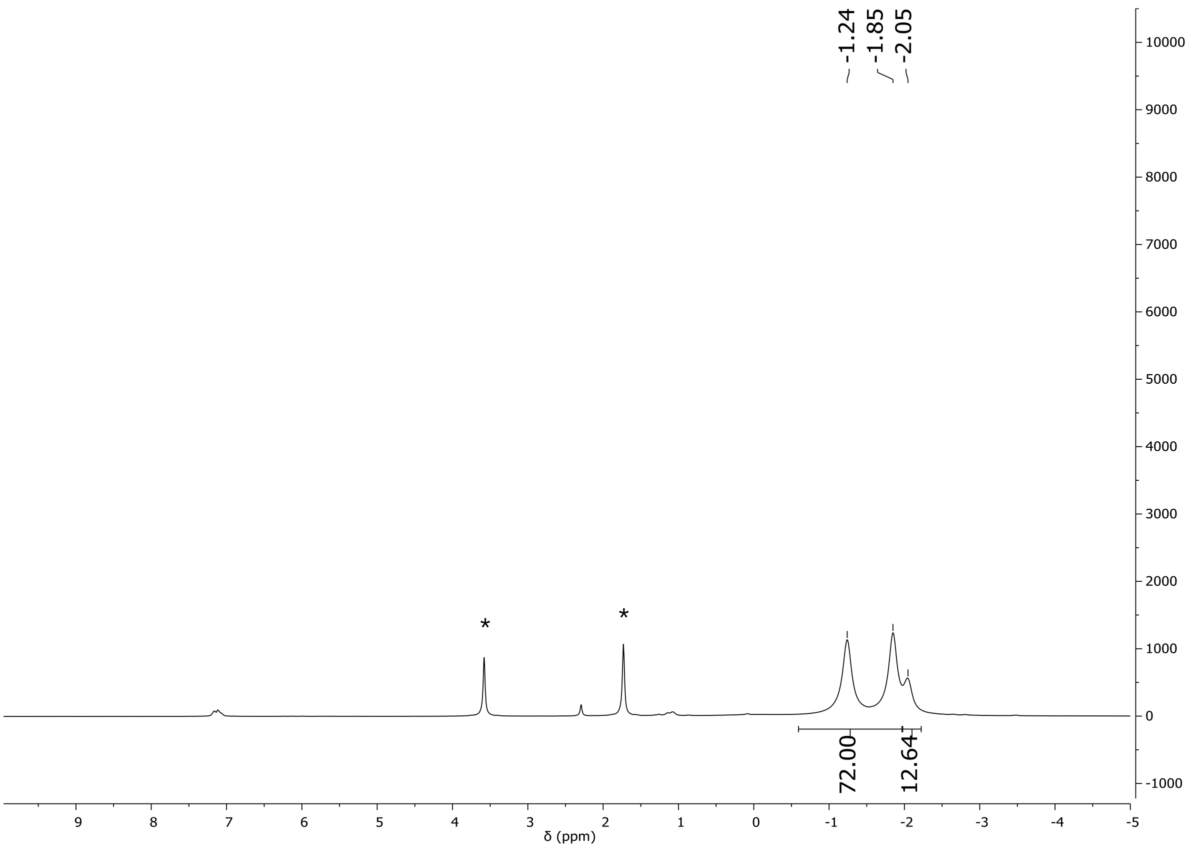

Figure S 7: ^{1}H NMR spectrum of $[Pr^{III}(\eta^8\text{-Cot}^{TIPS})_2K]_n$ (**1-Pr/2-Pr**) in THF-d_8 at 25 °C. *, residual protio solvent signal.

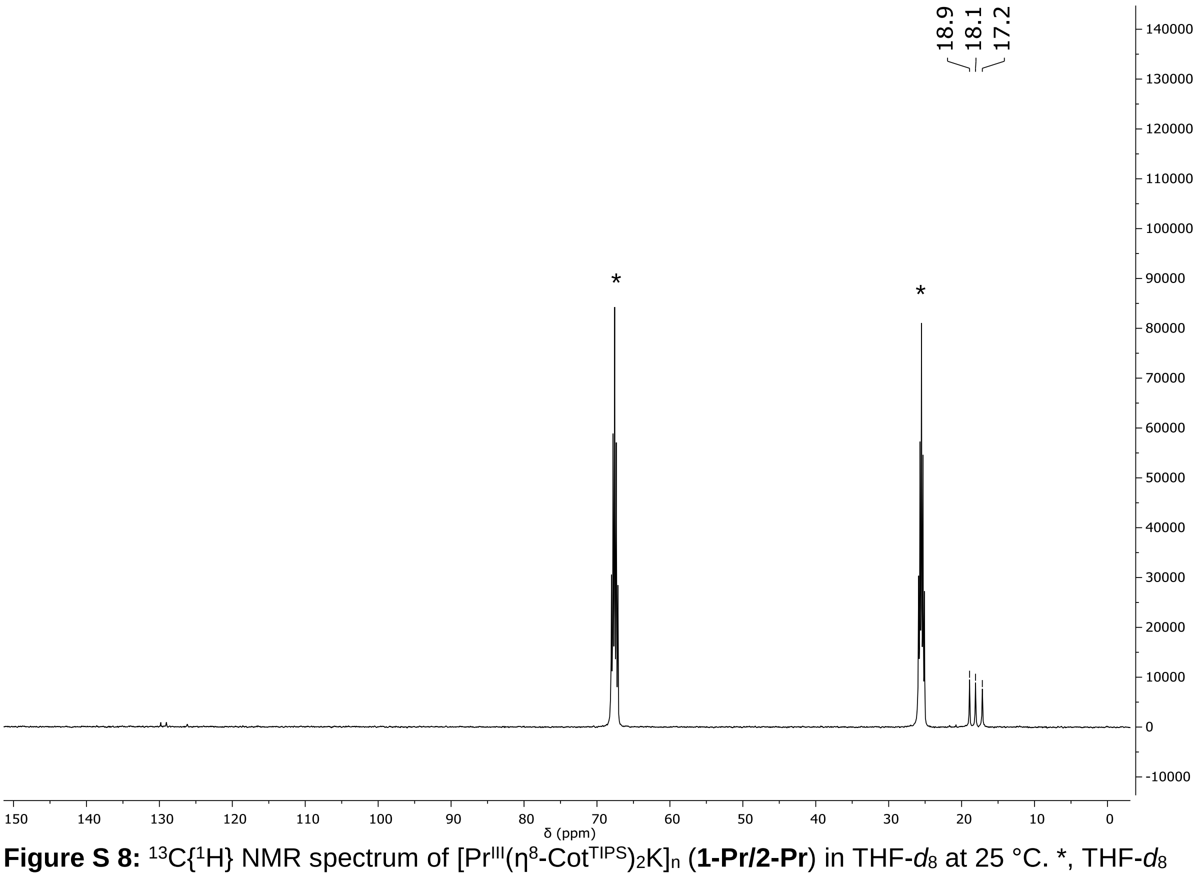

Figure S 8: $^{13}C\{^{1}H\}$ NMR spectrum of $[Pr^{III}(\eta^8\text{-Cot}^{TIPS})_2K]_n$ (**1-Pr/2-Pr**) in THF-d_8 at 25 °C. *, THF-d_8 signal.

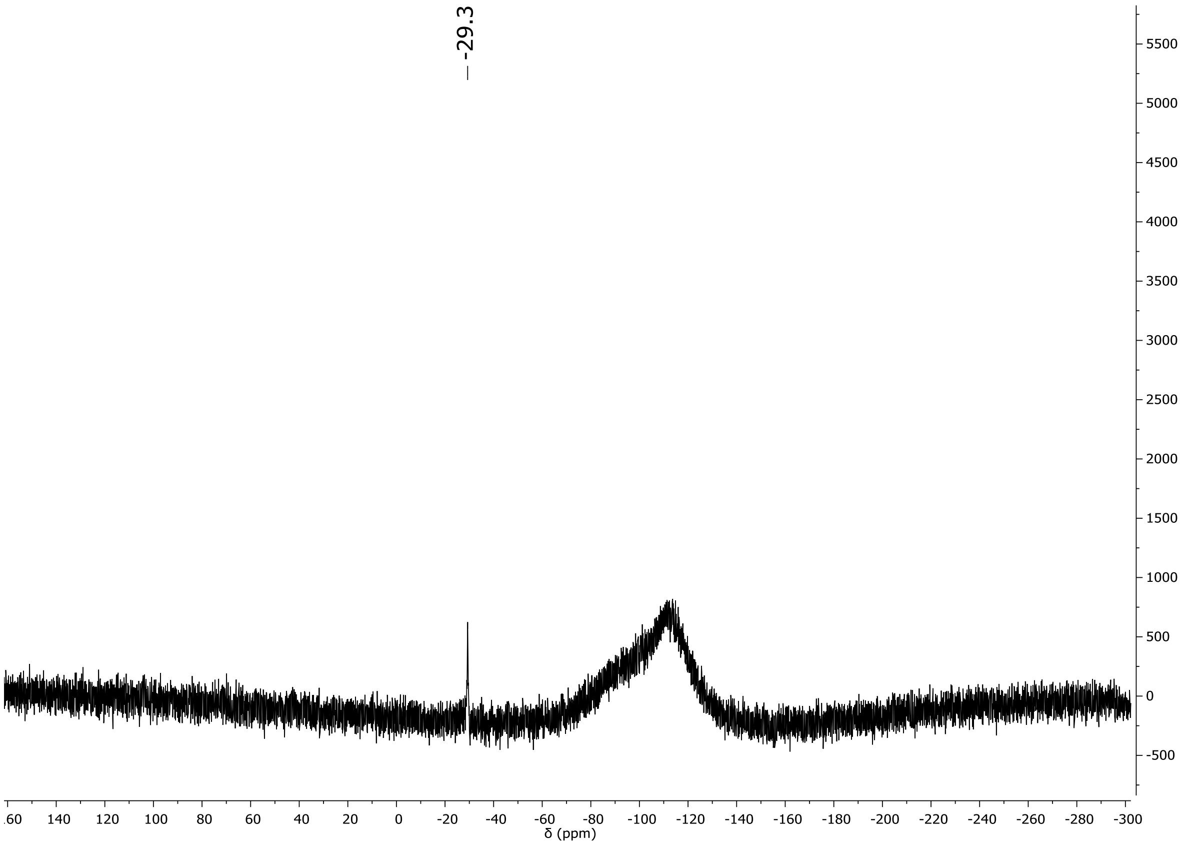

Figure S 9: $^{29}Si\{^{1}H\}$ NMR spectrum of $[Pr^{III}(\eta^8\text{-}Cot^{TIPS})_2K]_n$ (**1-Pr/2-Pr**) in THF-d_8 at 25 °C.

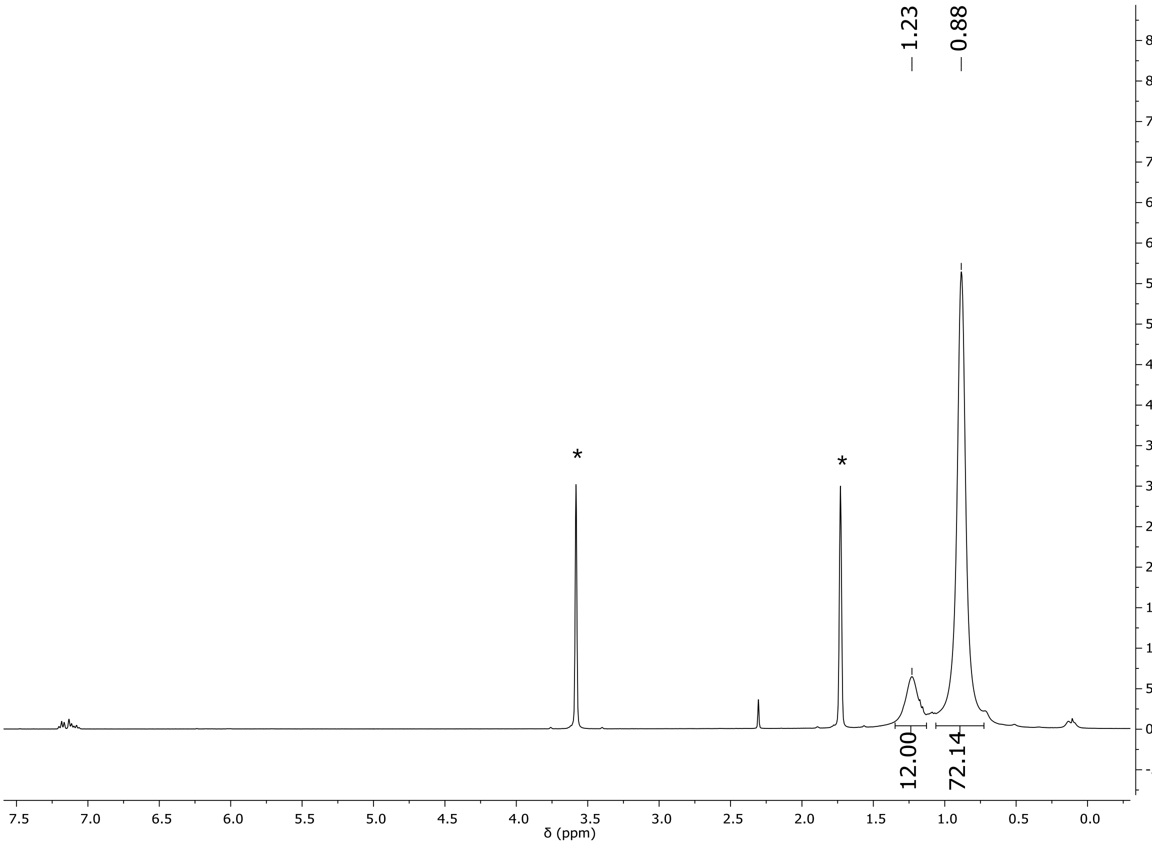

Figure S 10: ^{1}H NMR spectrum of $[Nd^{III}(\eta^8\text{-}Cot^{TIPS})_2K]_n$ (**2-Nd**) in THF-d_8 at 25 °C. *, residual protio solvent signal.

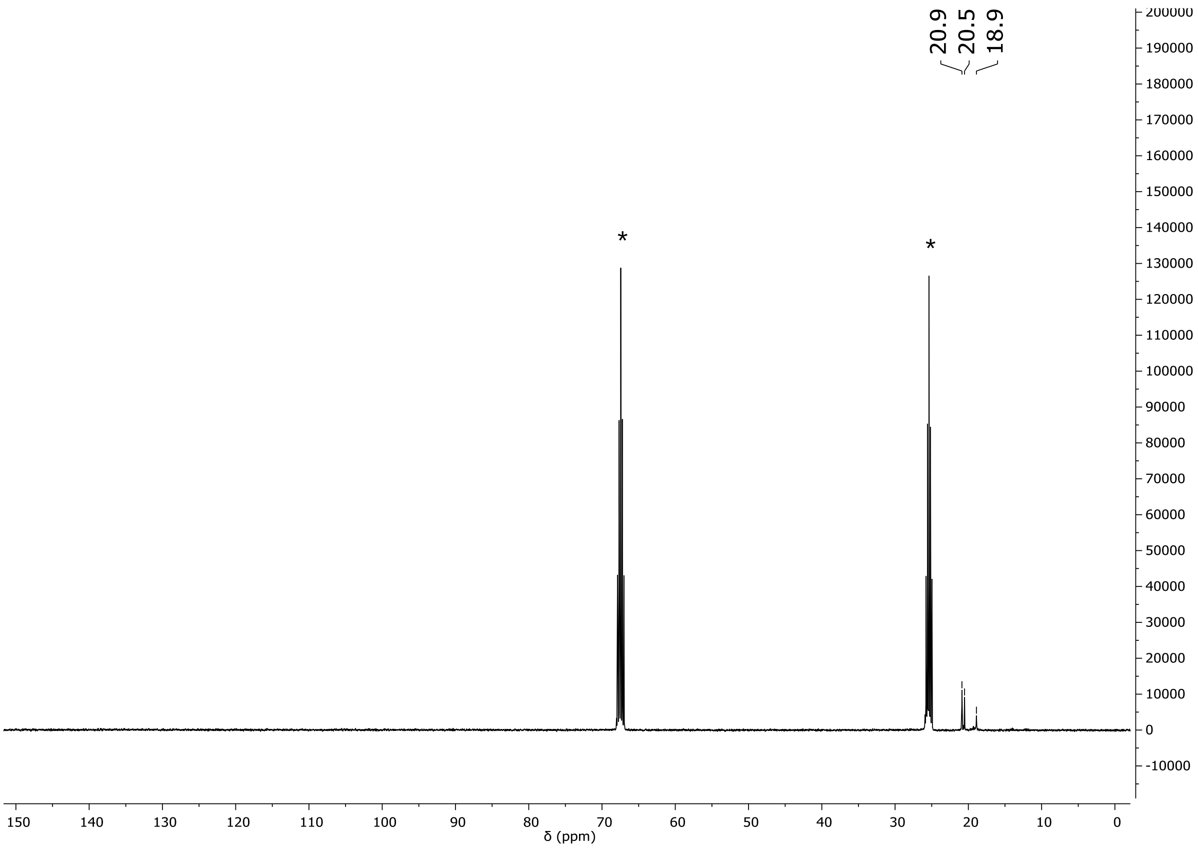

Figure S 11: $^{13}C\{^{1}H\}$ NMR spectrum of $[Nd^{III}(\eta^{8}\text{-}Cot^{TIPS})_{2}K]_{n}$ (**2-Nd**) in THF-d_8 at 25 °C. *, THF-d_8 signal.

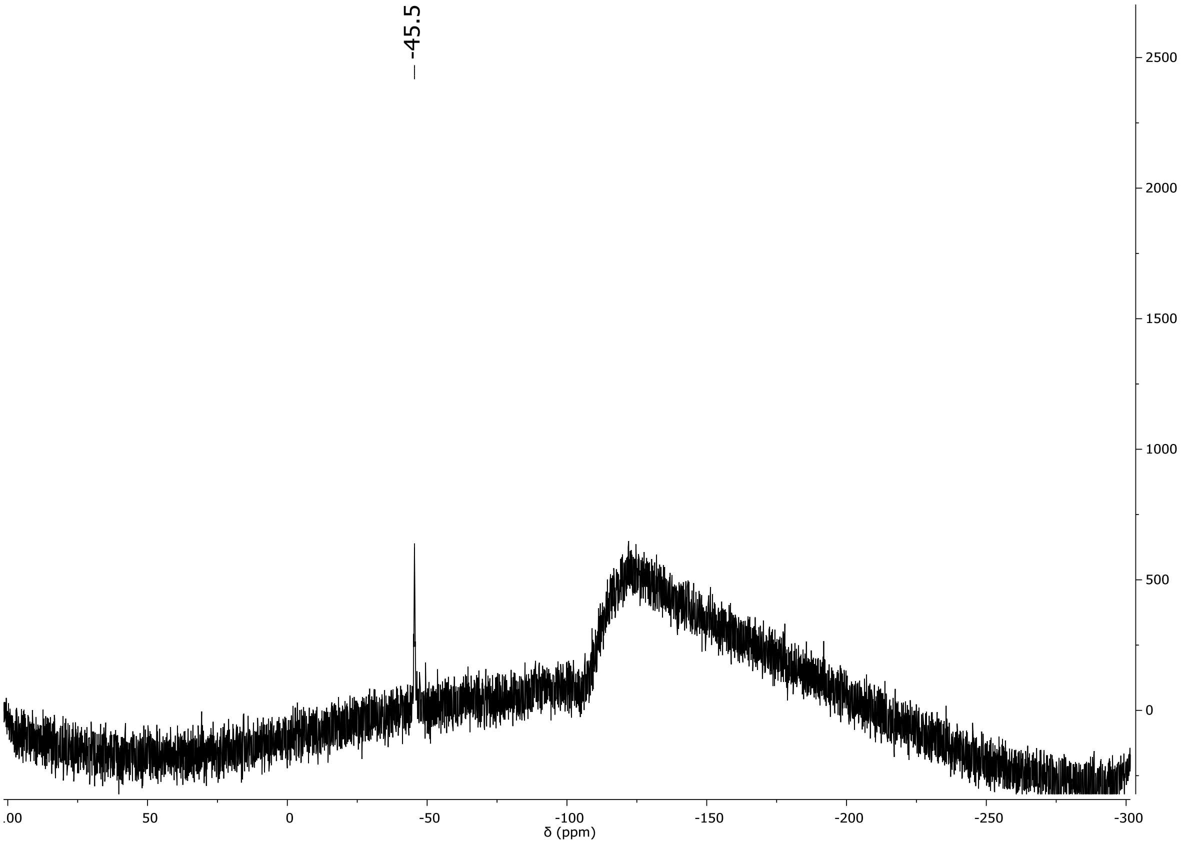

Figure S 12: $^{29}Si\{^{1}H\}$ NMR spectrum of $[Nd^{III}(\eta^{8}\text{-}Cot^{TIPS})_{2}K]_{n}$ (**2-Nd**) in THF-d_8 at 25 °C.

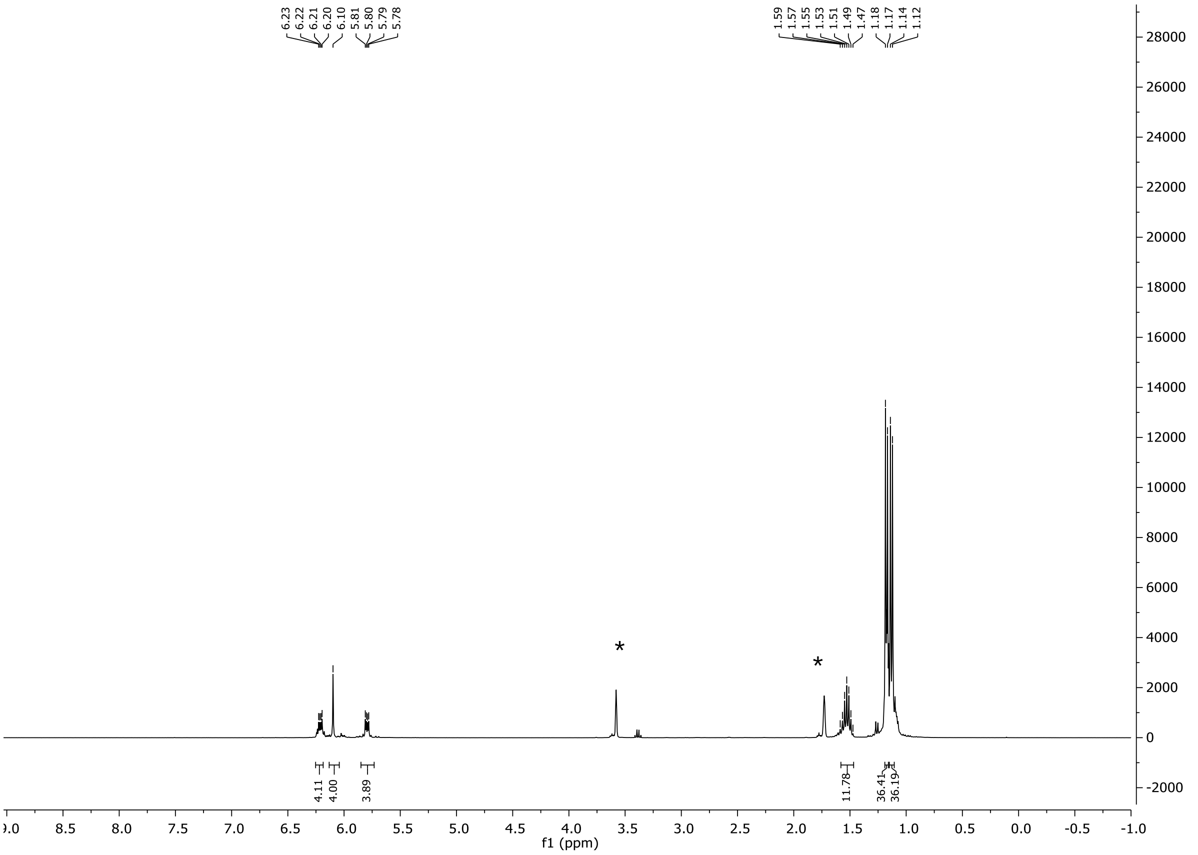

Figure S 13: ^{1}H NMR spectrum of $[Lu^{III}(\eta^8\text{-Cot}^{TIPS})_2K]_n$ (**2-Lu**) in THF-d_8 at 25 °C. *, residual protio solvent signal.

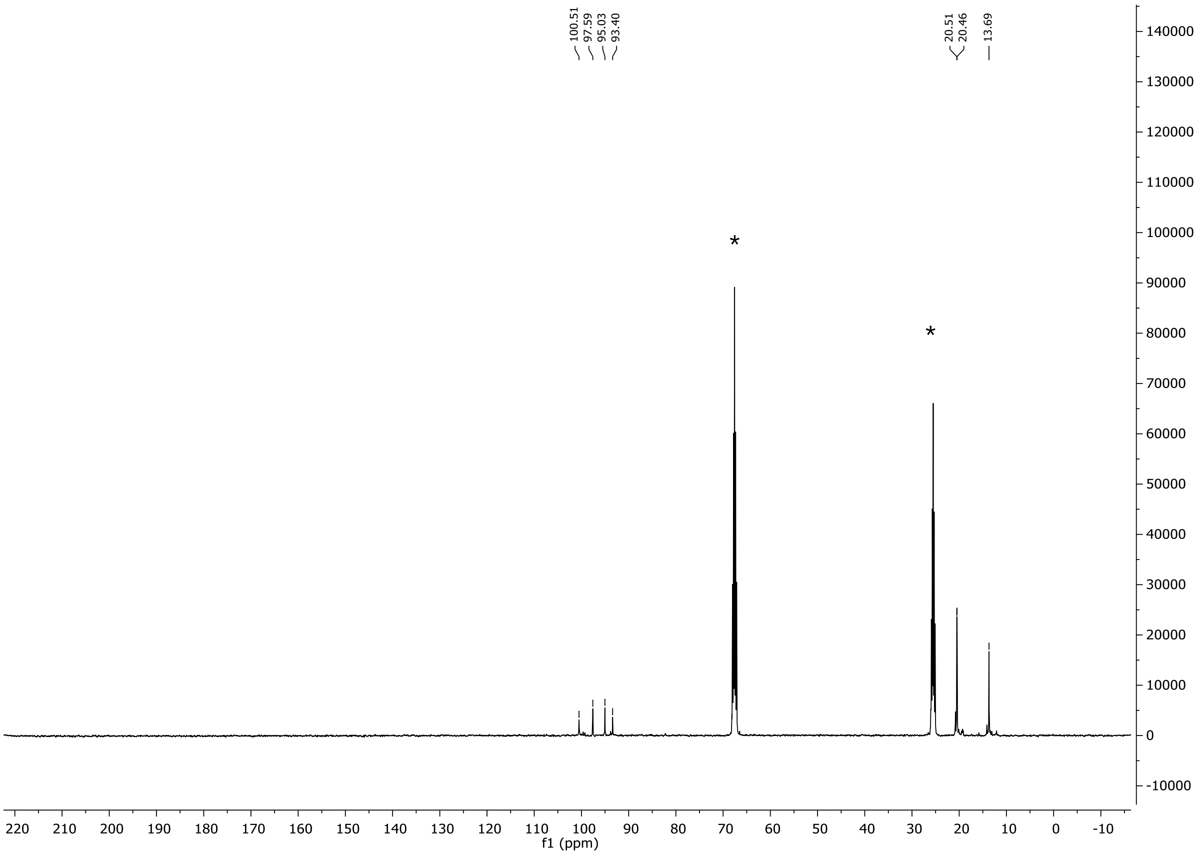

Figure S 14: $^{13}C\{^{1}H\}$ NMR spectrum of $[Lu^{III}(\eta^8\text{-Cot}^{TIPS})_2K]_n$ (**2-Lu**) in THF-d_8 at 25 °C. *, THF-d_8 signal.

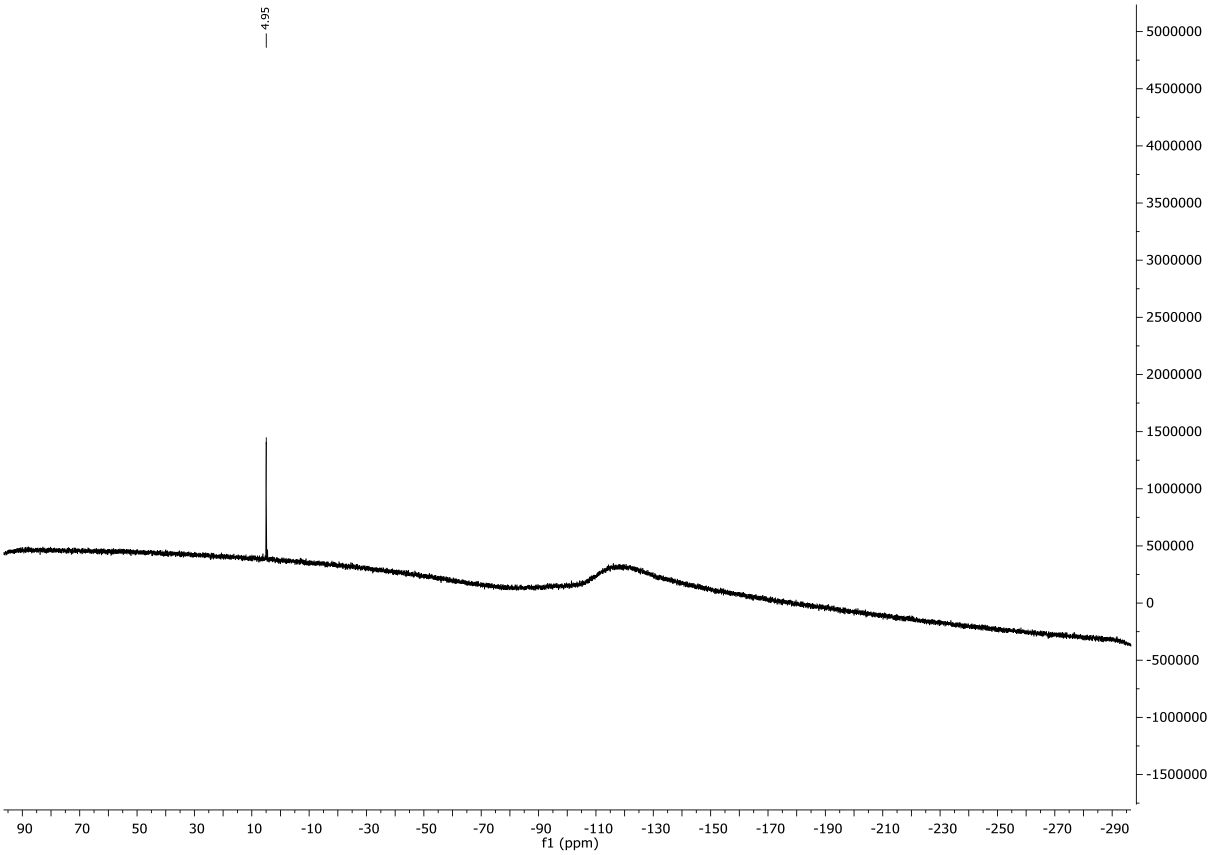

Figure S 15: $^{29}Si\{^1H\}$ NMR spectrum of $[Lu^{III}(\eta^8\text{-}Cot^{TIPS})_2K]_n$ (**2-Lu**) in THF-d_8 at 25 °C.

FT-Raman Spectra

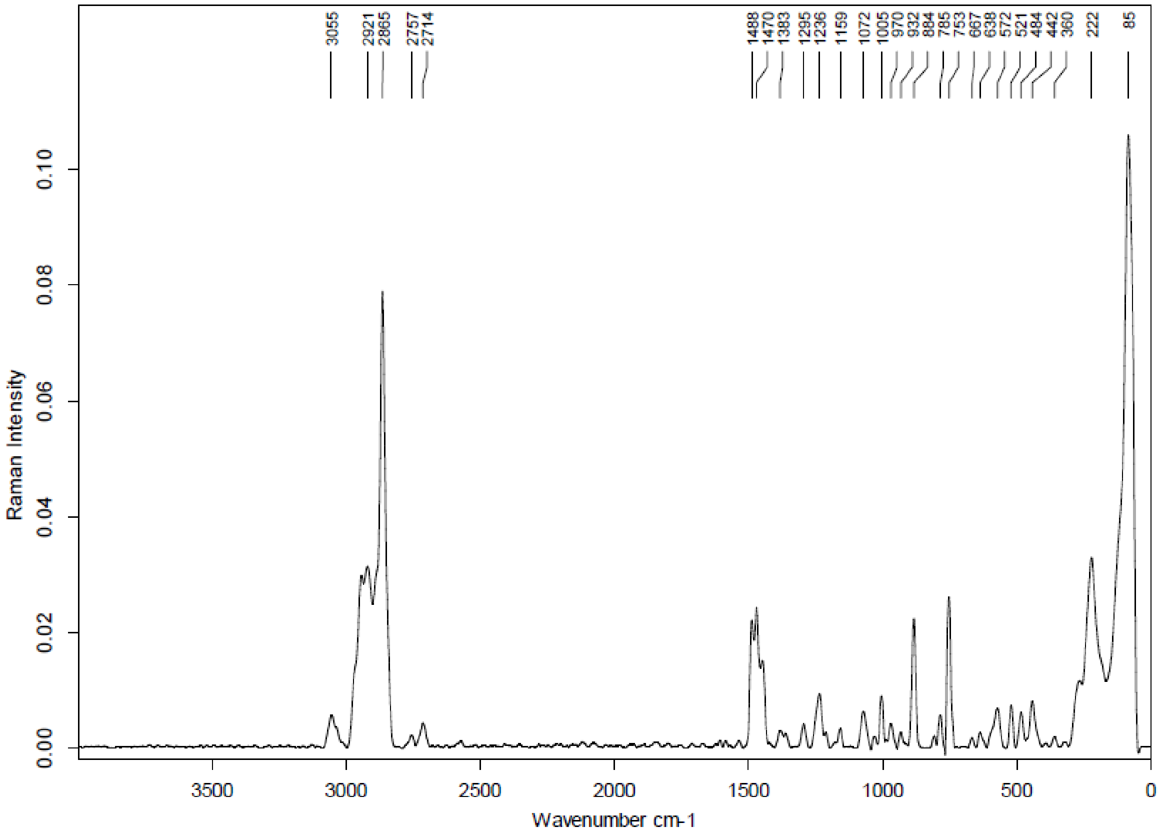

Figure S 16. FT-Raman spectrum of $[La^{III}(\eta^8\text{-}Cot^{TIPS})_2K]_n$ (**1-La/2-La**).

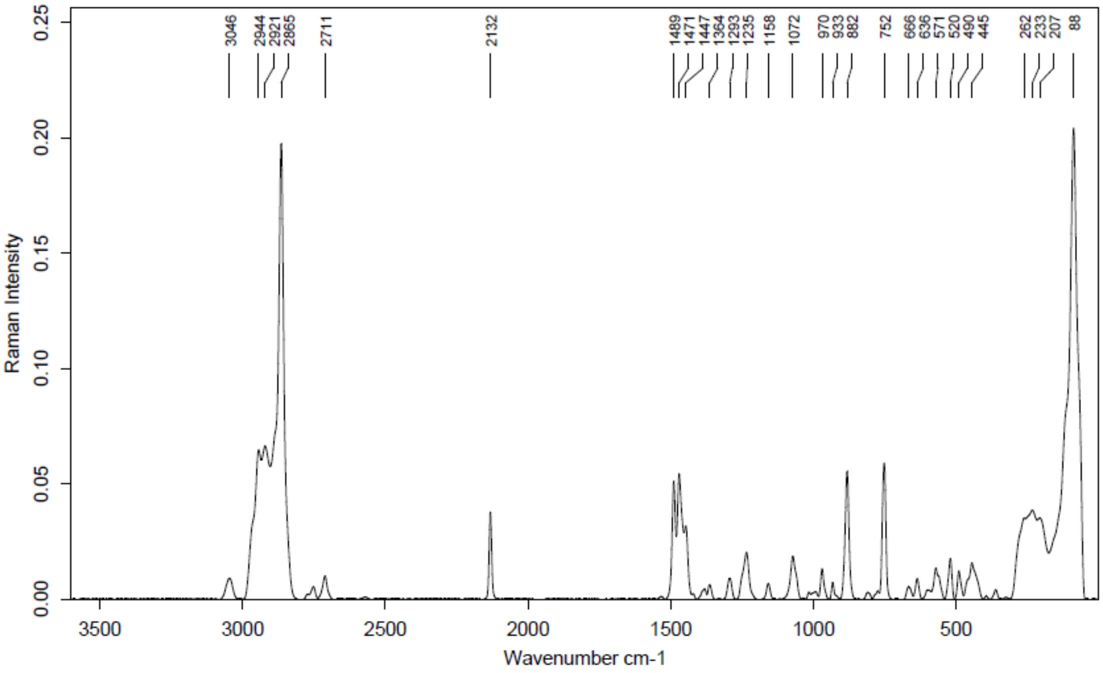

Figure S 17: FT-Raman spectrum of $[Ce^{III}(\eta^8\text{-}Cot^{TIPS})_2K]_n$ (**1-Ce/2-Ce**).

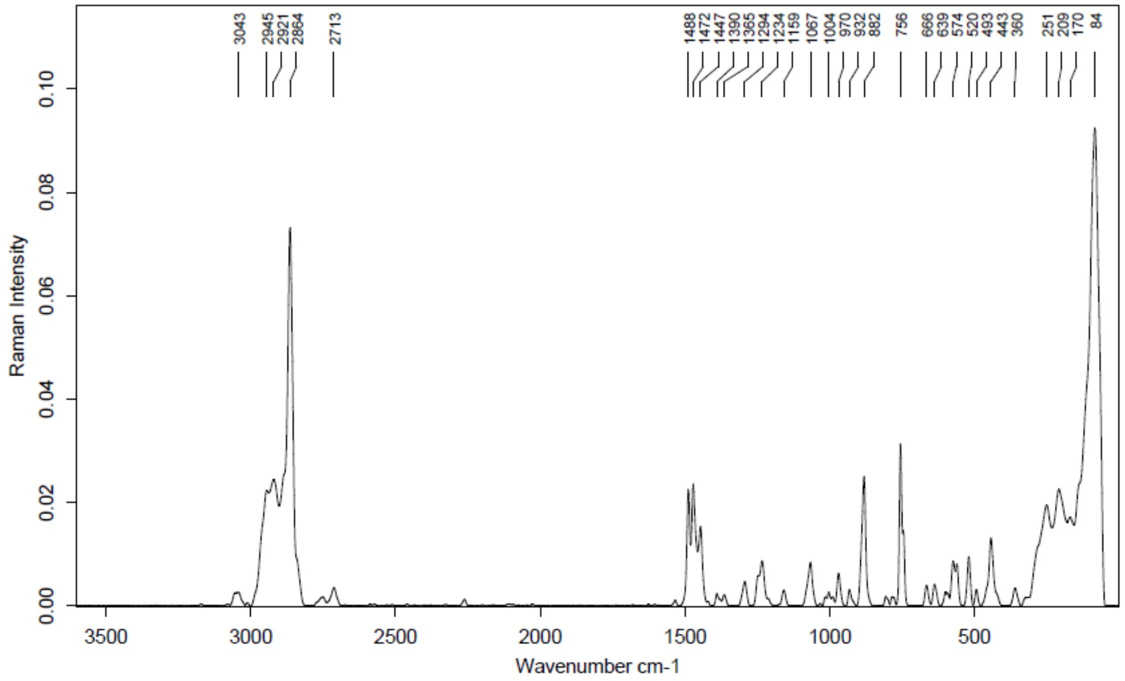

Figure S 18: FT-Raman spectrum of $[Pr^{III}(\eta^8\text{-Cot}^{TIPS})_2K]_n$ (**1-Pr/2-Pr**).

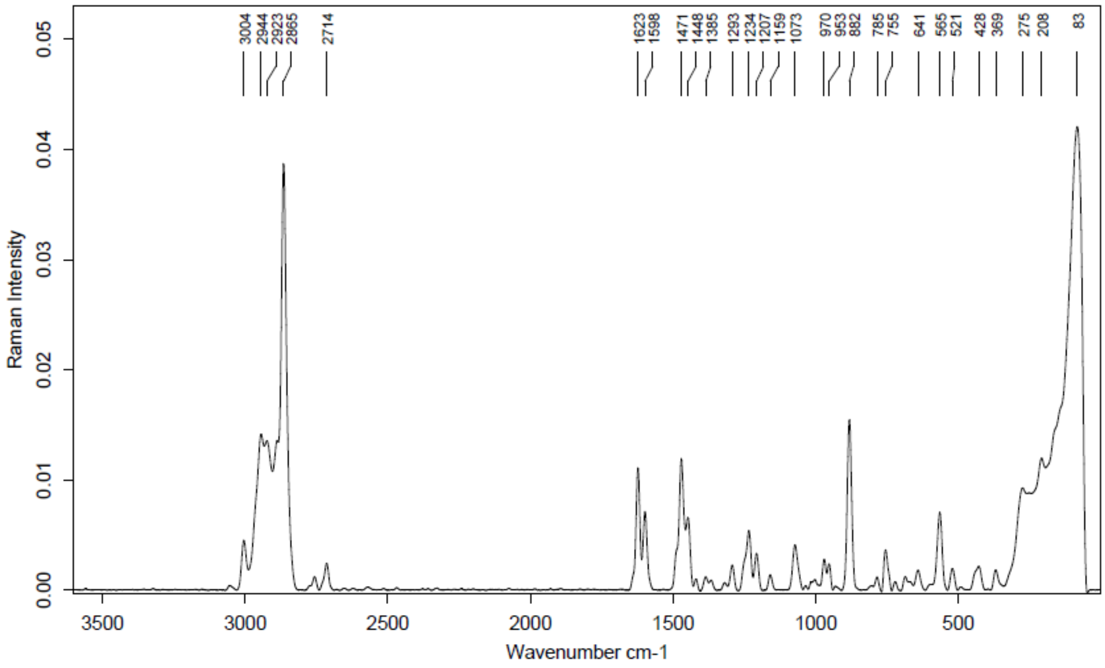

Figure S 19: FT-Raman spectrum of $[Nd^{III}(\eta^8\text{-Cot}^{TIPS})_2K]_n$ (**2-Nd**).

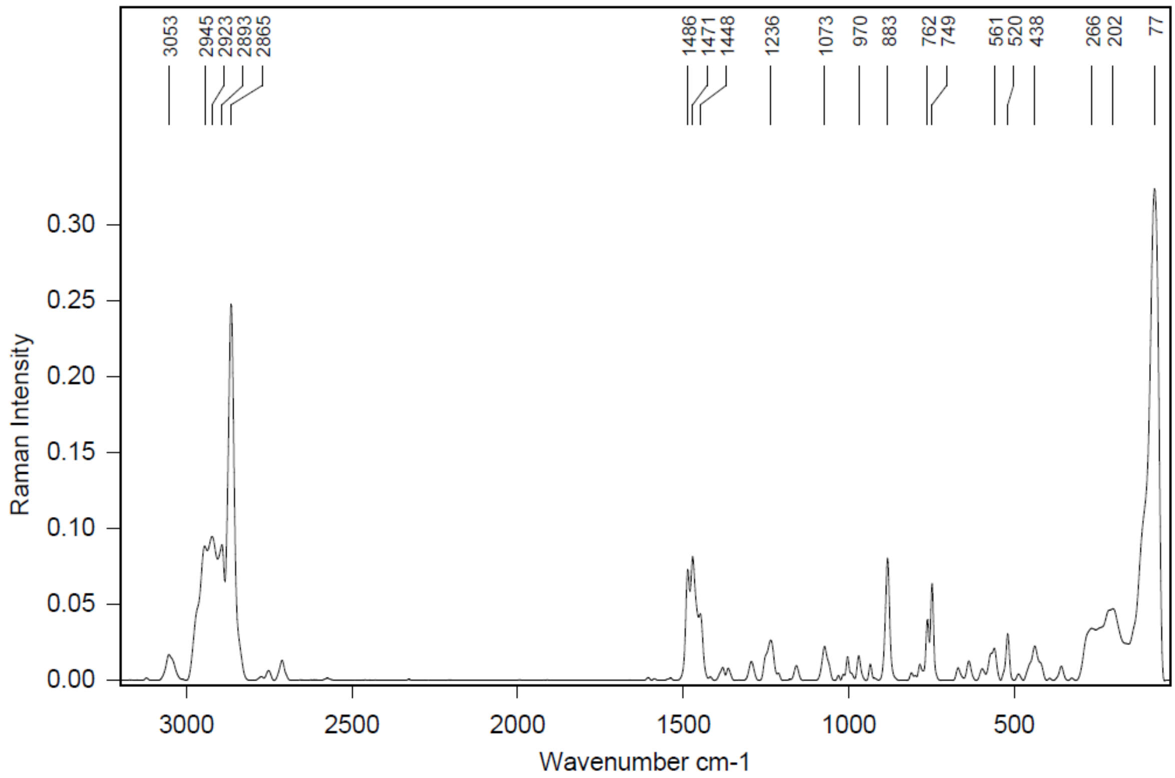

Figure S 20: FT-Raman spectrum of [Er^{III}(η^8-Cot^{TIPS})$_2$K]$_n$ (**2-Er/3-Er**).

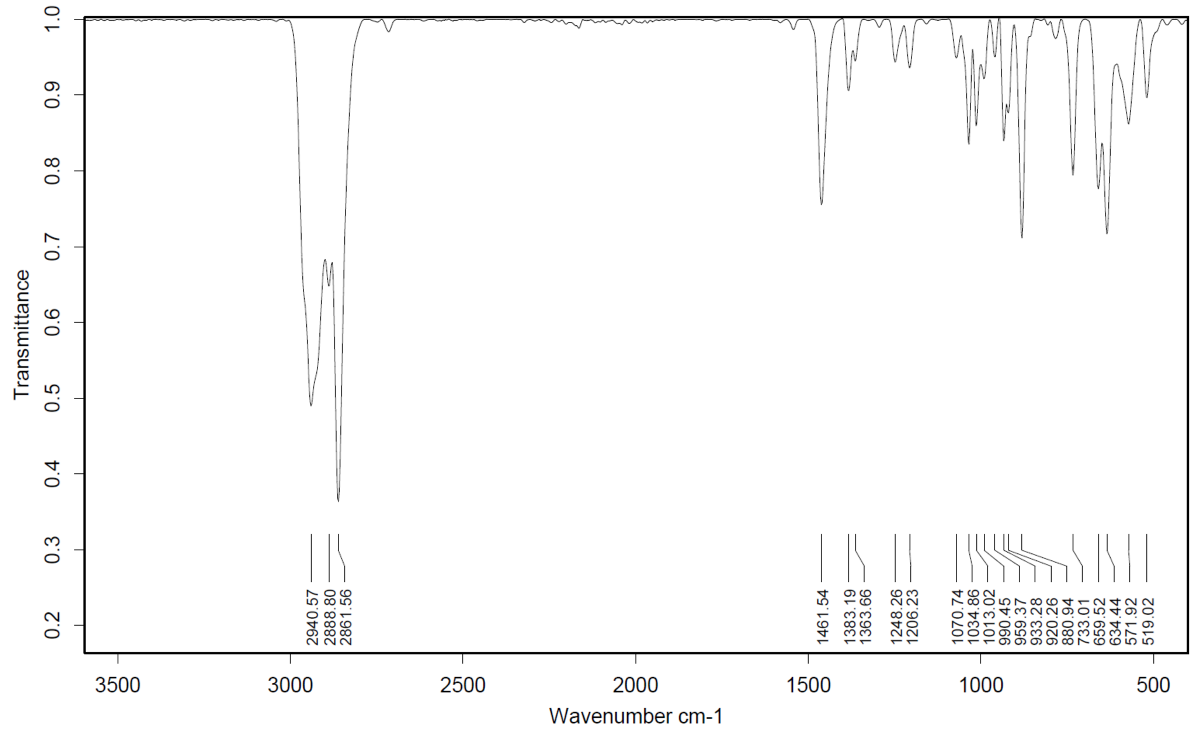

Figure S 21: IR (ATR) spectrum of [Lu^{III}(η^8-Cot^{TIPS})$_2$K]$_n$ (**2-Lu**).

X-ray Crystallographic Studies

Suitable crystals were selected under an optic microscope equipped with polarizing filters, covered in mineral oil (Aldrich) and mounted on a MiTeGen holder. Subsequently, the crystals were directly transferred to the cold stream of a *STOE IPDS 2* (150 K) or *STOE StadiVari* (100 K) diffractometer, equipped with a *Mo-sealed tube* or a *MoGenix 3D HF* X-ray source (Mo-Kα: λ = 0.71073 Å). All structures were solved using the programs SHELXS/T and Olex2 1.3.[3], [4], [5] The remaining non-hydrogen atoms were located from successive difference Fourier map calculations. The refinements were carried out by using full-matrix least-squares techniques on F^2 by using the program SHELXL. In each case, the locations of the largest peaks in the final difference Fourier map calculations, as well as the magnitude of the residual electron densities, were of no chemical significance.

Crystallographic data for the structures reported in this paper have been deposited with the Cambridge Crystallographic Data Centre as a supplementary publication no. 2282875-2282882 and 2309731-2309732. Copies of the data can be obtained free of charge on application to CCDC, 12 Union Road, Cambridge CB21EZ, UK (fax: (+(44)1223-336-033; email: deposit@ccdc.cam.ac.uk).

Table S1: Crystal data and structure refinement.

Compound	1-La	1-Ce	1-Pr	2-La
Formula	$C_{55.5}H_{100}KLaSi_4$	$C_{55.5}H_{100}KCeSi_4$	$C_{118}H_{208}K_2Pr_2Si_8$	$C_{52}H_{96}KLaSi_4$
$D_{calc.}$/ g cm^{-3}	1.201	1.153	1.216	1.202
μ/mm^{-1}	0.880	0.924	0.989	0.955
Formula Weight	1103.79	1058.93	2211.57	1011.65
Colour	yellow	clear dark green	yellow	orange
Shape	block-shaped	block-shaped	fragment-shaped	rod-shaped
Size/mm^3	0.56×0.42×0.29	0.33×0.30×0.24	0.23×0.14×0.09	0.23×0.14×0.07
T/K	150	150	100	180
Crystal System	orthorhombic	orthorhombic	monoclinic	monoclinic
Space Group	$C222_1$	$C222_1$	$P2_1/n$	$P2_1/n$
a/Å	18.1753(3)	18.1753(3)	13.8875(3)	14.9492(4)
b/Å	22.3961(3)	22.3961(3)	33.4663(9)	17.4712(5)
c/Å	29.9843(6)	29.9843(6)	26.0210(6)	21.5754(5)
α/°	-	-	-	-
β/°	-	-	92.638(2)	97.134(2)
γ/°	-	-	-	-
V/Å^3	12205.3(4)	12205.3(4)	12080.8(5)	5591.4(3)
Z	8	8	4	4
Z'	1	1	1	1
Wavelength/Å	0.71073	0.71073	0.71073	0.71073
Radiation type	Mo K_α	Mo K_α	Mo K_α	Mo K_α
Q_{min}/°	1.358	1.358	1.681	2.231
Q_{max}/°	26.737	27.188	29.535	31.843
Measured Refl.	55243	32075	65016	39321
Independent Refl.	12895	13515	29803	16651
Reflections with I≥2 *s*(I)	11014	12463	17854	12095
R_{int}	0.0426	0.0177	0.0459	0.0336
Parameters	812	812	1539	547
Restraints	186	186	424	0
Largest Peak	2.017	0.491	0.984	1.167
Deepest Hole	-0.608	-0.283	-0.943	-0.918
GooF	1.039	1.026	1.063	1.019
wR_2 (all data)	0.1183	0.0685	0.1549	0.1079
wR_2	0.1150	0.0665	0.1323	0.0967
R_1 (all data)	0.0508	0.0338	0.1331	0.0750
R_1	0.0428	0.0287	0.0695	0.0433
Flack Parameter	-0.026(7)	-0.018(4)	-	-
Hooft Parameter	0.037(3)	0.031(2)	-	-

**Compound**	**2-Ce** (0.5 Et_2O)	**2-Pr**	**2-Nd**	**2-Er**
Formula	$C_{54}H_{101}KCeO_{0.5}Si_4$	$C_{52}H_{96}KPrSi_4$	$C_{52}H_{96}KNdSi_4$	$C_{52}H_{95}ErKSi_4$
$D_{calc.}$/ g cm^{-3}	1.145	1.201	1.224	1.253
μ/mm^{-1}	0.926	1.059	1.134	1.717
Formula Weight	1049.92	1013.65	1016.98	1038.99
Colour	dark green	clear yellow	clear green	yellow
Shape	block-shaped	block-shaped	prism-shaped	block-shaped
Size/mm^3	0.56×0.39×0.23	0.24×0.19×0.16	0.15×0.13×0.11	0.33×0.24×0.18
T/K	150	150	100	100
Crystal System	monoclinic	monoclinic	monoclinic	monoclinic
Space Group	$P2_1/c$	$P2_1/n$	$P2_1/n$	$P2_1/n$
a/Å	21.4286(5)	14.9574(4)	14.8785(8)	14.8618(9)
b/Å	15.4560(2)	17.5114(4)	17.3956(7)	17.3571(8)
c/Å	18.8223(4)	21.5769(5)	21.4878(12)	21.5304(17)
α/°	-	-	-	90
β/°	102.374(2)	97.154(2)	97.196(4)	97.390(6)
γ/°	-	-	-	90
V/Å^3	6089.1(2)	5607.5(2)	5517.7(5)	5507.8(6)
Z	4	4	4	4
Z'	1	1	1	1
Wavelength/Å	0.71073	0.71073	0.71073	0.71073
Radiation type	Mo $K_α$	Mo $K_α$	Mo $K_α$	Mo K_a
$Q_{min}/°$	1.638	1.569	2.125	1.908
$Q_{max}/°$	29.541	29.541	31.697	30.070
Measured Refl.	88420	60046	34859	34803
Independent Refl.	16911	15602	14988	13056
Reflections with I≥2 *s*(I)	13243	11333	9628	10975
R_{int}	0.0373	0.0482	0.0509	0.0218
Parameters	594	558	547	546
Restraints	0	0	0	0
Largest Peak	0.951	0.731	2.195	0.720
Deepest Hole	-0.501	-0.532	-1.481	-0.671
GooF	1.040	1.065	1.027	0.954
wR_2 (all data)	0.0894	0.0792	0.1452	0.0604
wR_2	0.0806	0.0684	0.1191	0.0587
R_1 (all data)	0.0493	0.0743	0.1061	0.0305
R_1	0.0318	0.0393	0.0555	0.0226

**Compound**	**3-Er**	**2-Lu**
Formula	$C_{59}H_{104}KErSi_4$	$C_{52}H_{96}KLuSi_4$
$D_{calc.}$/ g cm^{-3}	1.243	1.265
μ/mm^{-1}	1.569	1.989
Formula Weight	1132.14	1047.71
Colour	yellow	colourless
Shape	fragment-shaped	block-shaped
Size/mm^3	0.69×0.41×0.24	0.20×0.12×0.06
T/K	150	130
Crystal System	orthorhombic	monoclinic
Space Group	*F2dd*	$P2_1/n$
a/Å	16.0025(3)	14.843(2)
b/Å	32.4257(7)	17.3279(17)
c/Å	46.6302(7)	21.557(2)
α/°	-	90
β/°	-	97.330(10)
γ/°	-	90
V/Å^3	24196.1(8)	5499.3(11)
Z	16	4
Z'	1	1
Wavelength/Å	0.71073	0.71073
Radiation type	Mo K_α	Mo K□
$Q_{min}/°$	1.485	1.815
$Q_{max}/°$	26.734	30.188
Measured Refl.	52731	34000
Independent Refl.	12760	13139
Reflections with I≥2 *s*(I)	11495	8578
R_{int}	0.0211	0.0420
Parameters	691	547
Restraints	116	13
Largest Peak	0.846	0.661
Deepest Hole	-0.463	-0.937
GooF	1.002	0.885
wR_2 (all data)	0.0592	0.0602
wR_2	0.0584	0.0555
R_1 (all data)	0.0282	0.0682
R_1	0.0239	0.0327
Flack Parameter	0.174(6)	
Hooft Parameter	0.005(2)	

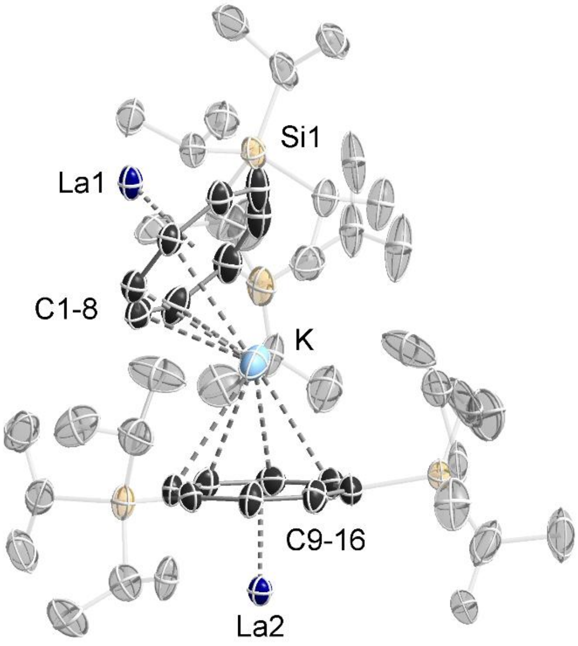

Figure S 22: Asymmetric unit of **1-La**. Thermal ellipsoids are represented at 50% probability. Hydrogen atoms are omitted, and TIPS groups are transparent for better clarity. Only one part of the two disordered TIPS groups is depicted. For selected bond lengths and angles see table S2. Colour code: La (dark blue), K (light blue), Si (transparent orange), C (black).

Table S2: Selected bond lengths and angles of $[La^{III}(\eta^8\text{-}Cot^{TIPS})_2K]_n$ (**1-La**).

**Length/ Å**			**Angle/°**			
La1	Ct_{C1-8}	2.124(9)	La1	Ct_{C1-8}	K	163.15(4)
La2	Ct_{C9-16}	2.1245(1)	Ct_{C1-8}	K	Ct_{C9-16}	149.98(7)
K	Ct_{C1-8}	2.855(2)	K	Ct_{C9-16}	La2	168.82(4)
K	Ct_{C9-16}	2.683(2)				

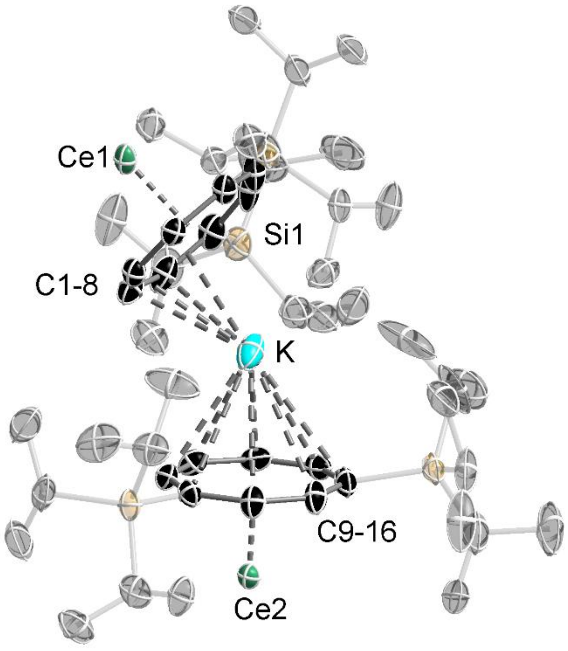

Figure S 23: Asymmetric unit of **1-Ce**. Thermal ellipsoids are represented at 50% probability. Hydrogen atoms are omitted, and TIPS groups are transparent for better clarity. Only one part of the two disordered TIPS groups is depicted. For selected bond lengths and angles see table S3. Colour code: Ce (green), K (light blue), Si (transparent orange), C (black).

Table S 3: Selected bond lengths and angles of $[Ce^{III}(\eta^8\text{-}Cot^{TIPS})_2K]_n$ (**1-Ce**).

**Length/ Å**			**Angle/°**			
Ce1	$Ct_{C1\text{-}8}$	2.090(3)	Ce1	$Ct_{C1\text{-}8}$	K	162.25(2)
Ce2	$Ct_{C9\text{-}16}$	2.0913(1)	$Ct_{C1\text{-}8}$	K	$Ct_{C9\text{-}16}$	150.85(4)
K	$Ct_{C1\text{-}8}$	2.8839(10)	K	$Ct_{C9\text{-}16}$	Ce2	167.89(2)
K	$Ct_{C9\text{-}16}$	2.698(1)				

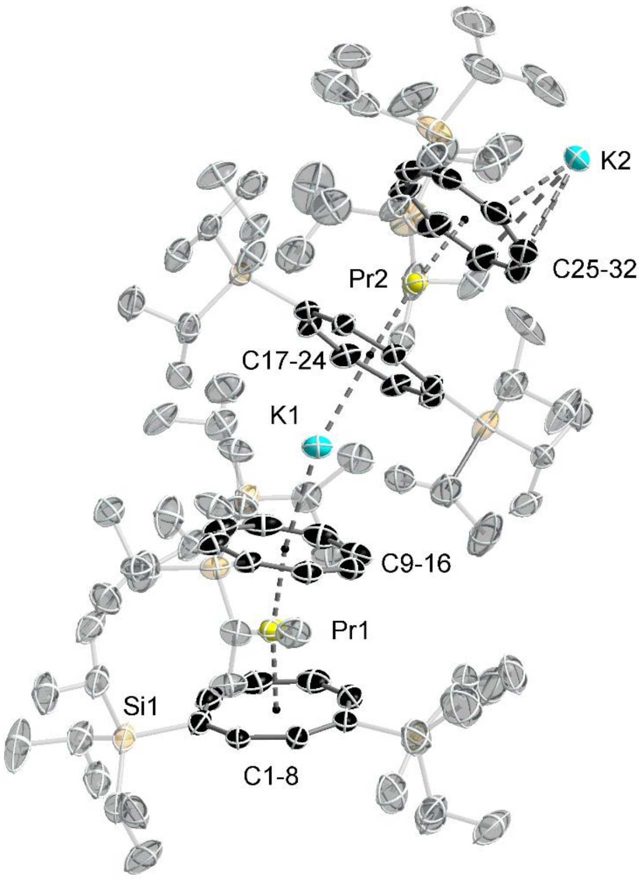

Figure S 24: Asymmetric unit of **1-Pr**. Thermal ellipsoids are represented at 50% probability. Hydrogen atoms are omitted, and TIPS groups are transparent for better clarity. Only one part of the four disordered TIPS groups is depicted. For selected bond lengths and angles see table S4. Colour code: Pr (yellow), K (light blue), Si (transparent orange), C (black).

Table S 4: Selected bond lengths and angles of $[Pr^{III}(\eta^8\text{-}Cot^{TIPS})_2K]_n$ (**1-Pr**).

**Length/ Å**			**Angle/°**			
Pr1	Ct_{C1-8}	2.0772(6)	Ct_{C1-8}	Pr1	Ct_{C9-16}	167.21(3)
Pr1	Ct_{C9-16}	2.0548(6)	Pr1	Ct_{C9-16}	K1	171.11(3)
K1	Ct_{C9-16}	2.6505(13)	Ct_{C9-16}	K1	Ct_{C17-24}	157.41(6)
K1	Ct_{C17-24}	2.5620(13)	K1	Ct_{C17-24}	Pr2	173.51(4)
Pr2	Ct_{C17-24}	2.0726(6)	Ct_{C17-24}	Pr2	Ct_{C25-32}	171.24(4)
Pr2	Ct_{C25-32}	2.0539(6)	Pr2	Ct_{C25-32}	K2	156.25(3)
K2	Ct_{C25-32}	3.1555(13)				

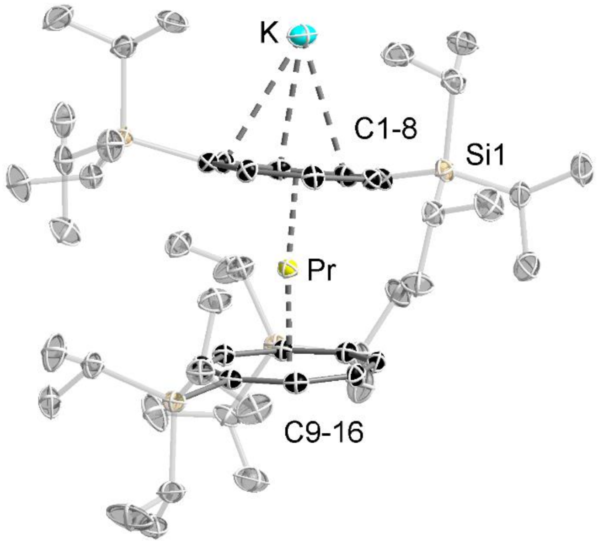

Figure S 25: Asymmetric unit of **2-Pr**. Thermal ellipsoids are represented at 50% probability. Hydrogen atoms are omitted, and TIPS groups are transparent for better clarity. For selected bond lengths and angles see table S5. Colour code: Pr (yellow), K (light blue), Si (transparent orange), C (black).

Table S 5: Selected bond lengths and angles of $[Pr^{III}(\eta^8\text{-}Cot^{TIPS})_2K]_n$ (**2-Pr**).

**Length/ Å**			**Angle/°**			
Pr	$Ct_{C1\text{-}8}$	2.0359(4)	$Ct_{C1\text{-}8}$	Pr	$Ct_{C9\text{-}16}$	170.02(2)
Pr	$Ct_{C9\text{-}16}$	2.0732(4)	Pr	$Ct_{C1\text{-}8}$	K	150.52(2)
K	$Ct_{C1\text{-}8}$	3.2220(7)				

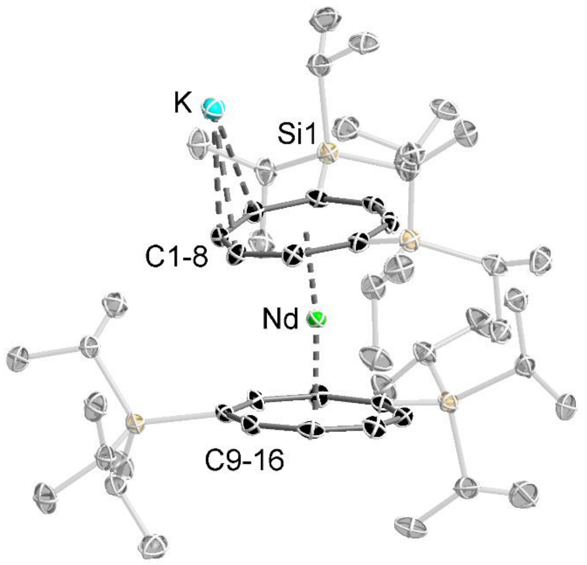

Figure S 26: Asymmetric unit of **2-Nd**. Thermal ellipsoids are represented at 50% probability. Hydrogen atoms are omitted, and TIPS groups are transparent for better clarity. For selected bond lengths and angles see table S6. Colour code: Nd (green), K (light blue), Si (transparent orange), C (black).

Table S 6: Selected bond lengths and angles of $[Nd^{III}(\eta^8\text{-}Cot^{TIPS})_2K]_n$ (**2-Nd**).

**Length/ Å**			**Angle/°**			
Nd	Ct_{C1-8}	1.9986(4)	Ct_{C1-8}	Nd	Ct_{C9-16}	170.50(2)
Nd	Ct_{C9-16}	2.0385(4)	Nd	Ct_{C1-8}	K	149.89(2)
K	Ct_{C1-8}	3.2356(10)				

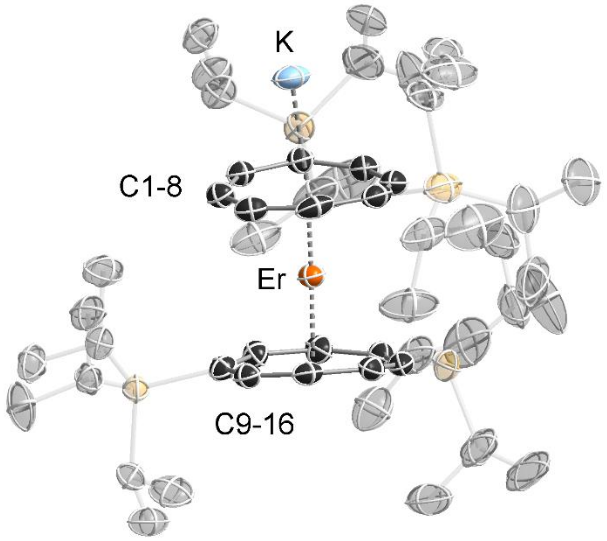

Figure S 27: Asymmetric unit of **3-Er**. Thermal ellipsoids are represented at 50% probability. Hydrogen atoms are omitted, and TIPS groups are transparent for better clarity. For selected bond lengths and angles see table S7. Colour code: Er (orange), K (light blue), Si (transparent orange), C (black).

Table S 7: Selected bond lengths and angles of $[Er^{III}(\eta^8\text{-}Cot^{TIPS})_2K]_n$ (**3-Er**).

**Length/ Å**			**Angle/°**			
Er	$Ct_{C1\text{-}8}$	1.9405(6)	$Ct_{C1\text{-}8}$	Er	$Ct_{C9\text{-}16}$	173.62(3)
Er	$Ct_{C9\text{-}16}$	1.8679(6)	Er	$Ct_{C1\text{-}8}$	K	173.73(3)
K	$Ct_{C1\text{-}8}$	2.4212(10)				

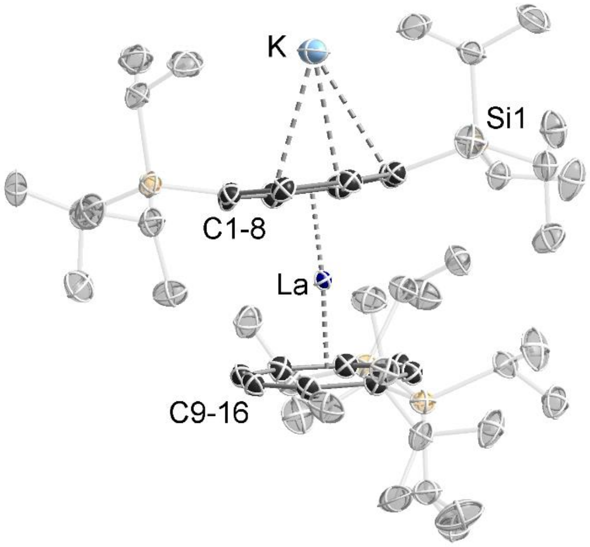

Figure S 28: Asymmetric unit of **2-La**. Thermal ellipsoids are represented at 50% probability. Hydrogen atoms are omitted, and TIPS groups are transparent for better clarity. For selected bond lengths and angles see table S8. Colour code: La (dark blue), K (light blue), Si (transparent orange), C (black).

Table S 8: Selected bond lengths and angles of [La^{III}(η^8-Cot^{TIPS})$_2$K]$_n$ (**2-La**).

**Length/ Å**			**Angle/°**			
La	Ct$_{C1-8}$	1.9740(4)	Ct$_{C1-8}$	La	Ct$_{C9-16}$	171.34(2)
La	Ct$_{C9-16}$	2.0148(4)	La	Ct$_{C1-8}$	K	149.16(2)
K	Ct$_{C1-8}$	3.2808(7)				

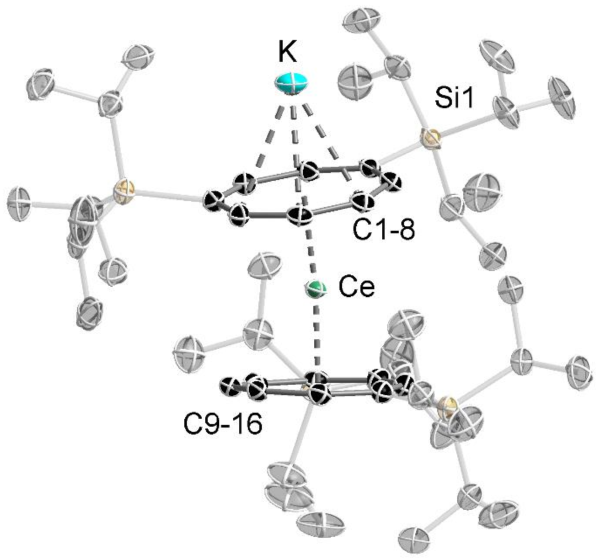

Figure S 29: Asymmetric unit of **2-Ce**. Thermal ellipsoids are represented at 50% probability. Hydrogen atoms are omitted, and TIPS groups are transparent for better clarity. For selected bond lengths and angles see table S9. Colour code: Ce (green), K (light blue), Si (transparent orange), C (black).

Table S 9: Selected bond lengths and angles of $[Ce^{III}(\eta^8\text{-}Cot^{TIPS})_2K]_n$ (**2-Ce**).

**Length/ Å**			**Angle/°**			
Ce	Ct_{C1-8}	2.1026(4)	Ct_{C1-8}	Ce	Ct_{C9-16}	169.71(2)
Ce	Ct_{C9-16}	2.0526(4)	Ce	Ct_{C1-8}	K	173.95(2)
K	Ct_{C1-8}	2.4540(6)				

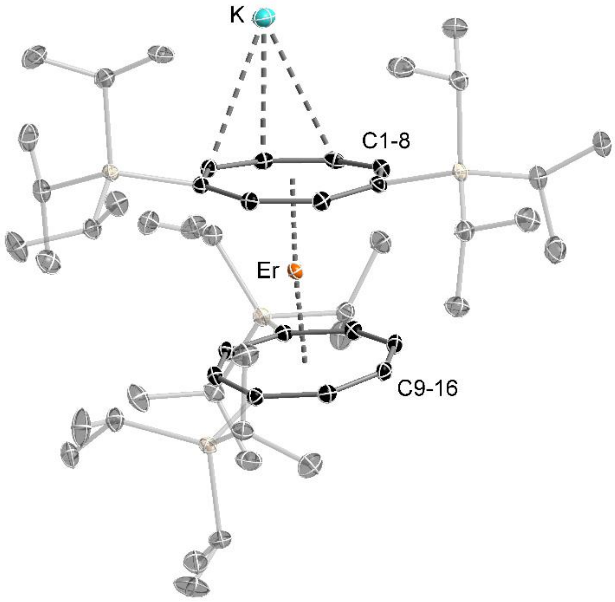

Figure S 30: Asymmetric unit of **2-Er**. Thermal ellipsoids are represented at 50% probability. Hydrogen atoms are omitted, and TIPS groups are transparent for better clarity. For selected bond lengths and angles see table S6. Colour code: Nd (green), K (light blue), Si (transparent orange), C (black).

Table S 10: Selected bond lengths and angles of $[Er^{III}(\eta^8\text{-}Cot^{TIPS})_2K]_n$ (**2-Er**).

**Length/ Å**			**Angle/°**			
Er	Ct_{C1-8}	1.8709(4)	Ct_{C1-8}	Er	Ct_{C9-16}	172.267(22)
Er	Ct_{C9-16}	1.9231(4)	Er	Ct_{C1-8}	K	147.363(12)
K	Ct_{C1-8}	3.3595(4)				

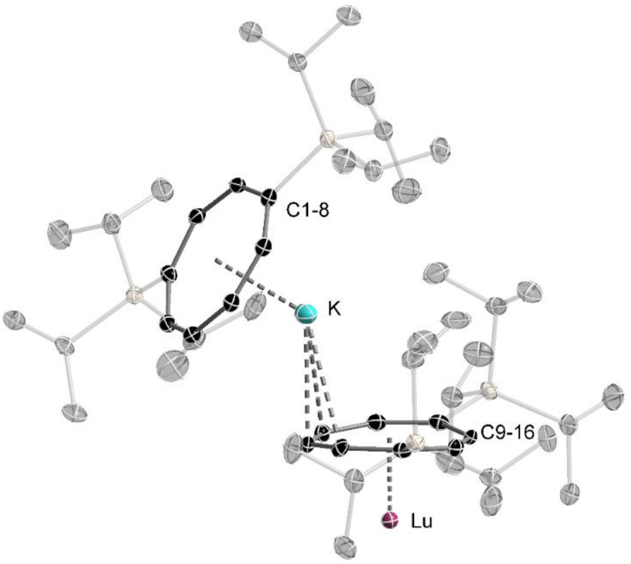

Figure S 31: Asymmetric unit of **2-Lu**. Thermal ellipsoids are represented at 50% probability. Hydrogen atoms are omitted, and TIPS groups are transparent for better clarity. For selected bond lengths and angles see table S9. Colour code: Lu (purple), K (light blue), Si (transparent orange), C (black).

Table S 11: Selected bond lengths and angles of [Lu^{III}(η^8-Cot^{TIPS})$_2$K]$_n$ (**2-Lu**).

**Length/ Å**			**Angle/°**			
Lu	Ct$_{C1-8}$	1.8947(4)	Ct$_{C1-8}$	Lu	Ct$_{C9-16}$	172.47(2)
Lu	Ct$_{C9-16}$	1.8349(4)	Lu	Ct$_{C1-8}$	K	175.02(2)
K	Ct$_{C1-8}$	2.5115(7)				

PL/PLE Spectra

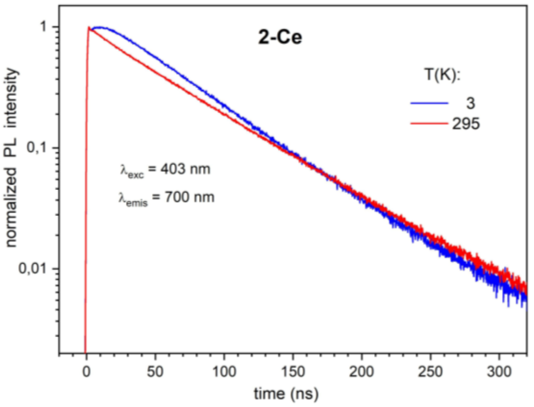

Figure S 32: Emission decay of polycrystalline helix and zigzag $[Ce^{III}(\eta^8\text{-}Cot^{TIPS})_2K]_n$ polymers (**1-Ce** and **2-Ce**, respectively) at different temperatures within 3 - 295 K. The emission was excited with a sub-ns laser diode at 403 nm and recorded at 700 nm. The instrument response is shown as the cyan-colored transient. Monoexponential decay fits yield lifetimes of 48 and 50 ns for **1**-**Ce**, 56 and 62 ns for **2-Ce**, at 3 K and 295 K, respectively.

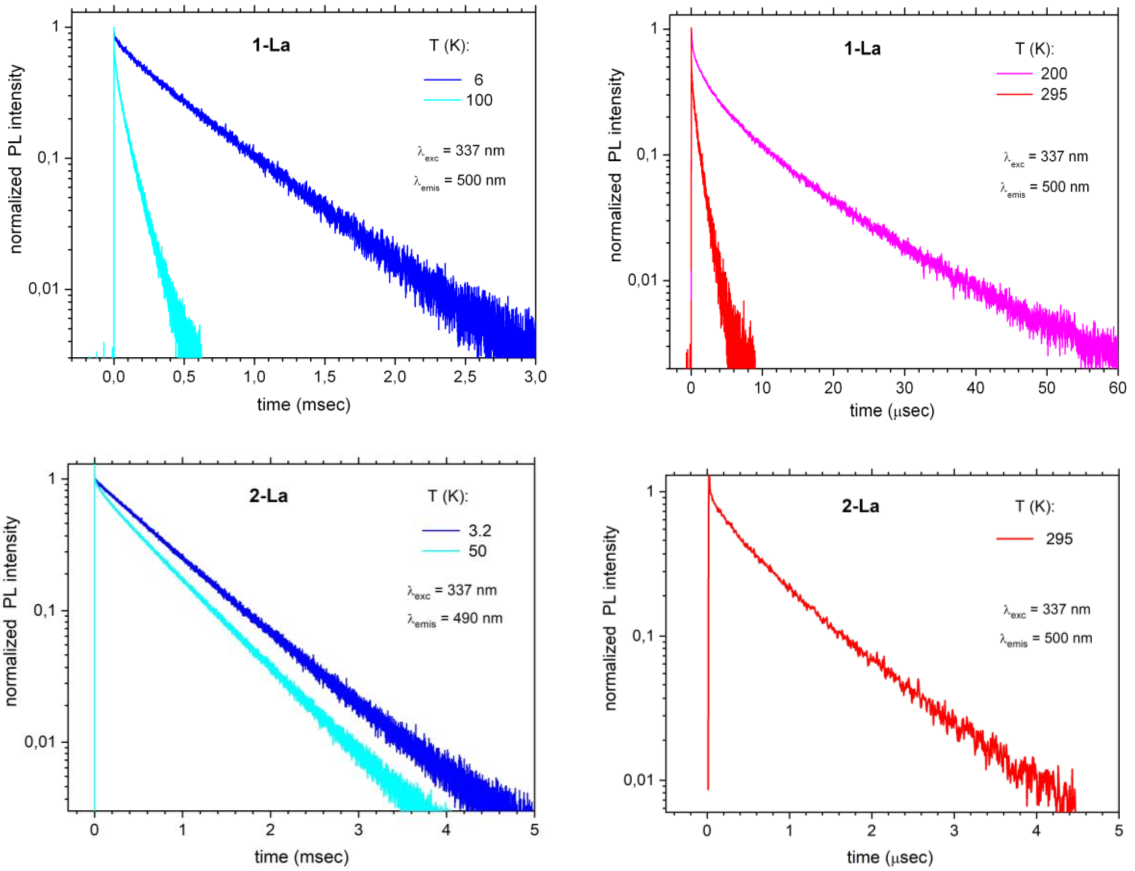

Figure S 33: Emission decay of polycrystalline **1-La** and **2-La** at different temperatures. The emission was excited with a ns-pulsed N_2 laser at 337 and recorded at 500 (490) nm. Average lifetimes from biexponential fits are 450, 66, 5.3 and 0.8 μs for **1-La** at 6, 100, 200 and 295 K, respectively, and 0.65 μs for **2-La** at 295 K. At low temperatures, PL decay of **2-La** reasonably follows the monoexponential kinetics with τ = 730 and 590 μs at 3 and 50 K, respectively.

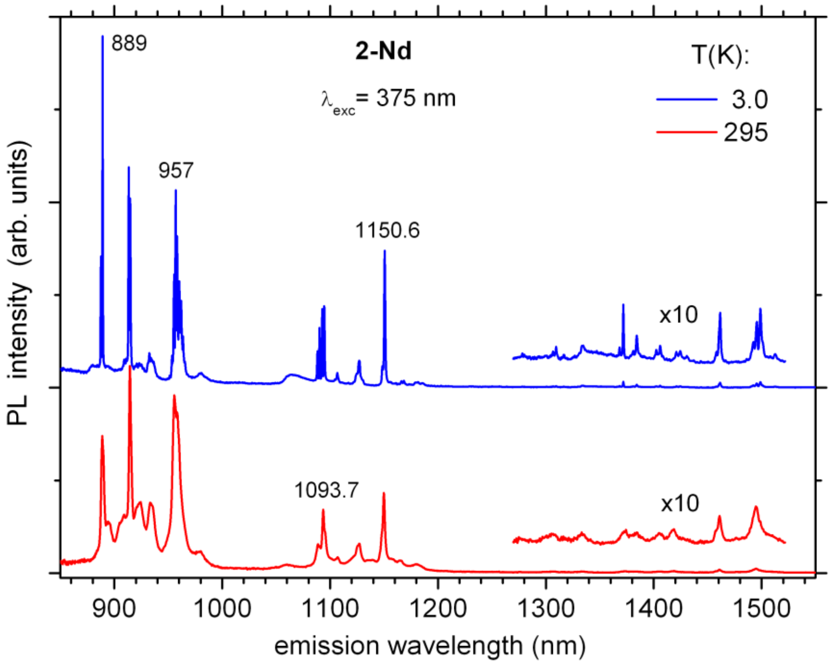

Figure S 34: FTIR-PL emission spectra of Nd^{3+} ions in polycrytalline $[Nd^{III}(\eta^8\text{-}Cot^{TIPS})_2K]_n$ (**2-Nd**) at T = 3 and 295 K and laser excitation at.375 nm (5 mW).

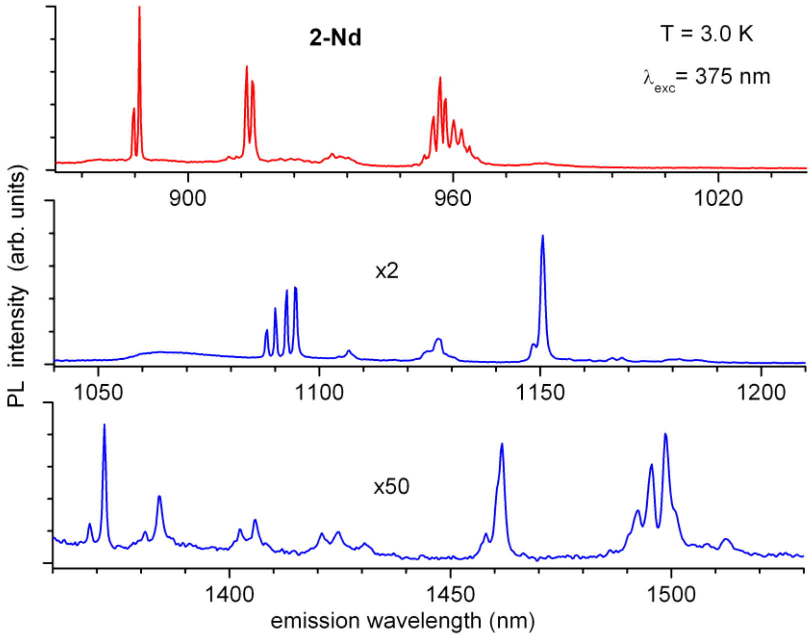

Figure S 35: Fine structure of the near-infrared emission bands of of Nd^{3+} ions in **2-Nd** (see previous Figure) at T = 3.0 K. The spectrum was acquired with an FTIR-PL spectrometer (see experimental details) at spectral resolution of 1 cm^{-1}.

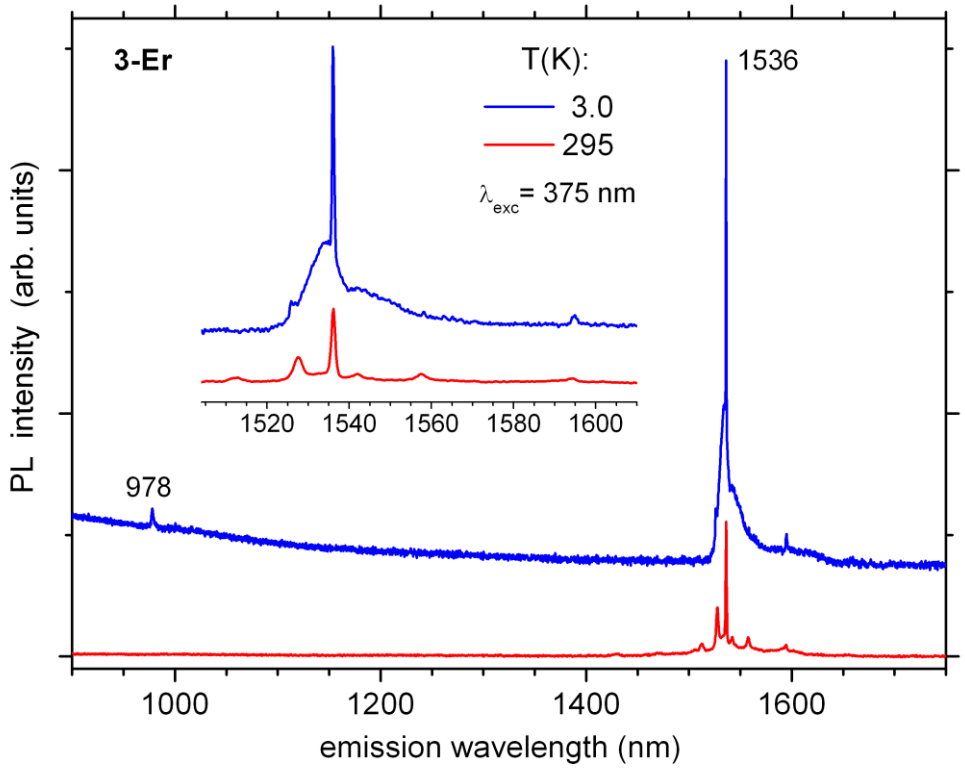

Figure S 36: FTIR-PL emission spectra of Er^{3+} ions in polycrytalline $[Er^{III}(\eta^8\text{-}Cot^{TIPS})_2K]_n$ (**3-Er**) at T = 3 and 295 K. The sample in the cryostat was excited with an (unfocused) laser beam at 375 nm (5 mW). The spectral resolution corresponded to 1 and 2 cm^{-1} at 3 and 295 K, respectively.

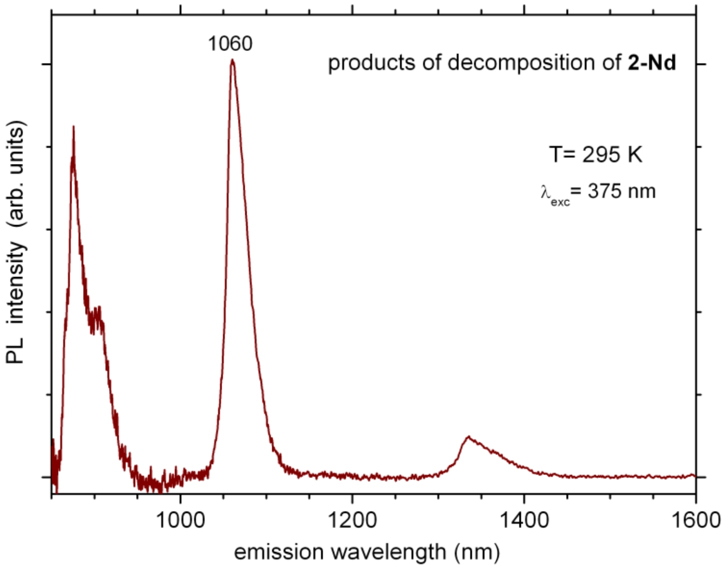

Figure S 37: FTIR-PL emission spectrum of Nd^{3+} ions in decomposition products of **2-Nd** after ~1 day exposure of this coordination polymer (as polycrystalline powder) to air at ambient temperature. The spectrum is acquired at 295 K using laser excitation at.375 nm (5 mW).

Magnetism

All magnetic measurements were performed on polycrystalline samples of the complexes. Due to their sensitive nature, the samples were flame sealed in a glass tube under inert atmosphere together with small amounts of eicosane to prevent sample movement. AC and T-dependent susceptibility measurements were carried out using a Quantum Design MPMS-XL Squid magnetometer. Field-dependent measurements of the magnetization were carried out using a Quantum Design MPMS-3 VSM Squid magnetometer. All data have been corrected for diamagnetic contributions of the sampleholder, the eicosane and the sample.
The mass of the samples/ eicosane were 10/ 30 mg (**1-Ce**), 11/ 27 mg (**2-Ce**), 9/ 27 mg (**2-Pr**), 13/ 27 mg (**2-Nd**), 10/ 29 mg (**2-Er**), 20/ 30 mg (**3-Er**).

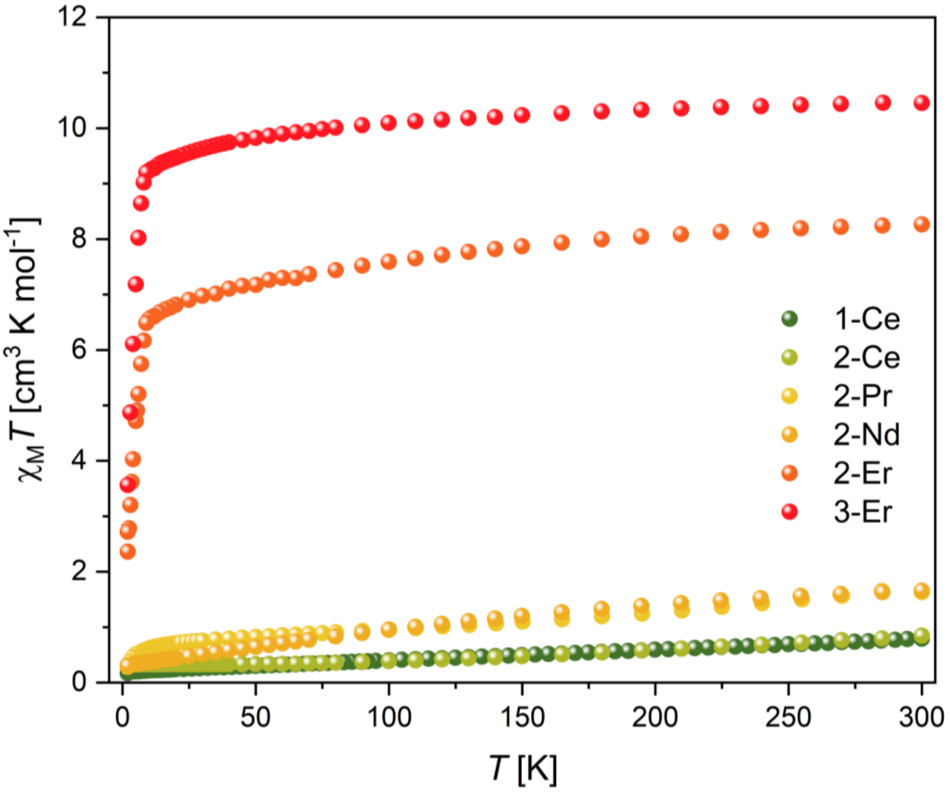

Figure S 38: Temperature product of the magnetic susceptibility vs. temperature recorded at 1000 Oe for all the complexes.

Table S 12: Best Fit parameters obtained for [Ce^{III}(η^8-Cot^{TIPS})$_2$K]$_n$ (**1-Ce**).

Temperature [K]	X_T [cm^3 mol^{-1}]	X_S [cm^3 mol^{-1}]	τ [s]	α
2.0	0.00221	0.10617	2.73913E-4	0.20099
2.4	0.00165	0.09973	2.52414E-4	0.20238
2.6	0.00126	0.09284	2.20436E-4	0.16966
2.8	0.00122	0.0871	2.51872E-4	0.20286
3.0	0.0068	0.08029	2.5214E-4	0.13026
3.2	7.69E-4	0.07334	1.91677E-4	0.11988
3.4	0.00825	0.0689	1.45024E-4	0.20419
3.6	0.01749	0.0654	1.38629E-4	0.21374
3.8	0.00282	0.06212	2.18208E-4	0.08235
4.0	3.116E-4	0.0579	1.7527E-4	0.10385
4.4	6.61E-5	0.05542	1.63116E-4	0.09858
4.8	6.79113E-4	0.05115	1.57975E-4	0.07411
5.2	0.00749	0.04735	2.00188E-4	0.02802
5.6	0.00938	0.04478	1.65615E-4	0.01777

Table S 13: Best Fit parameters obtained for $[Ce^{III}(\eta^8\text{-}Cot^{TIPS})_2K]_n$ (**2-Ce**).

Temperature [K]	X_T [cm^3 mol^{-1}]	X_S [cm^3 mol^{-1}]	τ [s]	α
2.0	-0.002	0.15846	8.39424E-4	0.15383
2.4	0.00877	0.13623	8.12582E-4	0.04836
2.6	0.00347	0.12759	7.72335E-4	0.10077
2.8	6.71213E-4	0.11731	7.85931E-4	0.09919
3.0	-0.00143	0.11173	7.65668E-4	0.12355
3.2	-0.00395	0.10422	7.43569E-4	0.17803
3.4	0.00137	0.09796	7.42091E-4	0.14284
3.6	-0.00459	0.09387	7.00397E-4	0.17853
3.8	-0.00437	0.08933	6.88568E-4	0.19294
4.0	-0.00449	0.08554	6.5912E-4	0.19303
4.4	3.70435E-4	0.07678	6.0608E-4	0.14396
4.8	-0.00222	0.0714	5.11865E-4	0.16108
5.2	2.07612E-4	0.06673	4.83689E-4	0.11687
5.6	-0.0047	0.06213	3.95593E-4	0.19785
6.0	0.00276	0.05733	4.13427E-4	0.11559
6.4	-0.00701	0.0539	2.76788E-4	0.19251
6.8	-0.00105	0.05167	2.54886E-4	0.10268
7.2	3.20022E-4	0.04897	1.97637E-4	0.0892
7.6	-0.00446	0.04611	1.51222E-4	0.10152
8.4	-0.01502	0.03974	5.54649E-5	0.19621

Table S 14: Best Fit parameters obtained for $[Nd^{III}(\eta^8\text{-}Cot^{TIPS})_2K]_n$ (**2-Nd**).

Temperature [K]	X_T [cm^3 mol^{-1}]	X_S [cm^3 mol^{-1}]	τ [s]	α
2.0	0.01895	0.15156	0.52576	0.28248
2.2	0.01938	0.13695	0.45215	0.25668
2.4	0.01859	0.12758	0.40572	0.23885
2.6	0.01731	0.1203	0.35047	0.25459
2.8	0.01624	0.11219	0.28999	0.24938
3.0	0.01732	0.10188	0.23672	0.19508
3.2	0.01578	0.09504	0.19062	0.16077
3.4	0.01426	0.08899	0.13237	0.14094
3.6	0.01412	0.08462	0.10762	0.13794
3.8	0.01275	0.08012	0.08142	0.11479
4.0	0.01362	0.07594	0.06184	0.0843
4.4	0.01183	0.06962	0.03685	0.07266
4.8	0.01168	0.06428	0.02227	0.04395
5.2	0.01054	0.05947	0.01423	0.01245
5.6	0.0097	0.05654	0.00789	0.06208
6.0	0.00984	0.053	0.00519	0.02373
6.4	0.00923	0.05023	0.00349	0.07526
6.8	0.00953	0.04771	0.00267	0.01179
7.2	0.00933	0.04548	0.00186	0.0204
7.6	0.01074	0.04348	0.00152	0.01566
8.0	0.00959	0.04135	0.00107	0.01911
8.4	0.00971	0.03982	7.9113E-4	0.02327
8.8	0.00838	0.0381	7.36625E-4	0.13207

Table S 15: Best Fit parameters obtained for $[Er^{III}(\eta^8\text{-}Cot^{TIPS})_2K]_n$ (**2-Er**).

**Temperature [K]**	χ_T **[cm³ mol⁻¹]**	χ_S **[cm³ mol⁻¹]**	**τ [s]**	**α**
10	0.11683	0.75735	2.46277	0.23024
11	0.10832	0.58897	0.52458	0.10135
12	0.09935	0.53835	0.15645	0.08011
13	0.09232	0.50596	0.0548	0.08297
14	0.08786	0.4755	0.02091	0.08637
15	0.08295	0.44728	0.00854	0.09027
16	0.07718	0.4214	0.00383	0.08626
17	0.07127	0.3983	0.00187	0.08906
18	0.06441	0.37825	9.38507E-4	0.10409
19	0.06303	0.35964	5.17435E-4	0.08869
20	0.05864	0.34259	2.98931E-4	0.08608
21	0.04356	0.32681	1.6617E-4	0.10331
22	0.03975	0.31278	1.01362E-4	0.10961
23	0.0332	0.30059	6.45293E-5	0.09519

Table S 16: Best Fit parameters obtained for $[Er^{III}(\eta^8\text{-}Cot^{TIPS})_2K]_n$ (**3-Er**).

**Temperature [K]**	$\chi_{T\ M}$ **[cm³ mol⁻¹]**	$\chi_{T\ S}$ **[cm³ mol⁻¹]**	**τ [s]**	**α**
9	1.23427	0.10152	1.47875	0.33321
10	1.00241	0.09826	0.5617	0.20686
11	0.85735	0.09021	0.17461	0.12671
12	0.80305	0.08375	0.08255	0.11796
13	0.74009	0.07521	0.02867	0.12175
14	0.68802	0.06824	0.01077	0.12702
15	0.62575	0.06023	0.00308	0.12896
16	0.58759	0.05571	0.00137	0.13156
18	0.52414	0.0479	3.42722E-4	0.12973
20	0.47259	0.06493	1.21095E-4	0.09941
22	0.43014	0.12522	6.3779E-5	0.02543

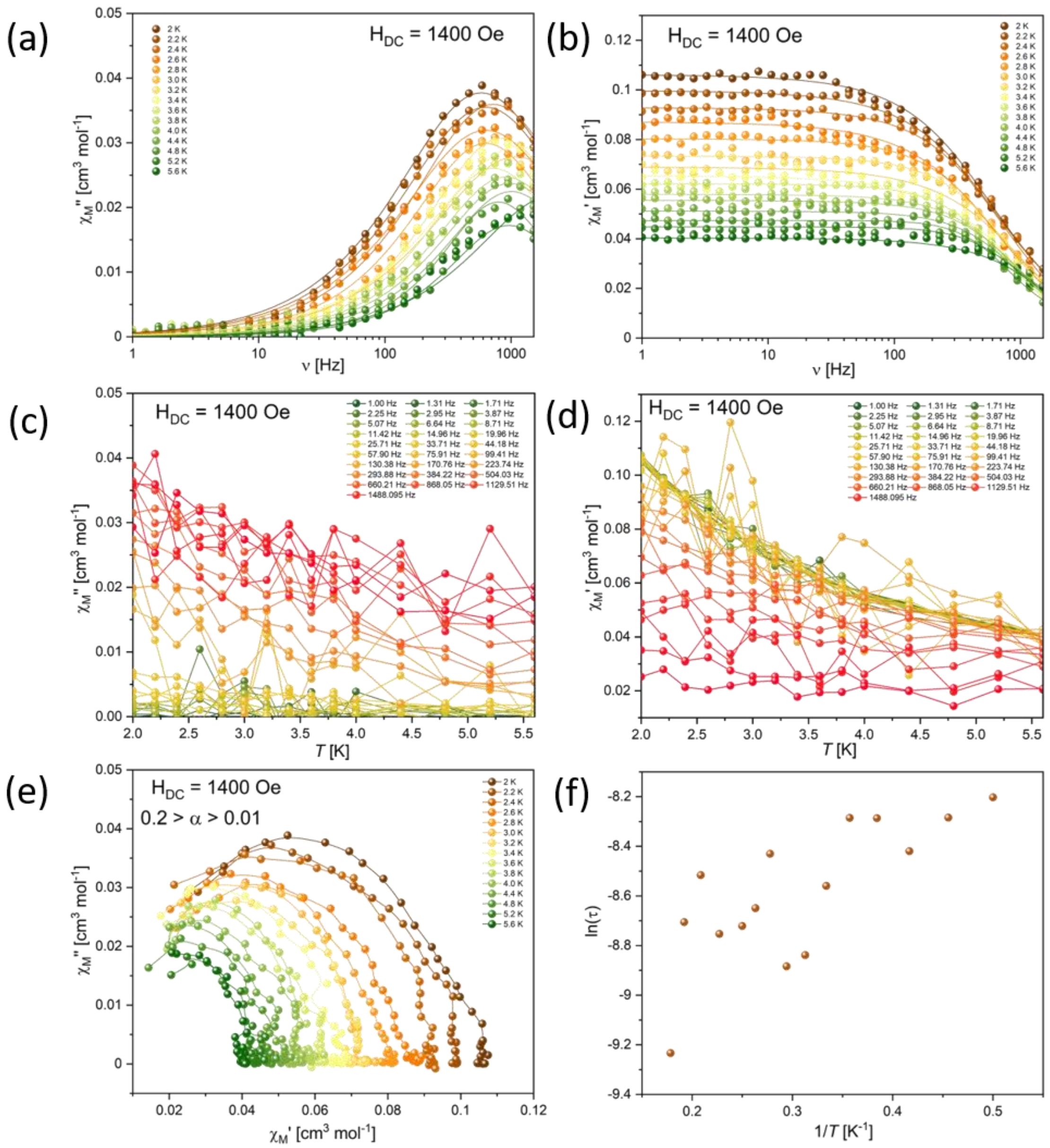

Figure S 39: Dynamic magnetic data for $[Ce^{III}(\eta^8\text{-}Cot^{TIPS})_2K]_n$ (**1-Ce**) employing a 3.5 Oe AC field and 1400 Oe DC field. (a) χ_M'' (ν); b) χ_M'(ν); (c) $\chi_M''(T)$; (d) $\chi_M'(T)$; (e) Cole-cole; (d) Arrhenius plot. The solid lines are a guide for the eye. For (a) and (b), the solid line are fit to a generalized debeye model. For (c), (d) and (e), the solid lines are a guide for the eye.

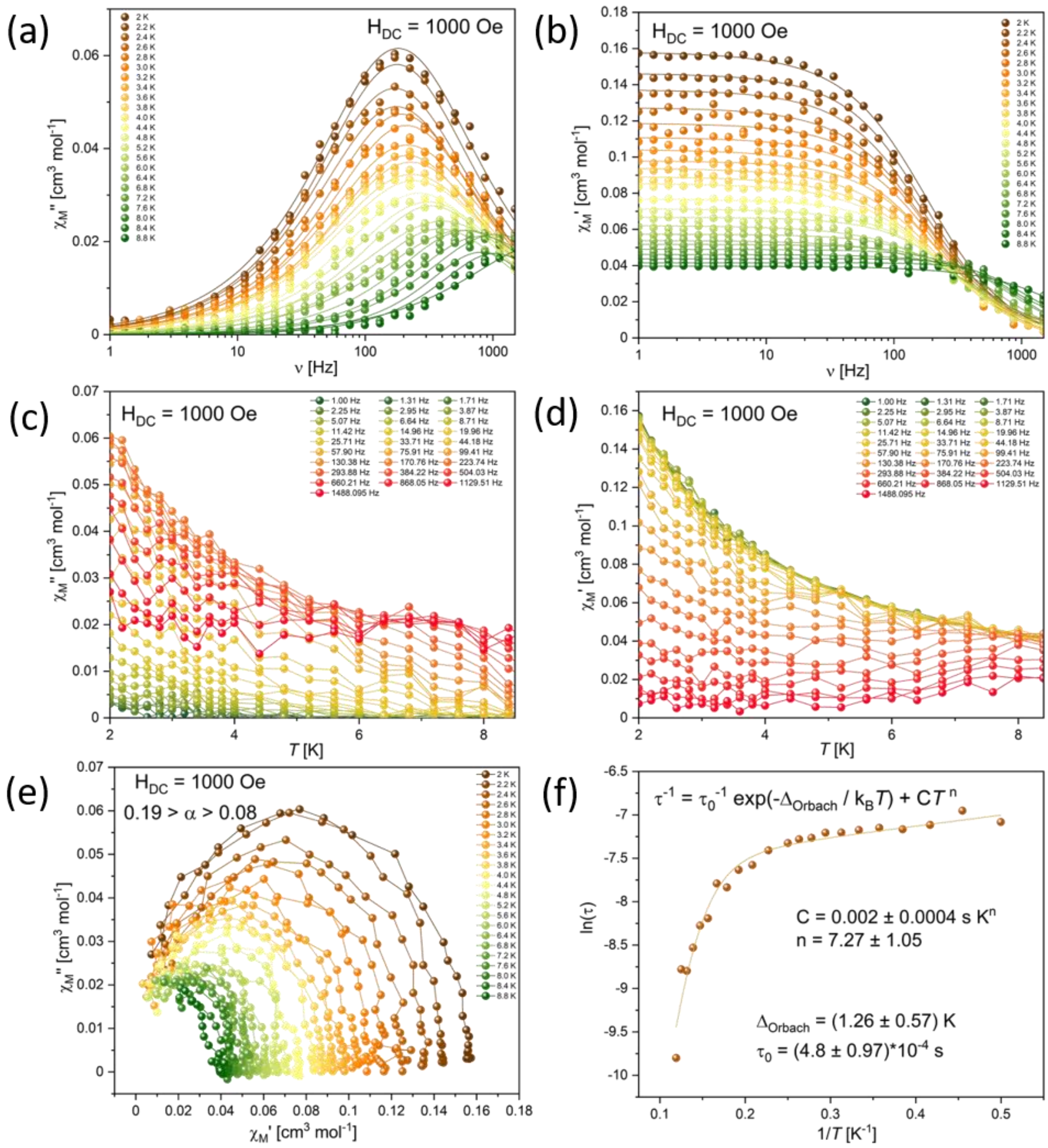

Figure S 40: Dynamic magnetic data for $[Ce^{III}(\eta^8\text{-}Cot^{TIPS})_2K]_n$ (**2-Ce**) employing a 3.5 Oe AC field and 1000 Oe DC field. (a) χ_M'' (v); b) χ_M'(v); (c) χ_M''(*T*); (d) χ_M'(*T*); (e) Cole-cole; (f) Arrhenius plot and best fit employing Orbach and Raman relaxation. For (a) and (b), the solid line are fit to a generalized debeye model. For (c), (d) and (e), the solid lines are a guide for the eye.

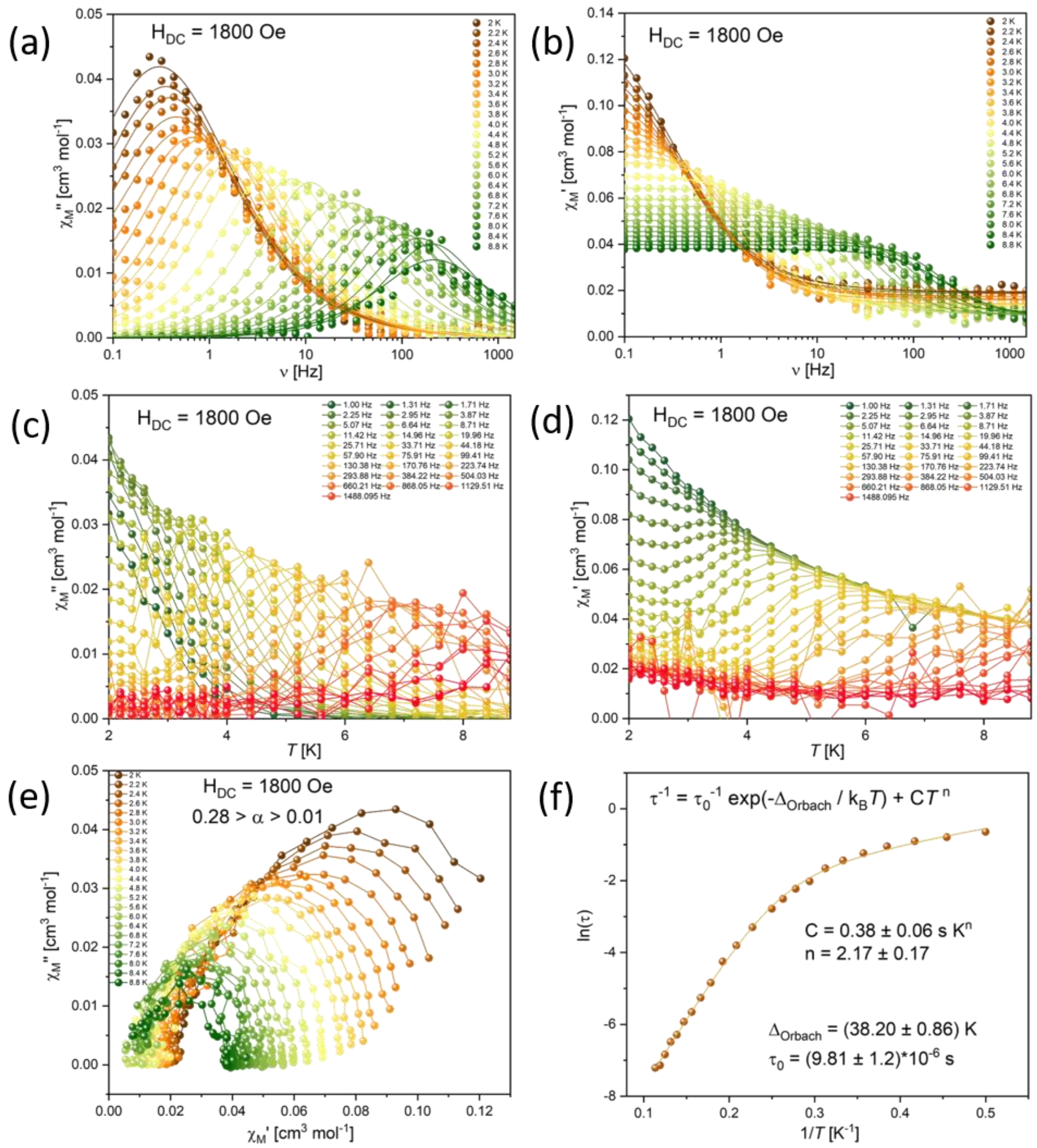

Figure S 41: Dynamic magnetic data for $[Nd^{III}(\eta^8\text{-Cot}^{TIPS})_2K]_n$ (**2-Nd**) employing a 3.5 Oe AC field and 1800 Oe DC field. (a) χ_M'' (ν); b) χ_M'(ν); (c) χ_M''(T); (d) χ_M'(T); (e) Cole-cole; (d) Arrhenius plot and best fit employing Orbach and Raman relaxation. For (a) and (b), the solid line are fit to a generalized debeye model. For (c), (d) and (e), the solid lines are a guide for the eye.

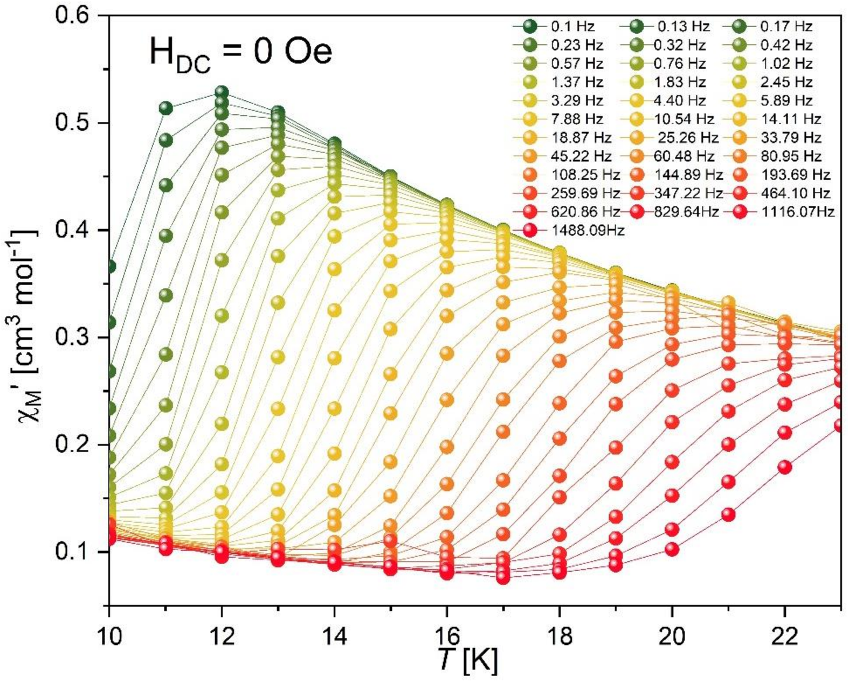

Figure S 42: In-Phase component of the magnetic susceptibility vs. temperature for $[Er^{III}(\eta^8\text{-}Cot^{TIPS})_2K]_n$ (**2-Er**). The solid lines are a guide for the eye.

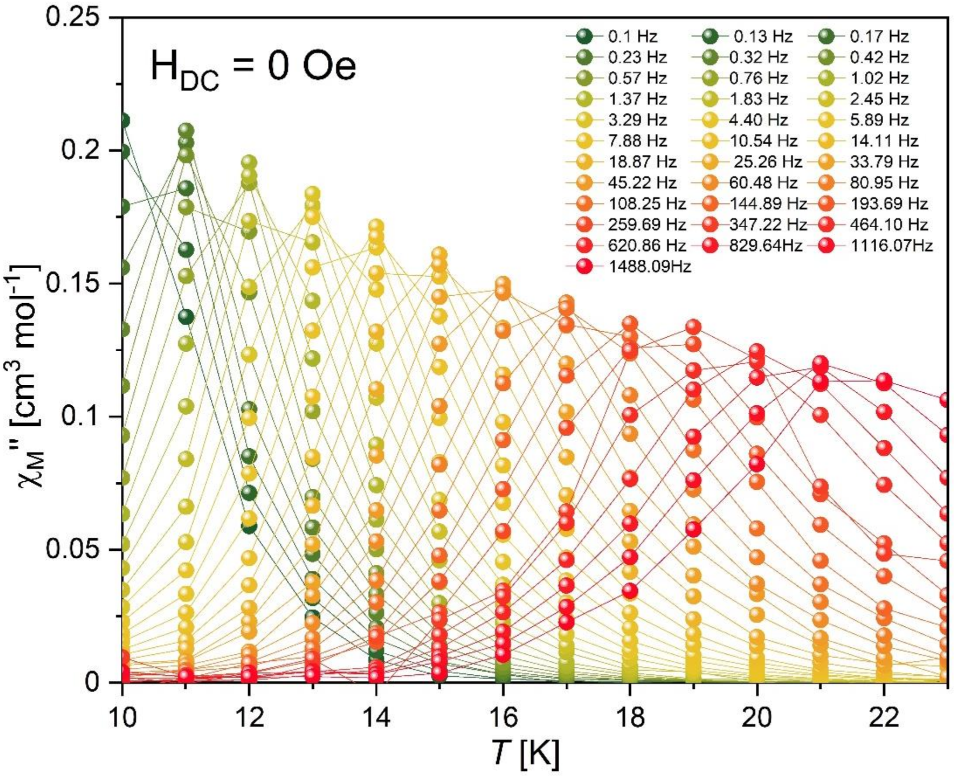

Figure S 43: Out-of-Phase component of the magnetic susceptibility vs. temperature for $[Er^{III}(\eta^8\text{-}Cot^{TIPS})_2K]_n$ (**2-Er**). The solid lines are a guide for the eye.

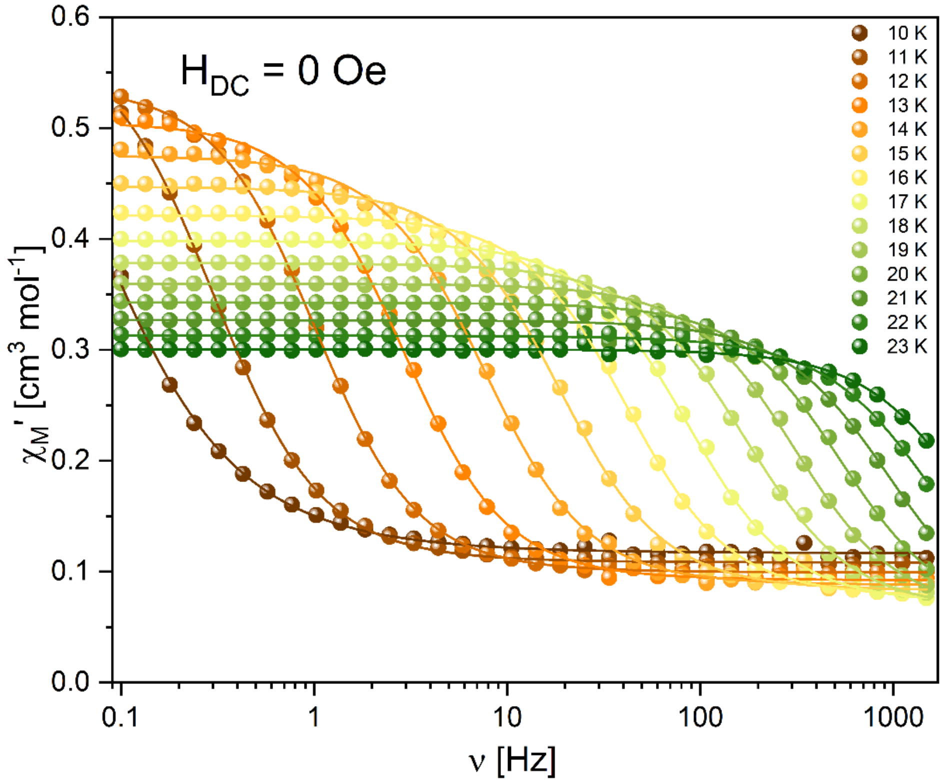

Figure S 44: In-Phase component of the magnetic susceptibility vs. AC frequency for [Er^{III}(η^8-Cot^{TIPS})$_2$K]$_n$ (**2-Er**). The solid lines are the fits to a generalized Debeye model.

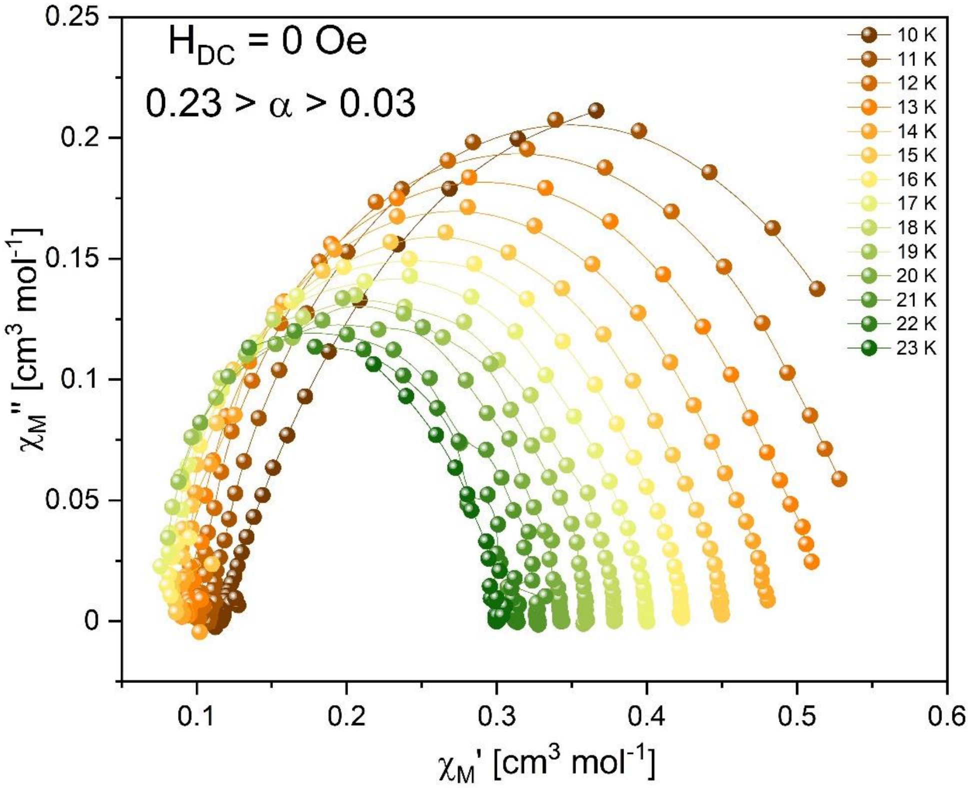

Figure S 45: Cole-Cole plot for [Er^{III}(η^8-Cot^{TIPS})$_2$K]$_n$ (**2-Er**). The solid lines are a guide for the eye.

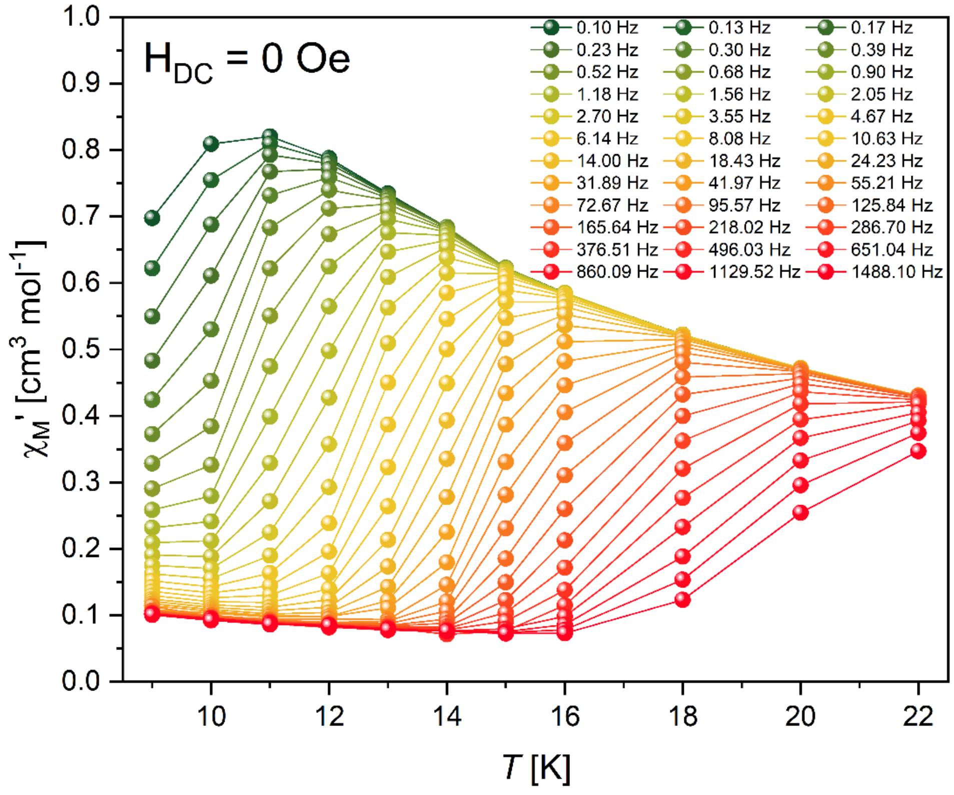

Figure S 46: In-Phase component of the magnetic susceptibility vs. temperature for $[Er^{III}(\eta^8\text{-}Cot^{TIPS})_2K]_n$ (**3-Er**). The solid lines are a guide for the eye.

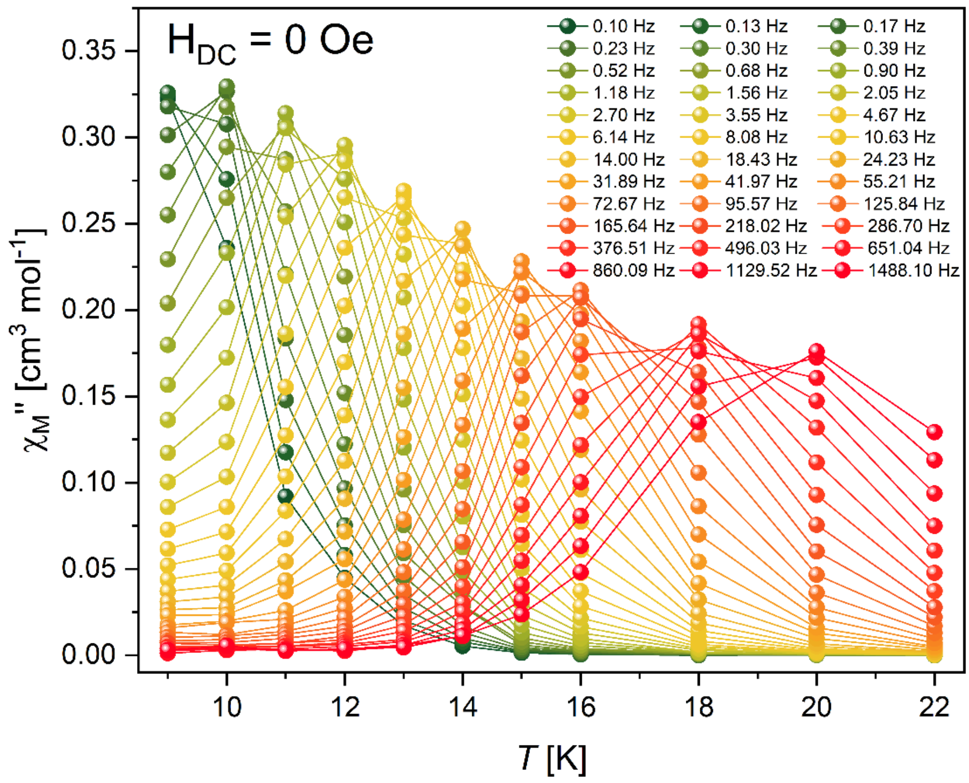

Figure S 47: Out-of-Phase component of the magnetic susceptibility vs. temperature for $[Er^{III}(\eta^8\text{-}Cot^{TIPS})_2K]_n$ (**3-Er**). The solid lines are a guide for the eye.

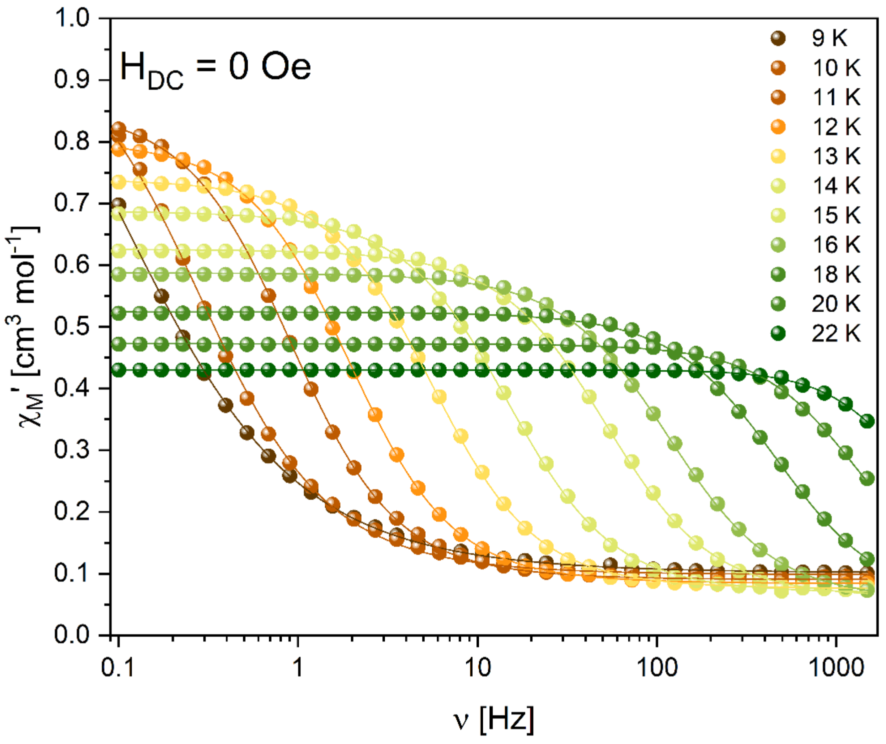

Figure S 48: In-Phase component of the magnetic susceptibility vs. AC frequency for $[Er^{III}(\eta^8\text{-}Cot^{TIPS})_2K]_n$ (**3-Er**). The solid lines are the fits to a generalized Debeye model.

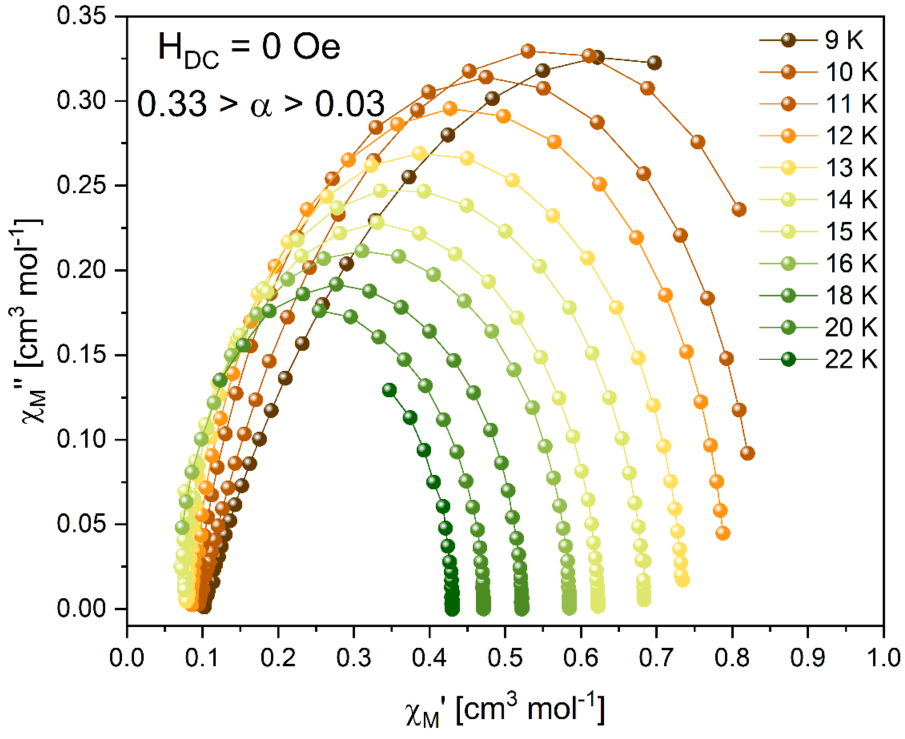

Figure S 49: Cole-Cole plot for $[Er^{III}(\eta^8\text{-}Cot^{TIPS})_2K]_n$ (3-Er). The solid lines are a guide for the eye.

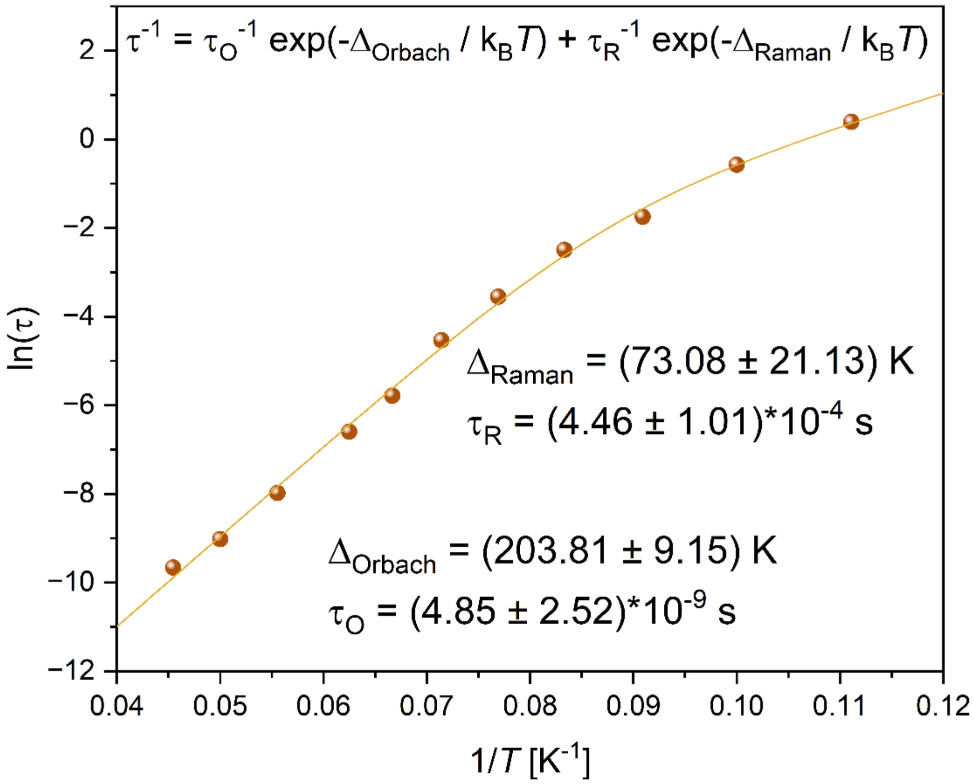

Figure S 50: Arrhenius plot and best fit employing Orbach and Raman relaxation for $[Er^{III}(\eta^8\text{-}Cot^{TIPS})_2K]_n$ (**3-Er**). The Raman process is treated using an exponential term.

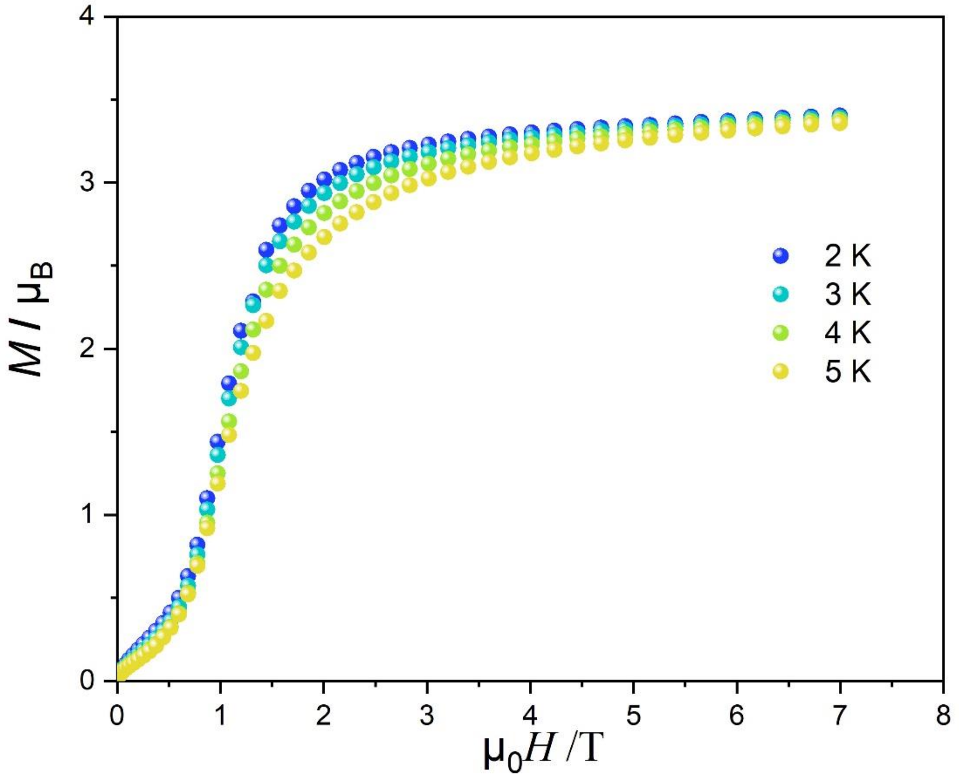

Figure S 51: Field-dependent magnetization obtained for for $[Er^{III}(\eta^8\text{-}Cot^{TIPS})_2K]_n$ (**2-Er**).

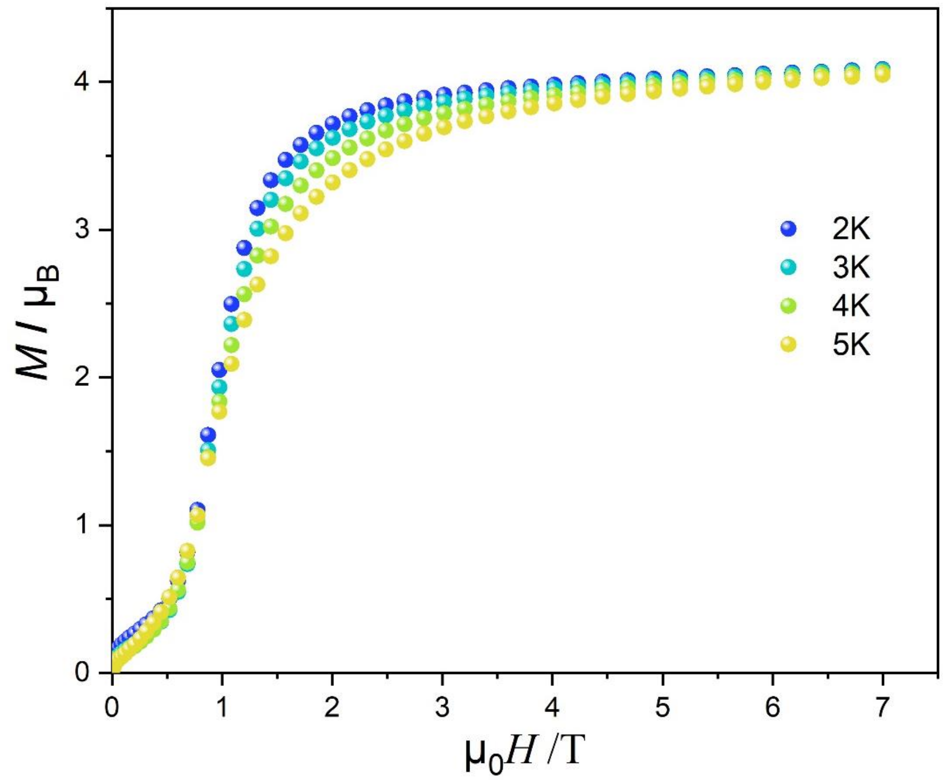

Figure S 52: Field-dependent magnetization obtained for for $[Er^{III}(\eta^8\text{-}Cot^{TIPS})_2K]_n$ (**3-Er**).

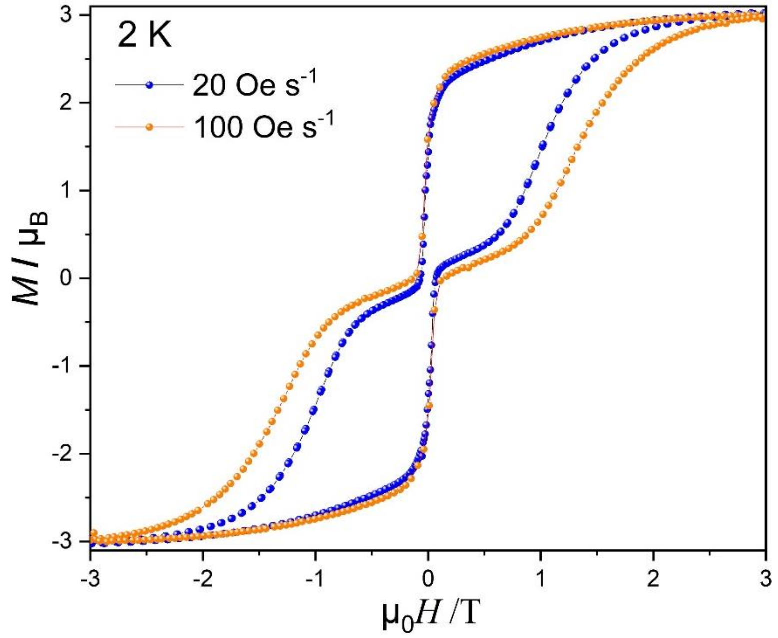

Figure S 53: Hysteresis at 2 K of $[Er^{III}(\eta^8\text{-}Cot^{TIPS})_2K]_n$ (**2-Er**).

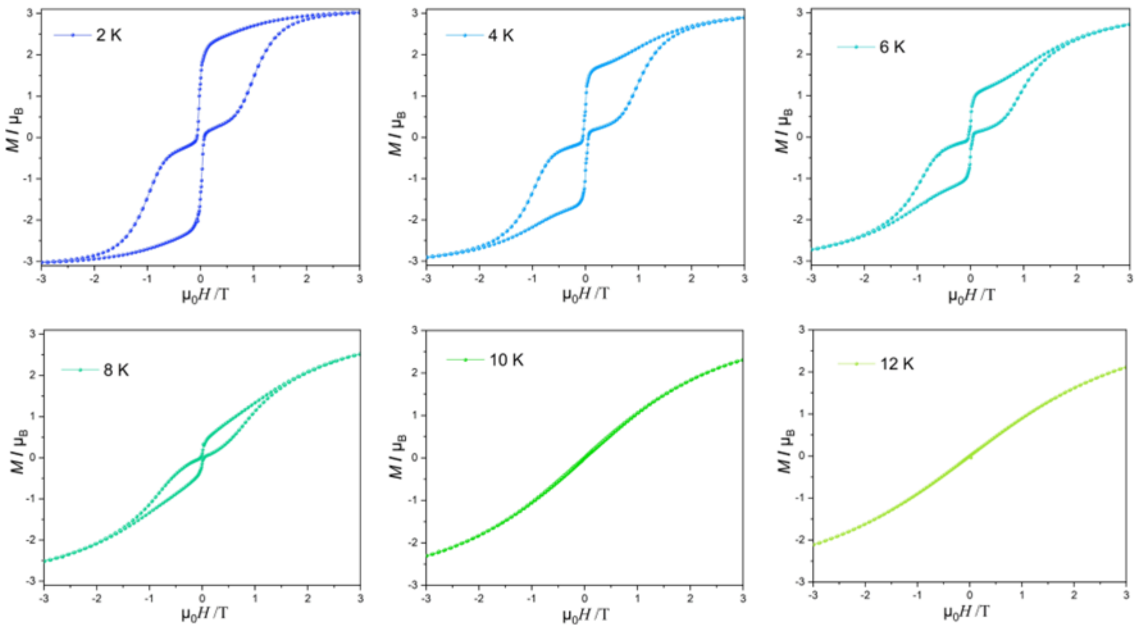

Figure S 54: Hysteresis at different T (20 Oe/s) of $[Er^{III}(\eta^8\text{-}Cot^{TIPS})_2K]_n$ (**2-Er**).

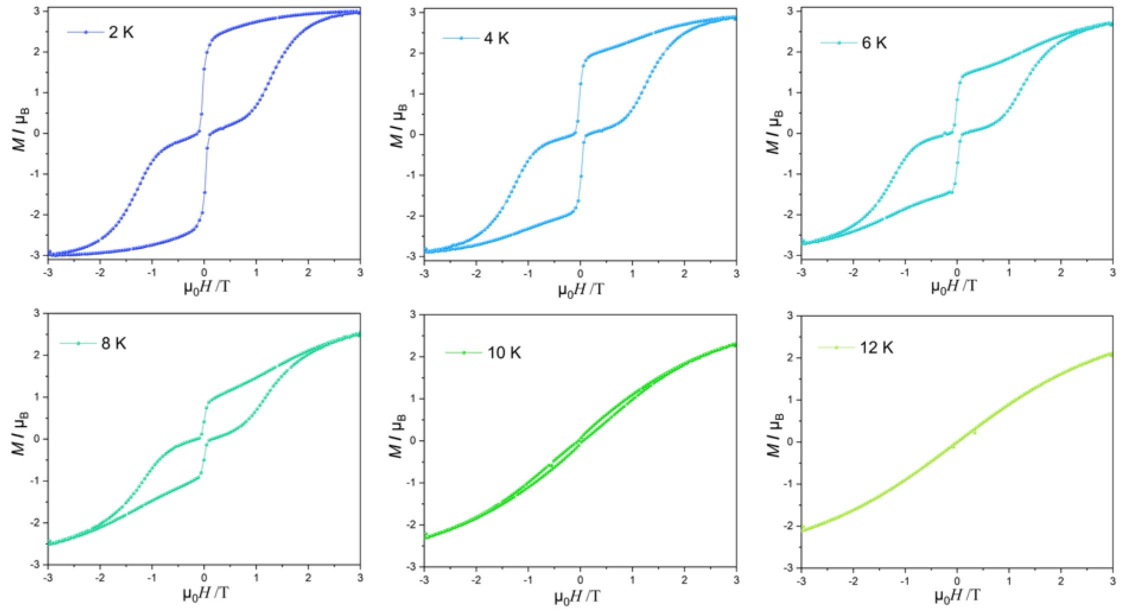

Figure S 55: Hysteresis at different T (100 Oe/s) of $[Er^{III}(\eta^8\text{-}Cot^{TIPS})_2K]_n$ (**2-Er**).

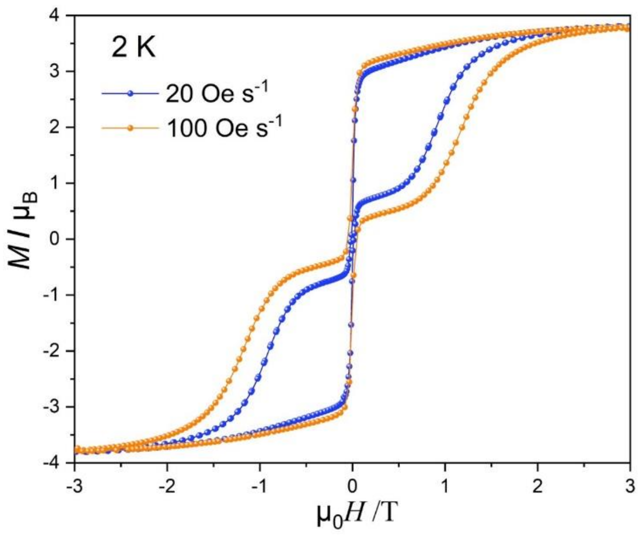

Figure S 56: Hysteresis at 2 K of $[Er^{III}(\eta^8\text{-}Cot^{TIPS})_2K]_n$ (**3-Er**).

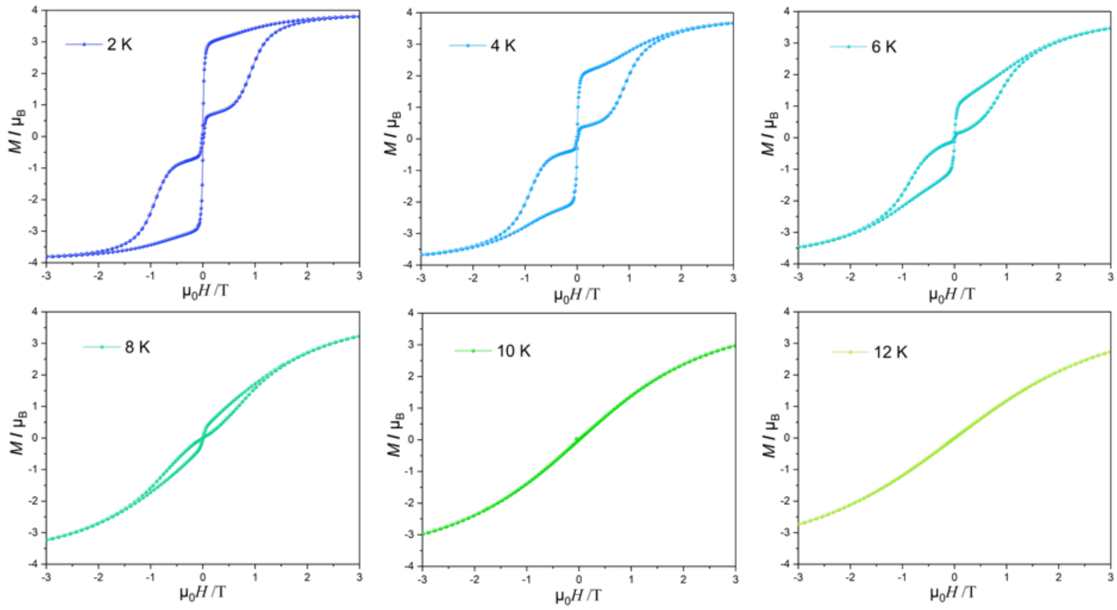

Figure S 57: Hysteresis at different T (20 Oe/s) of $[Er^{III}(\eta^8\text{-}Cot^{TIPS})_2K]_n$ (**3-Er**).

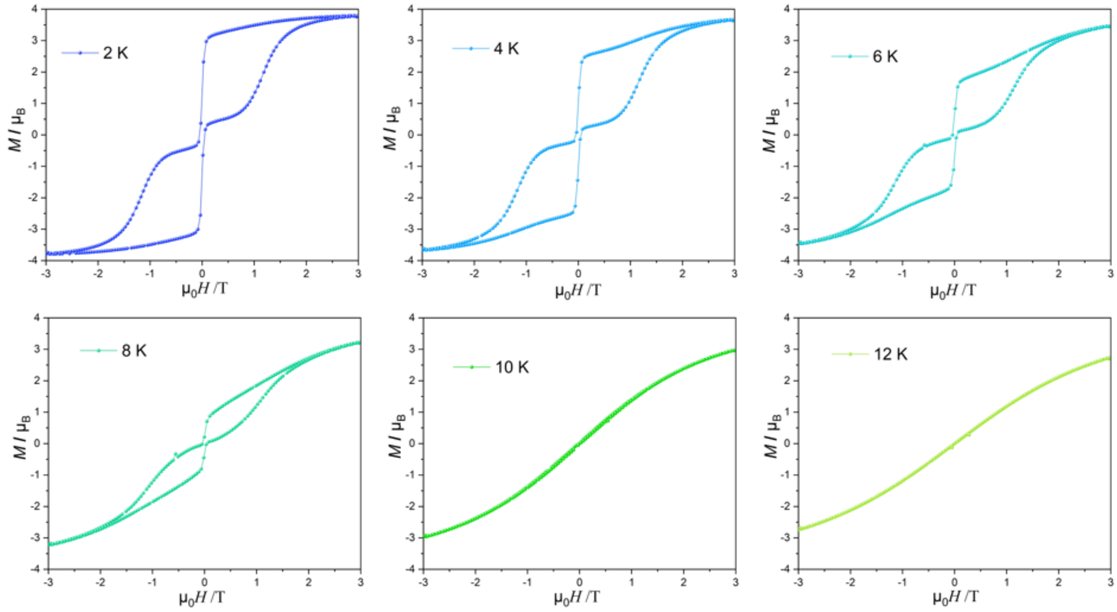

Figure S 58: Hysteresis at different T (100 Oe/s) of $[Er^{III}(\eta^8\text{-}Cot^{TIPS})_2K]_n$ (**3-Er**).

Ab initio calculations

A (11,7)-CASSCF calculation was carried out using the RASSCF/RASSI/SINGLE_ANISO routine of the OpenMolcas program package[6] on a fictional $[K_2ErY_2(COT^{TMS})_6]^-$ fragment of **2-Er** and **3-Er**. All basis sets taken, were of the relativistic ANO-RCC type. The size of the basis sets on the individual atoms were triple zeta with polarization for Er, double zeta with polarization for K as well as the 16 C's coordinating towards Er and double zeta for all other atoms. All 35 quartet and 112 doublet states were taken into the RASSI module.

Table S 17: Details of the J = 15/2 manifold obtained from CASSCF calculations for **2-Er**.

Doublet state	$\Delta E/cm^{-1}$	ΔE/K	g_x	g_y	g_z	Wavefunction composition
1	0	0	0.0000	0.0000	17.9438	∓ 100% |15/2›
2	164.90	237.24	0.0332	0.0346	15.4782	± 99% |13/2›
3	198.27	285.25	9.6730	9.3625	1.2599	∓ 71% |1/2› ± 28% |1/2›
4	267.17	384.37	0.0427	0.3594	3.6722	∓ 96% |3/2›± 3% |3/2›
5	364.05	523.76	0.0097	0.0252	13.0228	∓ 98% |11/2› ∓ 1% |5/2›
6	373.99	538.06	0.5245	0.9458	6.1084	± 88% |5/2› ∓ 9% |5/2› ± 1% |11/2›
7	459.15	660.58	0.4684	0.6803	10.8036	± 75% |7/2› ± 24% |9/2› ± 1% |3/2›
8	475.99	684.80	0.0811	0.1414	13.0574	± 75% |9/2› ± 24% |5/2›

Table S 18: Details of the J = 15/2 manifold obtained from CASSCF calculations for **3-Er**.

Doublet state	$\Delta E/cm^{-1}$	ΔE/K	g_x	g_y	g_z	Wavefunction composition
1	0	0	0.0001	0.0001	17.9432	∓ 100% |15/2›
2	165.30	237.83	0.0348	0.0365	15.4626	± 99% |13/2›
3	193.41	278.27	9.9285	9.1152	1.2604	∓ 99% |1/2›
4	262.09	377.09	0.3799	0.4393	3.6829	∓ 99% |3/2›
5	361.30	519.82	0.0071	0.0231	12.9319	∓ 97% |11/2› ∓ 2% |5/2› ∓ 1% |9/2›
6	367.66	528.97	0.5486	0.6338	6.2028	± 97% |5/2› ± 2% |11/2›
7	453.58	652.59	0.2998	0.5785	10.2109	∓ 84% |7/2› ∓ 15% |9/2›
8	468.84	674.55	0.1797	0.2591	12.4631	∓ 84% |9/2› ∓ 16% |7/2›

References

1 T. Summerscales Owen, F. G. N. Cloke, B. Hitchcock Peter, C. Green Jennifer, N. Hazari, *Science*, **2006**, 311, 829-831.

2 L. Münzfeld, X. Sun, S. Schlittenhardt, C. Schoo, A. Hauser, S. Gillhuber, F. Weigend, M. Ruben, P. W. Roesky, *Chem. Sci.*, **2022**, 13, 945-954.

3 G. Sheldrick, *Acta Crystallogr. Sect. A*, **2015**, 71, 3-8.

4 G. Sheldrick, *Acta Crystallogr. Sect. C*, **2015**, 71, 3-8.

5 O. V. Dolomanov, L. J. Bourhis, R. J. Gildea, J. A. K. Howard, H. Puschmann, *J. Appl. Crystallogr.*, **2009**, 42, 339-341.

6 F. Aquilante, J. Autschbach, A. Baiardi, S. Battaglia, V. A. Borin, L. F. Chibotaru, I. Conti, L. De Vico, M. Delcey, I. Fdez. Galván, N. Ferré, L. Freitag, M. Garavelli, X. Gong, S. Knecht, E. D. Larsson, R. Lindh, M. Lundberg, P. Å. Malmqvist, A. Nenov, J. Norell, M. Odelius, M. Olivucci, T. B. Pedersen, L. Pedraza-González, Q. M. Phung, K. Pierloot, M. Reiher, I. Schapiro, J. Segarra-Martí, F. Segatta, L. Seijo, S. Sen, D.-C. Sergentu, C. J. Stein, L. Ungur, M. Vacher, A. Valentini, V. Veryazov, *J. Chem. Phys.*, **2020**, 152, 214117.
